# Subcellular Localization of Extracytoplasmic Proteins in Monoderm Bacteria: Rational Secretomics-Based Strategy for Genomic and Proteomic Analyses

**DOI:** 10.1371/journal.pone.0042982

**Published:** 2012-08-09

**Authors:** Sandra Renier, Pierre Micheau, Régine Talon, Michel Hébraud, Mickaël Desvaux

**Affiliations:** INRA, UR454 Microbiology, Saint-Genès Champanelle, France; Institut Pasteur Paris, France

## Abstract

Genome-scale prediction of subcellular localization (SCL) is not only useful for inferring protein function but also for supporting proteomic data. In line with the secretome concept, a rational and original analytical strategy mimicking the secretion steps that determine ultimate SCL was developed for Gram-positive (monoderm) bacteria. Based on the biology of protein secretion, a flowchart and decision trees were designed considering (i) membrane targeting, (ii) protein secretion systems, (iii) membrane retention, and (iv) cell-wall retention by domains or post-translocational modifications, as well as (v) incorporation to cell-surface supramolecular structures. Using *Listeria monocytogenes* as a case study, results were compared with known data set from SCL predictors and experimental proteomics. While in good agreement with experimental extracytoplasmic fractions, the secretomics-based method outperforms other genomic analyses, which were simply not intended to be as inclusive. Compared to all other localization predictors, this method does not only supply a static snapshot of protein SCL but also offers the full picture of the secretion process dynamics: (i) the protein routing is detailed, (ii) the number of distinct SCL and protein categories is comprehensive, (iii) the description of protein type and topology is provided, (iv) the SCL is unambiguously differentiated from the protein category, and (v) the multiple SCL and protein category are fully considered. In that sense, the secretomics-based method is much more than a SCL predictor. Besides a major step forward in genomics and proteomics of protein secretion, the secretomics-based method appears as a strategy of choice to generate *in silico* hypotheses for experimental testing.

## Introduction

All living cells interface with their surrounding through proteins that are located in the cell envelope, displayed on the cell surface or released into the extracellular milieu, and even beyond when injected into a host cell. Such proteins are translocated in the first instance through biological membranes by protein-conducting channels, *i.e*. translocons. In bacteria, several secretion systems enabling protein transport from the inside to the outside of the cell have been characterised. Distinction must be made between bacteria possessing (i) one biological membrane (the cytoplasmic membrane), the so-called monodermata, and (ii) two biological membranes (the inner membrane and outer membrane), the so-called didermata [1], [2], [3]. Until recently and in contrast to diderm bacteria [4], [5], [6], [7], [8], comprehensive knowledge on protein secretion systems in monoderm species was restricted to the non-pathogenic *Bacillus subtilis* species [9], [10], [11] or scattered among different species for specific systems [12], [13], [14], [15].

Because of the absence of an outer membrane, the numerical classification of protein secretion systems does not apply to monodermata and export across the cytoplasmic membrane actually corresponds to a secretion event [16]. As in didermata, the Sec (secretion) and Tat (twin-arginine translocation) machineries are found in the cytoplasmic membrane but additional secretion systems can be present in monoderms, *i.e.* the FPE (fimbrilin-protein exporter), ABC (ATP-binding cassette) transporters, FEA (flagellum export apparatus), holins (hole-forming) and Wss (WXG100 secretion system) [17], [18]. As thoroughly explain by several specialists in the field of bacterial protein secretion [2], [19], [20], [21], [22], [23], [24], we will abstain to use the “T7SS” terminology to describe the Wss in monoderms, which is actually ascribed to the chaperone-usher pathway in diderm-LPS and at best only apply to diderm-mycolate, which is restricted to bacteria of the genus *Mycobacterium*.

As in any living cell, extracytoplasmic proteins cover a vast variety of functions, including nutrient uptake, chemosensing, motility, adhesion or cell envelope biogenesis. Moreover, their subcellular localization (SCL) and biological functions provide distinguishing clues regarding the physiology, lifestyle, position and interactions of the bacterial cell in an ecological niche and more generally in an ecosystem, as revealed for example by extracellular degradative enzymes in saprophytes or cell-surface virulence factors in pathogens. The final SCL of a protein results from a series of molecular mechanisms, involving post-translational and/or post-translocational modifications. The secretome concept is very useful for considering these different steps as it includes both the proteins secreted across the cytoplasmic membrane to the membrane–cell wall interface, the cell wall or the extracellular environment, and importantly, the secretory machineries themselves [10], [25], [26]. As such and contrary to what it sometimes misconceptualized by some authors, the secretome is not a proteome *per se*, let alone the subset of extracellular proteins, that is actually the exoproteome [1]. In bacteria with a Gram-positive cell-envelope architecture, the proteins actively transported *via* these secretion systems, the so-called secreted proteins, can have radically different final destinations and be either (i) anchored to the cytoplasmic membrane, (ii) associated with the cell wall, (iii) released into the extracellular milieu, or even (iv) injected into a host cell [1]. Description of SCL now follows the Gene Ontology (GO) recommendations for describing “Cellular component”, one of the three structured controlled vocabularies [27].

Because experimental investigation of the membrane and cell wall proteomes is hindered by technical limitation of protein extraction from their subcellular fractions, genomic prediction of SCL has been the subject of intense research effort. Numerous localization predictors have been developed for predicting the final destination of proteins. These bioinformatic tools can be divided into (i) specialized prediction tools, essentially based on the identification of signal peptides or retention sequences to the membrane or cell wall, *e.g.* SignalP [28], LipoP [29], TMHMM [30] or CW-PRED [31], and (ii) global prediction tools indicating the protein final SCL, *e.g.* PSORTb [32], LocTree [33], CELLO [34] or Gpos-mPLoc [35]. Such ensemble classifiers based on support vector machine (SVM) or neural network (NN) have been constructed on algorithms with a rationale somehow disconnected from the biology of the system investigated. Each of these tools having its own prediction limits, though, an alternative and powerful strategy consists in combining predictions [36]. For Gram-positive bacteria, different pipelines have been developed to predict final location of protein, *e.g.* Augur [37], LocateP [38] or SurfG+ [39], but none of them is comprehensive. A momentous limitation is that, by essence, their workflows are not evolutive but established once and for all and cannot be willingly adjusted in light of new findings in the field. Consequently, new specialized prediction tools cannot be swiftly implemented since any modifications remain at the discretion of their designers. In addition, results of some of these tools are frozen in databases and interrogations cannot be readily performed on demand or on newly available bacterial genomes.

In order to provide a much more comprehensive, reliable, flexible and adjustable prediction of protein SCL in monoderm bacteria, we aimed at developing a strategy for analysing genomic and proteomic data for secreted proteins. The originality of the method resides in the use of an extensive number of readily available bioinformatic tools organized in a workflow and decision trees mimicking very closely the molecular steps encountered by a protein in the course of secretion. Compared to all available predictions tools to date, this methodology embraces the secretome concept as it does not only consider the presence of various domains that can target or retain the secreted proteins within the cell-envelope of monoderm bacterium but most importantly considers the presence/absence of protein secretion pathways. Taking into account (i) it potentially expresses a prodigious amount of extracellular proteins and cell-surface proteins, which are membrane or cell-wall attached [4], [40], [41], (ii) it is a pathogenic Gram-positive bacterium contrary to the paradigm *B. subtilis*, and (iii) numerous experimental proteomic approaches have been dedicated to its extracytoproteome, including the surface and extracellular proteomes [42], [43], [44], [45], [46], [47], it prompts us to focus on the extracytoproteome of *Listeria monocytogenes* as a case study.

## Results

### Design of the secretomics-based method for genomic analysis of secreted proteins in monoderm bacteria

**Figure 1:**
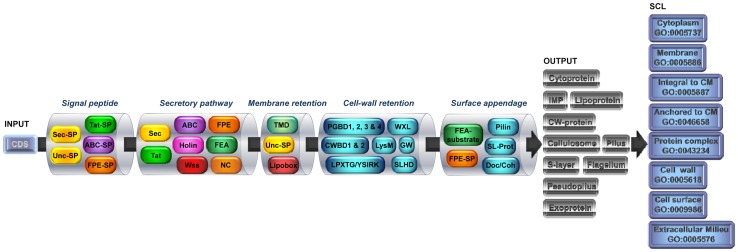
Synoptic view of the secretomics-based method for monoderm bacterium. Based on the biology of protein secretion, the coding sequences (CDS) are sequentially analysed in a workflow for (i) signal peptide (SP), (ii) secretory pathway, (iii) membrane retention, (iv) cell-wall retention, and (v) surface appendage. For each step, a combination of different tools allows defining different databases, as indicated in the detailed flowchart (**Figure 2**). From there, the resulting databases are analysed as depicted in the detailed decision trees (**Figure 3**). In the end, proteins are discriminated into different categories and different SCL are predicted. Sec-SP: Sec-dependent SP; Unc-SP: uncleaved SP; TMD: transmembrane domain; PGBD: peptidoglycan-binding domain; CWBD: cell-wall binding domain; SLHD: S-layer homology domain; SL-Prot: S-layer protein; Doc/Coh: dockerin/cohesin domain; IMP: integral membrane protein; GO: gene ontology.

The rationale for analyzing the final SCL of secreted proteins is based on the biology of protein secretion, *i.e.* the secretome. This concept provides an integrated and global view by considering protein routing, transport systems, post-translocational (translational) modifications and subcellular location [10], [25], [26]. Based on the secretome in monoderm bacteria (**Figure 1**), the steps considered are (i) the targeting of protein to the membrane by an N-terminal signal peptide, (ii) the protein secretion systems present, *i.e.* Sec, Tat, ABC, FPE, FEA, holin and/or Wss, (iii) the membrane retention of secreted proteins by domains or post-translational modifications, (iv) the cell-wall retention of secreted proteins by domains or post-translational modifications, and (v) the incorporation to cell-surface supramolecular structures. A summary of the different abbreviations in use in relation to protein secretion, especially protein categories and subcellular localizations, is provided in **Table 1** **.**

**Table 1:** Summary of the abbreviations in use in relation to protein secretion.

Abbreviation	Full name
*Protein categories*	
IMP	integral membrane protein
ssIMP I/II/III	single-spanning IMP of type I/II/III
msIMP	multi-spanning IMP
CW-protein	parietal protein
*Subcellular localization (SCL)*	
CM	cytoplasmic membrane
CW	cell wall
CS	cell surface
EM	extracellular milieu
*Other*	
SP I/II	signal peptide of type I/II
Unc-SP	uncleaved SP
TMD	α-helical transmembrane domain
CWBD1/2	cell-wall binding domain of type 1/2
PGBD1/2/3/4	peptidoglycan-binding domain of type 1/2/3/4
SLHD	S-layer homology domain
Sec	secretion
Tat	twin-arginine translocation
FEA	flagellum export apparatus
FPE	fimbrillin-protein exporter
ABC	ATP-binding cassette
Wss	WXG100 secretion system
NC	non-classical secretion

**Figure 2:**
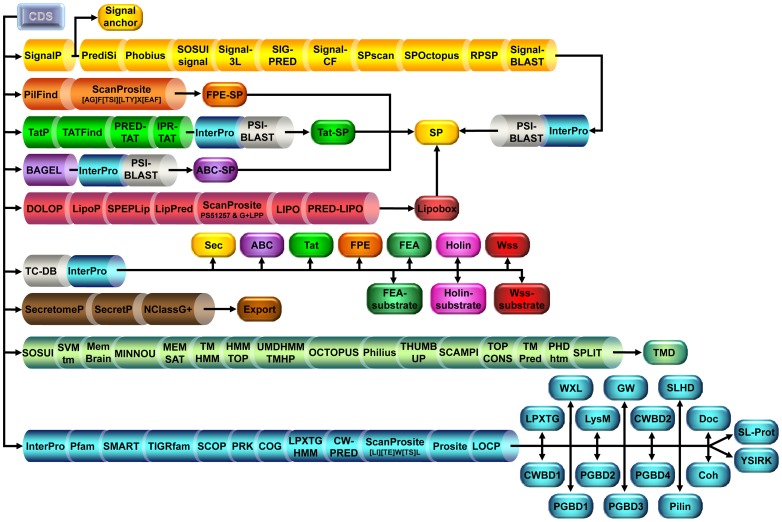
Comprehensive flowchart of the secretomics-based method in a monoderm bacterium. The analysis considered the (i) signal peptide (SP), (ii) type of SP (Signal anchor, FPE-SP, Tat-SP, ABC-S and, lipobox), (iv) protein secretion systems, (v) exported proteins lacking a SP (Export, FEA-, Holin, and Wss-substrates), (iv) transmembrane domain (TMD), and (iv) relevant conserved domains (LPXTG, WXL, LysM, CWBD1,...*etc*). Details of the prediction tools used for the analysis and definition of the databases are provided inthe Materials & Methods section.

**Figure 3:**
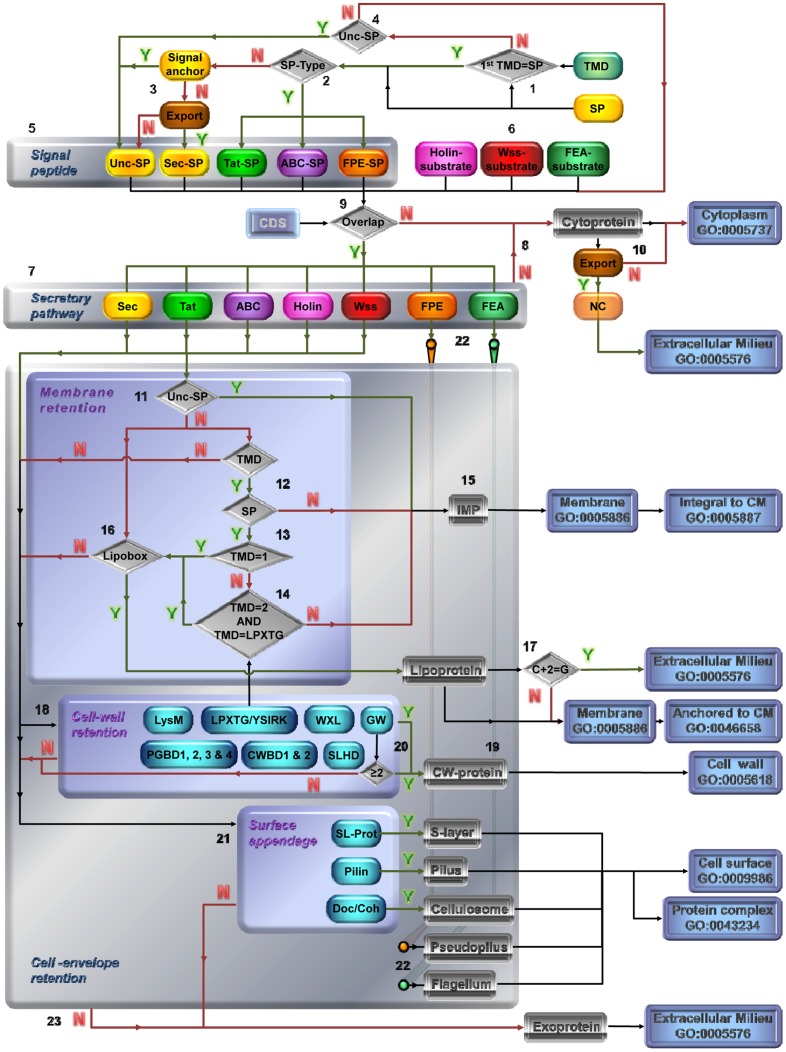
Detailed decision trees for prediction of protein category and SCL of secreted proteins. (1) Proteins exhibiting N-terminal SP are extracted from TMD database (Y). (2) The different types of SP (Tat-SP, ABC-SP and FPE-SP) are extracted (Y). (3) Absence of a signal anchor (N) and export (Y) define Sec-dependent SP (Sec-SP). (1) Proteins with TMD but no predicted SP (N), (4) are checked for uncleaved SP (Unc-SP), *i.e.* TMD of at least 7 amino acid within the first 100 N-terminal residues and with N_in_-C_out_ topology (Type II signal) (Y). (3) Unc-SP also comprises proteins with signal anchor (Y) and SP categorised as non-exported (N). (5) From the types of SP are clearly defined. Together with (4) proteins with TMD but no SP (N) and (6) protein substrates of holins, Wss and FEA, (7) the presence of the respective protein secretion systems is checked (Y). (8) When the respective protein secretion system is absent (N) or (9) proteins are not predicted as secreted (N), proteins are considered as cytoproteins and located in the CP. (10) Cytoproteins predicted as exported by NC (Y) are further considered as located extracellularly. (7) Secreted proteins and their respective secretion system are defined from there. (11) Translocated proteins with Unc-SP (Y) are IMPs. (11) Translocated proteins without Unc-SP (N), and (12) with TMD (Y) but no SP (N) are IMPs. (13) Remaining translocated proteins with a cleavable SP (Y) and a single predicted TMD (TMD = 1) (Y) cannot be IMP, and are checked for (16) the presence of a lipobox. (13) Remaining translocated proteins with more than one TMD (N) are checked for (14) the absence of overlap (N) with SP region and LPXTG domain respectively (TMD = 2 AND TMD = LPXTG) to be IMP, otherwise (Y) are checked for (16) the presence of a lipobox. (15) From TMD topology prediction (**Figure 2**), IMPs are further subcategorised and considered as integral to CM. (16) Presence of a lipobox (Y) define lipoproteins anchored to the CM. (17) The presence of glycine residue at position C+2 (C+2 = G) (Y) indicates potential release into the EM [26], [48]. (18) Presence of cell-wall retention domains define (19) parietal proteins (CW-protein) that are further subcategorised (**Figure 2**) and considered as located at the CW. (20) Proteins with less tha 2 GW modules is not defined as CW-protein located at the CW [57], [58]. (21) Proteins part of S-layer, pilus and cellulosome, as well as (22) pseudopilus and flagellum are defined. (23) Secreted proteins with none of the cell-envelope retention are as exoproteins located in the EM. N: No, Y: Yes.

As one of the cornerstones of the secretomics-based method, the type of signal peptide (SP) is defined (**Figure 2**). Besides, the search in parallel of all known protein secretion pathways in monoderm bacteria constitutes the keystone of this analytical strategy. Secretion pathways not only focus on translocases of the secretion systems but include the associated post-translocation maturation pathways, namely the respective signal peptidases, the lipoprotein maturation pathway, and the covalent cell-wall anchoring sortase pathway (**Table 2**). It is important to stress that the absence of a SP cannot rule out protein secretion by alternative systems as detailed below. Presence of the different types of SP is associated with the respective secretion systems, whereas substrates to alternative secretion systems lacking SP are subjected to similarity search (**Figure 3**). Together with pseudopilus and flagellum secreted *via* FPE and FEA respectively, other surface supramolecular structures are considered, namely S-layer, pilus and/or cellulosome. For integral membrane protein (IMP) and besides the presence of transmembrane domain (TMD), the presence of uncleaved N-terminal SP, which could serve as a signal anchor, is utterly considered. Secreted proteins, which are neither predicted as retained to the membrane, cell-wall nor subunits of surface appendages, are predicted as exoproteins. Once the different protein categories are defined, SCL can be predicted (**Figure 3**). Cytoproteins potentially secreted by non-classical pathway (NC) are also predicted as localized in the extracellular milieu. Position +2 of the cleavage site (C+2) is checked as an indicator that the lipoprotein can be potentially released into the extracellular milieu [26], [48]. The number of GW modules is carefully considered for protein SCL in the extracellular milieu rather than in the cell wall. Besides discriminating the protein category from the SCL to avoid any confusing statement or misleading interpretation, the secretomics-based method provides a detailed description of protein routing and permits considering multiple SCL for a given protein.

**Table 2:** Protein secretion pathways in *L. monocytogenes* EGD-e as revealed by the secretomics-based method.

Secretion pathway^a^	Protein ID	Annotation^b^	Similarity search^c^
*Sec*			
Translocase	Lmo2612	Sec translocon, subunit SecY	TC#3.A.5, COG0201, IPR002208, PIRSF004557, TIGR00967, SSF103491, PF00345
	Lmo0245	Sec translocon, subunit SecE	TC#3.A.5, COG0690, IPR001901, IPR005807, TIGR00964, PF00584
	Lmo2451	Sec translocon, subunit SecG	TC#3.A.5, COG1314, IPR004692, TIGR00810, PF03840
	Lmo1527	Sec transcolon, bifunctional subunit SecDF	TC#2.A.6.4, TC#3.A.5, IPR03335, PF07549, PF02356
	Lmo1529	Sec transcolon, subunit YajC	TC#9.B.18, COG1862, TIGR00739, PF02699
	Lmo2510	Sec translocase, ATPase, SecA	TC#3.A.5, COG0653, IPR000185, TIGR00963
	Lmo0583	Sec translocase, ATPase, SecA2	TC#3.A.5.10, COG0653
	Lmo1803	Signal recognition particle (SRP) receptor subunit, FtsY	TC#3.A.5, COG0552, IPR004390, IPR000897, TIGR00064, SSF47364
	Lmo1801	Signal recognition particle (SRP), Ffh	TC#3.A.5, COG0541, IPR004780, TIGR00959, SSF47446
Insertase	Lmo1379	YidC insertase, OxaA1 (YqjG)	TC#2.A.9, COG0706
	Lmo2854	YidC insertase, OxaA2 (SpoIIIJ)	TC#2.A.9, COG0706
SPase	Lmo1269	Signal peptidase of Type I, SipX	COG0681, IPR000223, TIGR02227, SSF51306
	Lmo1270	Signal peptidase of Type I, SipY	COG0681, IPR000223, TIGR02227, SSF51306
	Lmo1271	Signal peptidase of Type I, SipZ	COG0681, IPR000223, TIGR02227, SSF51306
	Lmo1844	Signal peptidase of Type II, lipoprotein signal peptidase, LspA	COG0597, IPR001872, TIGR00077, PF01252
	Lmo1101	Signal peptidase of Type II, lipoprotein signal peptidase, LspB	COG0597, IPR001872, TIGR00077, PF01252
CM anchoring	Lmo2482	Prolipoprotein diacylglyceryltransferase, Lgt	COG0682, IPR001640, TIGR00544, PF01790
CW anchoring	Lmo0929	Sortase A, SrtA	COG3764, IPR005754, TIGR01076, SSF63817, PF04203
	Lmo2181	Sortase B, SrtB	COG4509, IPR009835, IPR015986, PIRSF030150, TIGR03064, SSF63817, PF07170
*Tat*			
	Lmo0362	Twin-arginine translocase protein A, TatA	TC#2.A.64, COG1826, IPR003369, IPR006312, TIGR01411, PF02416
	Lmo0361	Twin-arginine translocation protein, TatC	TC#2.A.64, COG0805, IPR002033, TIGR00945, PF00902
*ABC*			
	Lmo0607	ABC-type bacteriocin exporter, peptidase domain, ATP-binding/permease protein	TC#3.A.1, COG2274
	Lmo0608	ABC-type bacteriocin exporter, peptidase domain, ATP-binding/permease protein	TC#3.A.1, COG2274
	Lmo2580	ABC-type antimicrobial peptide transport system, ATPase component	TC#3.A.1, COG1136
	Lmo2581	ABC-type antimicrobial peptide transport system, permease component	TC#3.A.1, COG0577
	Lmo2751	ABC-type bacteriocin exporter, peptidase domain, ATP-binding/permease protein	TC#3.A.1, COG2274
	Lmo2752	ABC-type bacteriocin exporter, peptidase domain, ATP-binding/permease protein	TC#3.A.1, COG2274
	Lmo0107	ABC-type bacteriocin exporter, peptidase domain, ATP-binding/permease protein	TC#3.A.1, COG2274
	Lmo0108	ABC-type bacteriocin exporter, peptidase domain, ATP-binding/permease protein	TC#3.A.1, COG2274
*FPE*			
	Lmo1347	Fimbrilin-protein exporter, ATPase component, ComGA	TC#3.A.14
	Lmo1346	Fimbrilin-protein exporter, membrane component, ComGB	TC#3.A.14
	Lmo1550	Type 4 prepilin peptidase, ComC	COG1989, IPR000045, PF01478
*FEA*			
	Lmo0680	Flagellar export apparatus, membrane subunit FlhA	TC#3.A.6, COG1298, IPR001712, PF00771
	Lmo0679	Flagellar export apparatus, membrane subunit FlhB	TC#3.A.6, COG1377, IPR006135, PF01312
	Lmo0678	Flagellar export apparatus, membrane subunit FliR	TC#3.A.6, COG1684, IPR002010, PF01311
	Lmo0677	Flagellar export apparatus, membrane subunit FliQ	TC#3.A.6, COG1987, IPR002191, PF01313
	Lmo0676	Flagellar export apparatus, membrane subunit FliP	TC#3.A.6, IPR018035, PF02108
	Lmo0715	Flagellar export apparatus, peripheral subunit FliH	TC#3.A.6, COG1338, IPR005838, PF00814
	Lmo0716	Flagellar export apparatus, ATPase subunit FliI	TC#3.A.6, COG1157, IPR005714, TIGR01026
*Holin*			
	Lmo0128	Holin TcdE-like	TC#1.E, COG4824, IPR006480, TIGR01593, PF05105
	Lmo2279	Holin phage A118	TC#1.E, IPR009708, PF06946
*Wss*			
	Lmo0061	WXG100 secretion system, ATPase component, YukAB (EssC)	TC#9.A.44, IPR023839, TIGR03928
	Lmo0057	WXG100 secretion system, membrane component, EsaA	TC#9.A.44, IPR023838, TIGR03929
	Lmo0058	WXG100 secretion system, membrane component, EssA	TC#9.A.44, IPR018920, PF10661, TIGR03927
	Lmo0060	WXG100 secretion system, membrane component, YukC (EssB)	TC#9.A.44, IPR018778, PF10140, TIGR03926
	Lmo0059	WXG100 secretion system, peripheral component, YukD (EsaB)	TC#9.A.44, IPR14921, PIRSF037793, PF08817
	Lmo0062	WXG100 secretion system, peripheral component, EsaC	TC#9.A.44

a Protein secretion systems: Sec (Secretion), Tat (Twin-arginine translocation), ABC (ATP-binding cassette), holin (hole forming) and Wss (WXG100 secretion system) pathways.

b Some annotations were corrected respective to the similarity search performed as described in the Material & Methods section. More extensive and detailed annotations are available in Table S1.

c Similarity search were based on interrogations of dedicated databases as described in the Material & Methods section.

### SCL prediction of secreted proteins in *L. monocytogenes* following the secretomics-based method

Refining the analysis of genome-encoded proteins exhibiting a SP in *L. monocytogenes* EGD-e and consolidating the results with the prediction of uncleaved SP, it appears that 723 proteins exhibit a SP, *i.e.* 1 protein with a Tat-SP, 3 an ABC-SP and 5 a FPE-SP as well as 224 with a SP of Type I (SP I), 74 with a SP II and 416 with an uncleaved SP (Unc-SP) (Table S1). The protein secretion pathways (**Table 2**) are associated with the identification of their respective protein substrates. Altogether and following majority vote approach, 741 proteins appear secreted, including 714 proteins targeted to Sec, 1 to Tat, 4 to ABC (including a leaderless bacteriocin), 3 to holin, 3 to Wss, 5 to FPE and 11 to FEA (Table S1). In addition, 162 IMPs lack a SP but could nonetheless be targeted and translocated within the membrane *via* YidC. Moreover, 108 proteins primarily predicted as cytoproteins, were further predicted as putatively secreted by NC. Altogether, proteins secreted *via* these different pathways are predicted as either located to the cytoplasmic membrane (CM; GO:0031226), cell wall (CW; GO:0009275), cell surface (CS; GO:0009986) and/or extracellular milieu (EM; GO:0005576).

#### Location intrinsic to the cytoplasmic membrane (GO:0031226)

This location splits into two further classes, i.e. either integral to the cytoplasmic membrane (GO:0005887) or anchored to the cytoplasmic membrane (GO:0046658).

Among the 686 predicted IMPs (GO:0005887) as revealed by majority-vote scheme, 524 bear an N-terminal SP including 100 with a SP I, 8 with a SP II and 416 with an Unc-SP (Table S2). From the most recent review [40], the number of protein with a C-terminal “hydrophobic tail” was estimated at only 10, no estimate have ever been provided for N-terminal “hydrophobic tail” proteins [49]. It is also worth noting that the view that only single-spanning IMP (ssIMP) with N-terminal or C-terminal TMD can be considered as surface exposed is simplistic. On the one hand, it is not only the TMD position but rather its orientation that should be considered. On the other hand, even a short strand of amino acid interacting with the external side, such as a loop in multi-spanning IMP (msIMP) can have significant biological function. Based on topogenic elements defined by TMD orientation, where Type I and Type II modules have N_out_–C_in_ and N_in_–C_out_ orientations respectively [50], ssIMPs are further classified into (i) Type I where the ssIMP remains membrane-integrated by a Type I module following cleavage of an N-terminal SP, (ii) Type II where the ssIMP remains membrane-integrated by a Type I module, such an Unc-SP for instance, and (iii) Type III where the ssIMP remains membrane-integrated by a Type I module but is deprivated of an N-terminal SP [51], [52], [53]. Following the standard nomenclature and among the 157 ssIMP, 20 are of Type I (ssIMP I), *i.e.* exhibiting a cleavable SP, 120 are ssIMP II (including 87 exhibiting an Unc-SP), and 17 ssIMP III (Table S2). Among the 529 ms IMP (msIMP), 88 exhibit a cleavable Sec-SP and 323 an Unc-SP (Table S2). From literature survey, the number of IMP in *L. monocytogenes* was estimated at 267 [4], proteins with a C-terminal “hydrophobic tail” was estimated at only 10 [40] and no estimate had ever been provided for N-terminal “hydrophobic tail” proteins [49]. Besides the fact that the terminology in usage for IMPs in *L. monocytogenes* is quite inadequate, the estimate is here considerably changed and qualitatively improved.

Among the 74 lipoproteins (GO:0046658) predicted through majority voting (Table S3), 41 exhibited a glycine residue at position +2 of the cleavage site, conferring them a double possible location in the CM and the EM. Besides, 8 lipoproteins are also predicted as IMP (2 ssIMP I and 6 msIMP); 5 of them (Lmo0269, Lmo0641, Lmo0821, Lmo2687 and Lmo2793) are predicted as lipoproteins for the first time. While one-third of them have unknown function, even after PSI-BLAST search, the majority of them would be substrate-binding protein from ABC transport system. The combination of comprehensive prediction tools allowed here correcting the previous prediction of 68 lipoproteins [40].

#### Location at the cell wall (GO:0009275)

Parietal proteins (CW-protein) covalently anchored to the cell-wall bear a C-terminal LPXTG domain. Of note, the LPXTG-domain refers to the original regular expression of the motif now surpassed by HMM profiles encompassing the diversity of the cell-wall covalent-anchoring domain, *i.e.* the LPXTG-protein family [54]. In *L. monocytogenes*, the 43 LPXGT-proteins previously mentioned have been here predicted [40], [49], including two LPXTG-proteins substrate to SrtB, a lipoprotein (Lmo1136) and a LXPTG protein bearing LysM domains (Lmo880) (Table S4). No YSIRK motif (IPR005877) could be identified within Sec-dependent signal peptides of these proteins; when present, this motif is systematically associated with a LPXTG domain for efficient protein secretion [55] and/or specific final localization within the bacterial cell wall [56].

Besides proteins covalently linked to the cell wall, several CW-proteins are predicted attached by alternative means (Table S4). Contrary to the most recent review [40], only 5 instead of 9 GW-proteins are estimated as CW-proteins. Indeed, the 4 other proteins only exhibit one GW module, which is insufficient for binding to lipoteichoic acids in a noncovalent manner, and are consequently considered here as located extracellularly (Table S6) [57], [58], [59]. In addition, 4 WXL-proteins and one PGBD1-protein (Lmo1851) have been also identified. Previously, the PGBD1-protein had been announced as a IMP because of the presence of a TMD [40]; however, the only TMD predicted is located in the cleavable N-terminal signal peptide of Type I.

#### Location at cell surface (GO:0009986)

According to GO, this term is intended to annotate gene products that are attached to the CM (intrinsic or loosely bound, GO:0031226 and GO:0031232, respectively) or to the CW (integral or loosely bound, GO:0009275 and GO:0010339, respectively), and thus not only covers IMP, lipoprotein and CW-protein categories but also includes protein complexes (GO:0043234).

Concerning secreted proteins belonging to supramolecular cell-surface appendages (GO:0043234 AND 0009986), neither S-layer proteins, cellulosome components nor prepilins with LPXTG domains could be identified in *L. monocytogenes.* Nonetheless, 11 flagellar components are predicted secreted and assembled by the FEA (Table S5). In addition, 5 genes encoding for pseudo-prepilin exported by the FPE are identified. Surprisingly, these supramolecular structures had rarely been reported as part of the surface proteins in *L. monocytogenes* [40], [60].

#### Location into the extracellular milieu (GO:0005576)

80 exoproteins were predicted as localized extracellularly, including 69 proteins transported *via* Sec, 1 protein secreted *via* Tat, 4 bacteriocins translocated *via* ABC transporters, 3 proteins exported *via* holins and 3 proteins secreted *via* Wss (Table S6). While three proteins were predicted with a Tat-SP in the first place (Table S1), only the Dyp (dye decolorising peroxydase)-type peroxydase Lmo0367 was finally predicted as a Tat substrate (Table S6). Among the five predicted bacteriocins, four of them would be secreted *via* ABC transporters, *i.e.* Lmo0335, Lmo0615, Lmo2574, and Lmo2753, which is leaderless. As a lactococcin 972 homolog Lmo2776 exhibits an N-terminal SP is most certainly exported in Sec-dependent manner.

Besides, some lipoproteins (Table S3) and primarily cytoplasmic proteins were also predicted with extracellular localization following NC (Table S1). For example, it can be noted that some proteins lacking a SP can be secreted in a route involving the alternative cytosolic ATPase SecA2, a paralogue of SecA, that most certainly converge to the Sec translocon [4], [61].

### Comparison with SCL predictors applied to the extracytoproteome of *L. monocytogenes*

With 8 different predictable SCL in agreement with GO terms in Gram-positive bacteria, *i.e.* EM (GO:0005876), CS (GO:0009986), cell-surface protein complex (GO:0043234 AND 0009986), CW (GO:0009275), intrinsic to the CM (GO:0031226), integral to the CM (GO:0005887), anchored to the CM (GO:0046658), and cytoplasm (GO:0005737), other predictors never reach such a level of discrimination (Tables 3 and S7). Usually, 4 SCLs are considered as in CELLO [34], PSORTb [62] and Gpos-mPLoc [35] with the cytoplasm, membrane, cell wall and extracellular milieu. SubLoc [63] or LocTree [33] only differentiate cytoplasmic from extracellular location, and Augur the surface from the extracellular milieu [37]. Of note, results of some of these tools are not strictly in accordance with the GO and suffer of several misconceptions regarding the field of protein secretion [1], *e.g.* (i) SurfG+ [39] and LocateP [38] misleadingly refer to “secreted” for EM location, (ii) they mixes up SCLs with protein categories using descriptors such as LPXTG, multi-transmembrane or N-terminally anchored, which are further not in agreement with standart nomenclature for IMP for instance, (iii) SurfG+ considers “potentially surface exposed (PSE)” needs a certain amino acid length to cross the cell wall, whereas first, this is protein folding that would rather matter, and second, there is no need for a protein to poke out the confine of the cell wall to interact with their environment [60]. In addition and contrary to SubLoc, LocTree, Augur, PSORTb, SurfG+ or LocateP, the secretomics-based method allows attributing multiple SCL for a single protein.

**Table 3:** Performance evaluation metrics of the secretomics-based methods compared to other SCL predictors.

Tool	Actual location	GO^a^	Performance^b^				
				MCC	Accuracy	Sensitivity	Specificity
***Secretomics-based method***			Single				
	Extracellular milieu	GO:0005876		0.914	97.8	89.8	99.3
	Cell surface	GO:0009986		0.965	98.2	97.0	100.0
	Cell surface protein complex	GO:0043234 AND 0009986		1.000	100.0	100.0	100.0
	Cell wall	GO:0009275		1.000	100.0	100.0	100.0
	Intrinsic to the CM	GO:0031226		1.000	100.0	100.0	100.0
	Anchored to the CM	GO:0046658		1.000	100.0	100.0	100.0
	Integral to the CM	GO:0005887		1.000	100.0	100.0	100.0
	Cytoplasm	GO:0005737		1.000	100.0	100.0	100.0
			Overall	0.988	99.5	98.5	99.9
***SubLoc***			Single				
	Extracellular milieu	GO:0005876		0.267	70.0	65.3	70.8
	Cell surface	GO:0009986		n/a	n/a	n/a	n/a
	Cell surface protein complex	GO:0043234 AND 0009986		n/a	n/a	n/a	n/a
	Cell wall	GO:0009275		n/a	n/a	n/a	n/a
	Intrinsic to the CM	GO:0031226		n/a	n/a	n/a	n/a
	Anchored to the CM	GO:0046658		n/a	n/a	n/a	n/a
	Integral to the CM	GO:0005887		n/a	n/a	n/a	n/a
	Cytoplasm	GO:0005737		0.420	63.5	94.0	48.3
			Overall	0.396	66.7	85.5	60.8
***LocTree***			Single				
	Extracellular milieu	GO:0005876		0.298	68.5	73.5	67.7
	Cell surface	GO:0009986		n/a	n/a	n/a	n/a
	Cell surface protein complex	GO:0043234 AND 0009986		n/a	n/a	n/a	n/a
	Cell wall	GO:0009275		n/a	n/a	n/a	n/a
	Intrinsic to the CM	GO:0031226		n/a	n/a	n/a	n/a
	Anchored to the CM	GO:0046658		n/a	n/a	n/a	n/a
	Integral to the CM	GO:0005887		n/a	n/a	n/a	n/a
	Cytoplasm	GO:0005737		0.556	71.5	100.0	57.0
			Overall	0.471	70.0	92.0	63.0
***CELLO***			Single				
	Extracellular milieu	GO:0005876		0.030	73.8	20.4	82.8
	Cell surface	GO:0009986		n/a	n/a	n/a	n/a
	Cell surface protein complex	GO:0043234 AND 0009986		n/a	n/a	n/a	n/a
	Cell wall	GO:0009275		n/a	91.2	0.0	100.0
	Intrinsic to the CM	GO:0031226		0.572	78.8	77.7	79.7
	Anchored to the CM	GO:0046658		n/a	n/a	n/a	n/a
	Integral to the CM	GO:0005887		n/a	n/a	n/a	n/a
	Cytoplasm	GO:0005737		0.749	86.8	97.4	81.2
			Overall	0.553	82.6	69.5	87.1
***Gpos-mPloc***			Single				
	Extracellular milieu	GO:0005876		0.248	73.5	55.1	76.6
	Cell surface	GO:0009986		n/a	n/a	n/a	n/a
	Cell surface protein complex	GO:0043234 AND 0009986		n/a	n/a	n/a	n/a
	Cell wall	GO:0009275		0.230	89.3	23.3	95.8
	Intrinsic to the CM	GO:0031226		0.627	81.8	73.6	88.0
	Anchored to the CM	GO:0046658		n/a	n/a	n/a	n/a
	Integral to the CM	GO:0005887		n/a	n/a	n/a	n/a
	Cytoplasm	GO:0005737		0.745	87.7	91.5	85.9
			Overall	0.571	83.1	72.7	86.6
***Augur***			Single				
	Extracellular milieu	GO:0005876		0.310	85.0	32.7	93.8
	Cell surface	GO:0009986		0.691	83.5	76.4	93.6
	Cell surface protein complex	GO:0043234 AND 0009986		n/a	n/a	n/a	n/a
	Cell wall	GO:0009275		n/a	n/a	n/a	n/a
	Intrinsic to the CM	GO:0031226		n/a	n/a	n/a	n/a
	Anchored to the CM	GO:0046658		n/a	n/a	n/a	n/a
	Integral to the CM	GO:0005887		n/a	n/a	n/a	n/a
	Cytoplasm	GO:0005737		n/a	n/a	n/a	n/a
			Overall	0.654	84.3	67.7	93.8
***PSORTb***			Single				
	Extracellular milieu	GO:0005876		0.280	86.9	20.4	97.4
	Cell surface	GO:0009986		n/a	n/a	n/a	n/a
	Cell surface protein complex	GO:0043234 AND 0009986		n/a	n/a	n/a	n/a
	Cell wall	GO:0009275		0.730	95.6	76.7	97.4
	Intrinsic to the CM	GO:0031226		0.680	84.1	72.3	93.2
	Anchored to the CM	GO:0046658		n/a	n/a	n/a	n/a
	Integral to the CM	GO:0005887		n/a	n/a	n/a	n/a
	Cytoplasm	GO:0005737		0.831	92.4	88.9	94.2
			Overall	0.714	89.7	70.9	95.9
***SurfG+***			Single				
	Extracellular milieu	GO:0005876		0.378	87.5	34.7	95.8
	Cell surface	GO:0009986		0.470	70.4	54.8	90.4
	Cell surface protein complex	GO:0043234 AND 0009986		n/a	n/a	n/a	n/a
	Cell wall	GO:0009275		0.888	98.3	86.7	99.4
	Intrinsic to the CM	GO:0031226		0.803	90.3	90.5	90.1
	Anchored to the CM	GO:0046658		0.802	95.7	91.9	96.2
	Integral to the CM	GO:0005887		0.828	92.4	86.8	95.2
	Cytoplasm	GO:0005737		0.819	90.6	100.0	85.9
			Overall	0.730	89.3	77.2	94.0
***LocateP***			Single				
	Extracellular milieu	GO:0005876		0.392	88.6	26.5	98.4
	Cell surface	GO:0009986		n/a	n/a	n/a	n/a
	Cell surface protein complex	GO:0043234 AND 0009986		n/a	n/a	n/a	n/a
	Cell wall	GO:0009275		0.866	97.9	76.7	100.0
	Intrinsic to the CM	GO:0031226		0.789	89.2	94.6	85.2
	Anchored to the CM	GO:0046658		0.825	96.0	97.3	95.9
	Integral to the CM	GO:0005887		0.807	91.0	93.0	90.0
	Cytoplasm	GO:0005737		0.814	90.3	100.0	85.5
			Overall	0.790	92.1	87.9	93.4

a Subcellular location follow the GO (Gene Ontology) for cellular component.

b Performance was evaluated for single and overall SCL predictions for each tools. MCC (Matthews Correlation Coefficient) and other statistical metrics were calculated as described in the Materials & Methods section. Sensitivity, specificity and accuracy are expressed in %. Detailed performance evaluation metrics are provided in Table S7.

A major and original advance from the secretomics-based analysis resides in the clear and systematic differentiation of the protein category from the protein SCL. To objectively evaluate the performance to predict the protein SCL, the secretomics-based method and other SCL predictors were tested against a dataset of 337 distinct *L. monocytogenes* proteins, which actual location is defined and referenced (Table S7). SubLoc and LocTree exhibit the lowest overall performance as indicated by MCC values lower than 0.5 (**Table 3**). While they both show quite similar percentages of overall and single accuracy for SCL prediction, they exhibit an imbalance in their specificity with regard to the sensivity for protein prediction into the cytoplasm. With quite similar percentages of overall and single accuracy for SCL prediction, CELLO and Gpos-mPloc have MCC values just above 0.5. They both present an imbalance in their sensivity with regard to the specificity for SCL prediction to the EM and CW, especially CELLO, which appears inappropriate for EM and CW prediction as its sensitivity is null in the latter case.

With correct overall performance, Augur and PSORTb also present an imbalance in their sensivity/specificity for SCL prediction to the EM (**Table 3**). While the Augur database identifies the so-called “cell anchor” responsible for cell surface location [37], it suffers from numerous inaccuracies. The presence of LRR and NLPC/P60 domains are considered as cell anchors, which are not. Other well-known cell-wall binding domains, namely PGBD, CWBD, SLHD or WXL domain, are not taken into account. The absence of SP when a “cell anchor” is present does not underdetermine the surface prediction. From there, considering a “single secretome analysis” is performed, when just identifying all secreted proteins with SP and without “cell anchor”, is misleading. In the end, the proportion of genome encoded *L. monocytogenes* proteins, which SCL prediction is in agreement with the secretomics-based method is quite low, *i.e.* 67% (Table S7); with a number of Sec-secreted exoproteins estimated at 112, only 49% are in agreement with our present investigation as the remaining proteins exhibit cell-envelope retention domains. Conscious of the ambiguities in the Gram nomenclature especially in the field of protein secretion [1], [2], PSORTb proposes a confusing description to differentiate the analysis for bacteria with Gram positive or negative staining and those “positive with outer membrane” and “negative without outer membrane”. In line with a phylum level perspective on bacterial cell envelope architecture, however, the terminology of monoderm and diderm bacteria, which can even be discriminated between diderm-LPS and diderm-mycolate bacteria, is much more appropriate especially in the field of secretomics [1], [2], [64].

Among tested SCL predictors, SurfG+ and LocateP have the highest overall performances (**Table 3**). At a genome scale level, the agreement in SCL predictions of *L. monocytogenes* proteins between these tools and the secretomics-based approach is quite high, *i.e.* 95% and 92% respectively (Table S7). Back to the performance evaluation metrics, however, LocateP present an imbalance in its sensivity with regard to the specificity for SCL prediction to the EM, as well as to the CW for SurfG+ (**Table 3**). While the attempt by LocateP to differentiate the IMPs between multi-transmembrane, C-terminally and N-terminally anchored proteins with or without cleavable SP can be acknowledged, it also results in misprediction to the cytoplasm for several ssIMP II or III where the TMD is not located in the N-terminal or C-terminal region and then do not stand within the classification above (Table S7). Similarly in SurfG+ the differentiation of IMP topology into Loop-out, Nterm-out or Cterm-out tails does not compile with the standard nomenclature for IMP. Moreover, LocateP only considers LPXTG motif for SCL to the cell wall; as a result several proteins predicted as IMPs or exoproteins in *L. monocytogenes* EGD-e are in fact CW-proteins with LysM, GW, PGBD or WXL domains. Finally, the possibility for a single protein to belong to different protein categories is not fully considered by either of these two tools.

Compared to all other SCL predictors, the secretomics-based methods clearly outperform them both considering single and overall performances with accuracy reaching or close to 100% and MCC reaching or close to 1 for all of the 8 SCLs considered by the approach (**Table 3**). The secretomics-based method is the sole to take into consideration protein cell-surface protein complexes (GO:0043234 AND 0009986), *i.e.* S-layer (GO:0030115), cellulosome (GO:0043263), flagellum (GO:0019861), pseudo-pilus and pilus (GO:0009289). Actually, PSORTb considers flagellar components but it has not been especially designed to identify substrates to the FEA [62]; as a result, it could only find 3 out of the 11 components secreted by FEA in *L. monocytogenes* EGD-e and misleadingly predict them as located into the extracellular milieu (Table S7). Protein substrates of FPE or FEA are systematically mispredicted by other SCL predictors, most often as located in the cytoplasm or release in the extracellular milieu.

As the keystone of the secretomics-based method, none of the SCL predictors consider the secretion pathways, including protein routing, transport mechanisms and post-translocational modifications. In fact, LocateP was the best attempt to mimic secretion process but it is restricted to only 3 protein secretion systems, Sec, Tat and ABC protein exporter, plus NC secretion. Another major difference is that the pipeline misses some treads and does not follow the sequential steps of the biology of protein secretion, *e.g.* TMD prediction is considered before SP, processing of Type 4 prepilin is ruled out, or non-covalent anchoring of proteins to the cell wall is overlooked.

### Comparison with experimental proteomic analyses of the extracytoproteome of *L. monocytogenes*

Globally, the SCL from the secretomics-based approach is in good agreement with experimental data as revealed from the analyses of different extracytoplasmic fractions in *L. monocytogenes* EGD-e (Table S8). Information on protein trafficking has been experimentally confirmed for only a few proteins and concerns the (i) secretion of proteins dependent on the SPases I (SipX, SipY or SipZ) or Spase II LspA, (ii) SecA2-dependent protein secretion, (iii) protein anchoring to the CM by the prolipoprotein diacylglyceryltransferase Lgt, (iv) non-covalent anchoring to cell wall *via* GW repeats, or (v) covalent anchoring of proteins to the cell wall *via* sortases, *i.e.* SrtA and SrtB. Protein secretion system in used was demonstrated for none of them.

Concerning exoproteins, 26 were experimentally identified, mostly in culture supernatant but also in multi-fractions for some of them, namely membrane, cell wall and/or cell surface exposed fractions (Table S8). SP cleavage site was confirmed for only 3 of them, the involvement of SPase I SipZ for 2 of them, and the SecA2-dependent secretion for one of them. Most exoproteins experimentally identified are predicted secreted *via* Sec and two of them would be substrates of holin or ABC exporter, respectively.

Among CW-proteins, 35 were experimentally identified, essentially in the cell wall but some of them were also found in the membrane and/or supernatant fractions (Table S8). While the presence in the supernatant of CW-proteins covalently attached to the cell wall can be more surprising than those linked by weak interactions, it must considered that LPXTG-protein SCL is strictly associated with cell-wall biogenesis. Cell wall anchoring *via* sortases, SrtA or SrtB, was confirmed for 15 LPXTG-proteins but SP cleavage site as well as the involvement of SPase I SipZ and/or SipX was confirmed for only 2 CW-proteins, and the SecA2-dependent secretion for one of them.

With 117 IMP experimentally identified and categorized into ssIMP I, ssIMP II, ssIMP III and msIMP (Table S8), 36 lipoproteins essentially originate from membrane fraction (GO:005624). Again, some of them were also found in the cell wall, supernatant and/or cell surface exposed fractions. For IMP, the involvement of SPase I was confirmed for only one of them as well as SecA2-dependent translocation. Interestingly, most lipoproteins identified in the supernatant exhibited a G at C+2. SecA2-dependent secretion was demonstrated for 4 of them and the involvement of SPase II LspA for only 2 of them. SP cleavage site was confirmed for none. Anchoring to the CM by Lgt was actually confirmed for 26 lipoproteins.

With 192 representatives, the cytoproteins represent the largest category of experimentally identified proteins in extracytoplamic fractions of *L. monocytogenes* (Table S8). While only 6 of them could be predicted as secreted *via* NC in the first place, SecA2-dependent translocation was demonstrated for 12 of them. This category stresses the importance to discriminate the GO terms for SCL from the GO terms for the different cell fractions. For example, the presence of ribosomal proteins in membrane fractions is certainly related to SRP (Signal Recognition Particule)-dependent pathway, where ribosomes interact closely with the Sec translocon in the course of co-translational translocation. The SCL of such proteins would then be extrinsic to the CM (GO:0019897) and more precisely on the internal side (GO:0031234), still they would be identified in the membrane fraction (GO:0005624) and even more precisely peripheral to membrane of membrane fraction (GO:0000300) depending on the experimental protocol applied. Besides being secreted in a SecA2-dependent manner, the primary glycolytic enzyme enolase was demonstrated to moonlight when located extracytoplasmically since, together with DnaK, EF-TU and GAPDH, it binds to human plasminogen [44].

## Discussion

Compared with other SCL predictors for Gram-positive bacteria available to date, the secretomics-based method clearly outperforms them in term of single and overall performances. Accordingly, (i) protein routing is described and detailed in terms of secretion system, post-translocational modification, cell-envelope retention and/or incorporation to cell-surface supramolecular structures, (ii) the number of distinct SCL, as well as the different protein categories is the most comprehensive for monoderm bacterial cell, (iii) detailed description of protein type and topology is provided, (iv) it unambiguously differentiates the SCL from the protein category, and (v) the possibility for a single protein to belong to different protein categories as well as to have multiple SCL is fully considered. In that sense, the secretomics-based method is much more than a SCL predictor. In *L. monocytogenes*, this approach conveyed a highly significant quantity of new information, *e.g.* i) by considering for the first time ABC transporters in protein secretion, ii) presenting the first ever comprehensive estimate of IMPs with careful concern about their topology (msIMP, ssIMP I, II, or III) and double affiliation (to lipoprotein for instance), iii) reporting cell-surface supramolecular structures (flagellum and pseudo-pilus), iv) correcting the prediction of the number of lipoproteins or v) of non-covalently cell-wall anchored proteins by including PGBD1 and excluding proteins exhibiting only one GW domain. While protein attachment to the lipoteichoic acids of the cell wall is clearly modulated by the number of GW modules, i.e. complete release with one GW module and complete retention with 8 GW modules [57], [58], the proportion of retention/release from the bacterial cell for proteins with an in-between number of GW modules remain to be established. As a boundary, this combinatorial approach is based on the biology of protein secretion, which means it relies on the veracity of the knowledge in the field at a given time. This is also the strength of the secretomics-based method as revealed by its performance and its ability to be readily adjusted in light of new findings in the field.

As the keystone of the method, the protein secretion pathways in Gram-positive bacteria are fully taken into consideration and provided for each secreted proteins. Such an in-depth integration of the secretome concept for genomic analysis had never been achieved before. LocateP is the only SCL predictor attempting to reconsider protein secretion systems in monoderm bacteria but it misses some points, *e.g.* non-covalent cell-wall anchoring of secreted proteins, cell-surface supramolecular structures, or alternative secretion systems to Sec, Tat and ABC pathways [38]. The recently developed CoBaltDB, which combines at once prediction outputs from bioinformatic tools related to protein SCL is a valuable tool to get data prior to the secretomics-based analysis following the decision tree here described for monoderm bacteria [65]. While the basic limit of these tools resides in their lack of flexibility as new predictions tools, new basic findings in the field of protein secretion or genomes are made available, the secretomics-based approach can on the contrary be readily implemented within an Excel data table (Table S1). Nonetheless, we are currently working to make also available in the near future an online tool to ease and fasten the use of the secretomics-based method. Though, as for all pipelines combining results of individual prediction tools and developed to date, the secretomics-based method is based on majority-voting scheme for predictions; for example, LocateP applies unique, consensus and/or unanimously scheme at different stages of the decision trees [38]. Taking lipoprotein prediction tools for instance, they display different overall performances but also distinct precision and recall values [66], which should even be further considered in their single performances for the cleavage site prediction for instance. An awaited development in the field is the attempt to benchmark each individual tool for the subsequent weightings of each prediction.

Besides genomic analysis, this rational strategy is also useful for categorizing, analyzing, and supporting proteomic data. As an example, the prediction of a lipoprotein even by only one tool can be of high relevance if it backups some experimental proteomic data. An interesting aspect is the possibility to generate a database containing mature protein sequences, where any cleavable parts of pre(pro)proteins are removed [43]. The root of proteomics is indeed the protein identification based on expected peptide sizes deduced from *in silico* trypsinization of amino acid sequence. Confrontation of experimental data with the mature database can then allow experimental determination of the cleavage site in SP or sorting signal [43].

The secretomics-based method allows rigorous interpretation of experimental data by clearly differentiating the predicted SCL GO terms from the experimental cell fraction GO terms. While GO terms are available for proteins identified in membrane fractions (GO:0005624, 0000299, 0000300), they are still awaited for cell wall, surface exposed and supernatant fractions. This further pinpointed some limitations for SCL prediction. Despite very recent improvement in SignalP for example [67], straight prediction in bacteria of uncleaved SP serving as a signal anchor are still unavailable and thus requires intricate analyses (**Figure 3**). Due to limited experimental data on the IWZ (inner wall zone) [68], for which no GO term is as yet available, prediction of IWZ protein is not reliable. No bioinformatic tools have been developed to predict secreted peripheral proteins (GO:0019897, 0010339). Similarly, no SCL prediction into the cytosol of a host cell for substrates to cytolysin-meditated translocation could be performed.

By considering all molecular mechanisms known to date, the present secretomics-based analysis constitutes the ultimate step in mimicking the protein secretion steps for prediction of the final SCL. Contrary to all other SCL predictors, it does not only supply a static snapshot of protein SCL but also offers the full picture of the secretion process dynamics. Such insight provides strong basis to generate a wealth of *in silico* hypotheses that further fuel experimental work, *e.g.* demonstrating the secretion of a protein *via* a specific secretion system, its post-translational modification by specific sorting pathway, or the biological significance of peripheral proteins. Hopefully, this analytical strategy should be inspirational for the development of rational secretomics-based approaches dedicated to diderm-LPS (Gram-negative) bacteria. As a leitmotif in the field of bacterial protein secretion, the reason for the presence of primarily cytoproteins in the supernatant is an important issue. Besides autolysis, several alternative hypotheses can be formulated (i) allolysis, (ii) uncovered secretion systems, (iii) piggybacking, or (iv) leakage [69]. As exemplified with *L. monocytogenes*, the secretomics-based method provides the most updated and comprehensive genomic analysis of the extracytoproteome in a monoderm bacterium and should promote further experimental testing based on generated *in silico* hypotheses.

## Materials and Methods

Bioinformatic analyses were performed from web-based servers or under Linux environment with Sun Grid Engine (SGE) server hosted at INRA MIGALE Bioinformatics Platform from (INRA, Jouy-en-Josas, France) with a Intel Quad Core W3520 2.67 GHz. The cluster is organized around 4 computing groups, (i) AMD Quad Core 8354 2.8 GHz, (ii) Intel Quad Core E5520 2.27 GHz, (iii) Intel Quad Core 5340 2.33 GHz, and (iv) Intel Dual Core 5140 2.33 GHz. The complete genome, coding sequences (CDS) and annotation files for *L. monocytogenes* EGD-e were downloaded from GenBank (ftp://ftp.ncbi.nih.gov/genbank/genomes/Bacteria/Listeria_monocytogenes/).

Searches against various databases were performed using different tools, namely RPS-BLAST v2.2.19 (Reverse Position-Specific BLAST) [70], HMMER v2.3.2 for hidden Markov models (HMM) [71], InterProScan v4.3 [72], or ScanProsite v1.0 [73]. Interrogated databases included InterPro (IPR) v32.0 [74], Pfam (PF) v24.0 [75], SMART (SM) v6.1 [76], TIGRfam (TIGR) v10.1 [77], SuperFamily (SSF) SCOP v1.73 [78], [79], PIRSF v2.74 [80], PRK v3.0 [81], COG v1.0 [82] and Prosite (PS) v20.7 [83]. Position-specific iterated BLAST (PSI-BLAST v2.2.25) [70] searches were executed against UniProtKB v2011_07 [84] until convergence with unlimited number of database sequences for matrix building.

### Identification of N-terminal signal peptide

N-terminal signal peptide (SP) were predicted combining results from (i) SignalP v2.0 and v3.0 using both neural network (NN) and hidden Markov model (HMM) [85] with truncation set either at 35, 70, 140 or disabled and predictions with best scores and propensity were considered, (ii) PrediSi v1.0 [86], (iii) Phobius v1.0 [87], (iv) SOSUIsignal v1.0 [88], (v) Signal-3L v1.0 [89], (vi) SIG-PRED v1.0, (vii) Signal-CF v1.0 [90], (vii) SPScan v1.0, an implementation of von Heijne's weight matrix approach with McGeoch criteria where prompted parameters and optional parameter -ADJustscores were used and predictions with best scores and propensity were considered [91], [92], (viii) SPOctopus v1.0 [93], (ix) RPSP v1.0 [94], and (x) Signal-BLAST v1.0 [95]. Tat SP prediction was performed from (i) TatP v1.0 [96], (ii) TATFIND v1.4 [97], (iii) PRED-TAT v1.0 [98], and (iv) InterPro (IPR006311, IPR019546, PS51318, PF10518, TIGR01409). Whenever possible, all these previously cited tools were trained on prokaryotes, bacteria or Gram-positive bacteria. Pseudopilin-like SP were searched from PilFind v1.0 and with ScanProsite syntax [73] for consensus motif [AG]-F-[TSI]-[LTY]-x-[EAF] located between the N- and H-domains of the SP [4]. SP for protein substrates of ABC transporters were identified using BAGEL v1.0 [99]. Uncleaved signal peptides, *i.e.* N-terminal signal anchors, were predicted from SignalP using HMM trained on eukaryotes [85]. Identification of proteins with no signal peptide and secreted *via* alternative systems was based on genomic proximity with the genes encoding the respective secretion pathway as described below, coupled to similarity searches, namely substrates to holin and Wss (IPR018921, IPR010310, TIGR03930, PF10663, PF06013). Prediction of non-classical secreted proteins, *i.e*. lacking a signal peptide and translocated *via* unknown secretion system, was performed from (i) SecretomeP v2.0 [100], (2) SecretP v2.0 [101] and (3) NClassG+ v1.0 [102] trained on Gram-positive bacteria.

### Identification of protein secretion systems

Protein secretion systems were identification performing BLAST search against TC-DB v2011_07 [103], namely Sec (TC#3.A.5), Tat (TC#2.A.64), ABC (TC#3.A.1), FPE (TC#3.A.14), FEA (TC#3.A.6.2), holins (TC#1.E), and Wss (TC#9.A.44) pathways. To discriminate ABC transporters involved in protein secretion, results were compiled with BAGEL [99]. Results were refined by similarity searches, also to identify signal peptidases of Type I (SPase I, IPR000223, COG0681, PRK10861, TIGR02227, SSF51306), SPaseII (COG0597, PRK00376, PRK01574, IPR001872, TIGR00077, PF01252) and prepilin peptidase (COG1989, IPR000045, PF01478), the lipoprotein maturation pathway for some Sec- or Tat substrates, namely in addition to SPase II, the prolipoprotein diacylglyceryltransferase Lgt (COG0682, PRK00052, PRK12437, PRK13108, IPR001640, TIGR00544, PF01790) and the apolipoprotein N-acyltransferase (Lnt) (COG0815, IPR004563, TIGR00546) and the covalent cell-wall anchoring sortase (COG3764, IPR005754, IPR009835, IPR015986, IPR23365, IPR022445, TIGR01076, TIGR03064, TIGR03784, SSF63817, PF04203, PF07170, PIRSF029877, PIRSF030150) pathway for some Sec-substrates.

### Identification of transmembrane hydrophobic domains and lipoproteins

Transmembrane hydrophobic α-helice domain (TMD) were predicted combining (i) TMHMM v2.0 [30], (ii) SVMtm v1.0 [104] (iii) THUMBUP v1.0 [105], (iv) SOSUI v1.10 [106], (v) HMMTOP v2.0 [107], (vi) PHDhtm v8.94_69 [108], (vii) UMDHMMTMHP v1.0 [105], (vii) MEMSAT v3.0 [109], (ix) MemBrain v1.0 [110], (x) OCTOPUS v1.0 [111], (xi) MINNOU v1.0 [112], (xii) Philius v1.0 [113], (xiii) SCAMPI v1.0 [114], (xiv) TMPred v1.0 [115], (xv) SPLIT v4.0 [116], and (xvi) TOPCONS v1.0 [117].

For the identification of lipobox, sequences were submitted to (i) DOLOP v2.0 [118], (ii) LipoP v1.0 [29], (iii) SPEPLip v1.0 [119], (iv) LipPred v1.0 [120], (v) ScanProsite for a scan with both PS51257 profile and G+LPP v2.0 pattern [121], (vi) LIPO [122], and (vii) PRED-LIPO [123].

### Identification of cell-wall attachment domains

Besides similarity search from InterPro (IPR001899, IPR017502, IPR019931, IPR017503, IPR019948, TIGR01167, TIGR03063, TIGR03065, PF00746, PS50847), LPXTG domain were specifically identified by LPXTG-HMM profile [54] and CW-PRED v2.0 [31]. The concomitant presence of YSIRK domain (IPR005877, PF04650, TIGR01168) within Sec-SP was also checked [55], [56]. WXL domain were found scanning for the motif [LI]-[TE]-W-[TS]-L with ScanProsite where C-terminal location of the motif was also taken into account [124]. Similarity searches were performed for other cell-wall binding motifs, namely (i) LysM (IPR018392, IPR002482, PF01476, SM00257, SSF54106), (ii) GW (SSF82057), (iii) choline-binding domain, also called cell-wall binding domain of type 1 (CWBD1; IPR018337, PF01473, PS51170, SSF69360), (iv) CWBD2 (IPR007253, PF04122), (v) peptidoglycan-binding domain of type 1 (PGBD1; IPR002477, PF01471, SSF47090), (vi) PGBD2 (IPR014927, PF08823), (vii) PGBD3 (IPR018537, PF09374, SSF53955), PGBD4 (IPR022029, PF12229, SSF143985), and S-layer homology domain (SLHD; IPR001119, PF00395, PS51272).

### Identification of cell-surface supramolecular structure

Besides the identification of pseudo-pilus components based on the identification of proteins with pseudopilin-like signal peptides as described above, pilus in Gram-positive bacteria was identified from LOCP [125]. Identification of proteins substrates to FEA followed similarity search (IPR001029, IPR00149, IPR010809, IPR003481, IPR013384, IPR002371, IPR020013, IPR005648, IPR021136, IPR001444, IPR006300, IPR006299, IPR001624, COG1345, COG1334, COG1344, COG1256, COG1749, COG1843, COG4786, COG4787, COG1815, COG1558, COG1677, SSF64518, SSF117143), as well as proteins substrates to Wss (IPR018921, IPR010310, TIGR03930, PF10663, PF06013). Identification of cellulosome components was based on dockerin/cohesin domains (IPR002102, IPR018452, IPR016134, IPR002105, IPR018242, IPR009034, IPR002883) and for S-layer on SLHD (IPR001119, COG1361) coupled to PSI-BLAST search.

### The secretomics-based method

The method is truly based on the biology of protein secretion in monoderm bacterium (**Figure 1**). The different types of N-terminal SP targeting a protein to the membrane are discriminated into SP specific for Sec, Tat, ABC and FPE, as well as uncleaved SP (Unc-SP). The protein secretion systems considered are Sec, Tat, ABC, holins, Wss, FPE, FEA, and non-classical secretion (NC). Both transmembrane domain (TMD), including uncleaved SP, or post-translational modifications, *i.e.* lipobox, are considered for membrane retention. Domains (PGBD, CWBD, WXL, LysM, SLHD, GW) or post-translational modification (LPXTG) are considered for cell-wall retention. Besides flagellum and pseudopilus secreted *via* the FPE and FEA, S-layer, pilus and cellulosome are also considered as cell-surface supramolecular structures.

The coding sequences (CDS) are screened simultaneously following a flowchart (**Figure 2**), which provides results from the prediction tools described above and organizes to define the different databases as exemplified in Table S1, namely (i) signal peptide (SP), (ii) type of SP, (iv) protein secretion systems, (v) export (proteins lacking a SP), (iv) transmembrane domain (TMD), and (iv) relevant conserved motifs. Results of different tools for similar prediction as combined into a majority vote approach, *i.e.* SP, FPE-SP, Tat-SP, ABC-SP, Lipobox, Export and TMD. Using specific tools, proteins exhibiting SP specific for Tat, FPE and ABC transporters are defined as well as proteins with signal anchor, *i.e.* uncleaved SP, or lipobox in SP of Type II (SP II); combined with the 14 distinct predictions resulting from 11 tools, proteins exhibiting a N-terminal SP are delineated after checking the compatibility with functional annotation verified by similarity search with InterProScan and/or PSI-BLAST. Besides secretion systems, substrates of FEA, holin and Wss are identified following BLAST search against TC-DB. Exported proteins are identified using specific tools for NC. Prediction of proteins with TMD combined 16 tools, 12 of them provide further information on protein topology. Topological information allows to categorised proteins into msIMP, ssIMP I, ssIMP II or ssIMP III. Proteins with a lipobox in SP II are extracted from 8 distinct predictions. Finally, identification of conserved motifs for cell wall anchoring or surface appendages results from interrogation of 10 distinct databases. Parietal proteins are discriminated between LPXTG-, WXL-, SLHD-, LysM-, GW-, CWBD1/2- and/or PGBD1/2/3/4-protein.

From there and as a key step of the the secretomics-based method, the resulting databases are analysed as depicted in the detailed decision trees (**Figure 3**). As a result of the decision trees, proteins are discriminated into 10 distinct primary categories; IMPs can be further discriminated into multi-spanning IMP (msIMP), single-spanning IMP of Type I (ssIMP I), II (ssIMP II) or III (ssIMP III), whereas parietal protein (CW-protein) can be dicrimnated into 12 subtypes (LPXTG-protein, LysM protein, CWBD1-protein,...*etc*). In agreement with GO terms, the secretomics-based method provides 4 primary SCL, CP (GO:0005737), CM (GO:0005886), CW (GO:0009275) and EM (GO:0005576); protein complex (GO:0043234), cell surface (GO:0009986), integral (GO:0005887) and anchored to CM (GO:0046658) SCL are further discriminated.

### Subcellular localization predictors

Predictions for SCL were performed from pipelines, *i.e.* Augur v2010_07 [37], LocateP v1.0 [38] and SurfG+ [39], as well as support vector machines (SVMs), *i.e.* SubLoc v1.0 [63] and LocTree v1.0 [33] trained on prokaryotes where only extracellular and cytoplasmic SCL are considered, whereas CELLO v2.5 [34], PSORTb v3.0.2 [62] and Gpos-mPLoc v1.0 [35] trained on Gram-positive bacteria considered extracellular, cell wall, membrane and cytoplasmic subcellular compartments as well as multiple localization sites.

### Performance evaluation metrics

Sensitivity, specificity, accuracy and MCC (Matthews Correlation Coefficient) of the SCL methods were calculated from the four basic statistics, *i.e.* true positives (TP), false negatives (FN), false positives (FP) and true negatives (TN) [126]. Sensitivity was calculated as TP/(TP+FN), specificity as TN/(TN+FP), accuracy as (TP+TN)/(TP+FN+FP+TN), and MCC as [(TP×TN)-(FN×FP)]/√[(TP+FN)×(TP+FP)×(TN+FP)×(TN+FN)]. Sensitivity, specificity and accuracy were expressed as percentage, whereas MMC values can vary between −1 and 1. Taking the extracellular milieu (EM) as an example of SCL prediction, (i) TP corresponds to instances both predicted and actually located in the EM, (ii) FN to instances actually located in the EM but not predicted in the EM, (iii) FP to instances predicted in the EM but actually not located in the EM, and (iv) TN to instances both predicted and actually not located in the EM. Besides the overall performance of a given tool, single performances were evaluated for each of the different SCL considered. A dataset was constructed from *L. monocytogenes* proteins, which location was experimentally confirmed and/or acknowledged from literature survey (Table S7). It consisted of 337 distinct proteins localized in the (i) extracellular milieu (EM; 49 proteins), (ii) cell surface (CS; 199 proteins), (iii) protein complex (PC; 16 proteins), (iv) cell wall (CW; 30 proteins), (v) intrinsic to the cytoplasmic membrane (CM; 148 proteins), (vi) anchored to the CM (aCM; 37 proteins), (vii) integral to the CM (iCM; 114 proteins), and/or (viii) cytoplasm (CP; 117 proteins) (Table S7).

## Supporting Information

Table S1 **Complete results from secretomics-based genomic analysis allowing prediction of protein types and SCL in** ***L. monocytogenes*** **EGD-e.**
(XLSX)

Table S2 **Summarised information about protein categories, secretion pathways and GO terms for IMPs, lipoproteins, cell-wall proteins, subunits of supramolecular cell-surface appendages and exoproteins, respectively, as predicted by the secretomics-based method in** ***L. monocytogenes*** **EGD-e.**
(PDF)

Table S3 **Summarised information about protein categories, secretion pathways and GO terms for IMPs, lipoproteins, cell-wall proteins, subunits of supramolecular cell-surface appendages and exoproteins, respectively, as predicted by the secretomics-based method in** ***L. monocytogenes*** **EGD-e.**
(PDF)

Table S4 **Summarised information about protein categories, secretion pathways and GO terms for IMPs, lipoproteins, cell-wall proteins, subunits of supramolecular cell-surface appendages and exoproteins, respectively, as predicted by the secretomics-based method in** ***L. monocytogenes*** **EGD-e.**
(PDF)

Table S5 **Summarised information about protein categories, secretion pathways and GO terms for IMPs, lipoproteins, cell-wall proteins, subunits of supramolecular cell-surface appendages and exoproteins, respectively, as predicted by the secretomics-based method in** ***L. monocytogenes*** **EGD-e.**
(PDF)

Table S6 **Summarised information about protein categories, secretion pathways and GO terms for IMPs, lipoproteins, cell-wall proteins, subunits of supramolecular cell-surface appendages and exoproteins, respectively, as predicted by the secretomics-based method in** ***L. monocytogenes*** **EGD-e.**
(PDF)

Table S7 **Detailed performance evaluation metrics and comparison of prediction results in** ***L. monocytogenes*** **EGD-e between secretomics-based method and available SCL predictors.**
(XLSX)

Table S8 **Secretomic analysis of extracytoplasmic proteins experimentally identified in** ***L. monocytogenes*** **EGD-e.**
(PDF)
