# Supplementary material for: Subcellular Localization of Extracytoplasmic Proteins in Monoderm Bacteria: Rational Secretomics-Based Strategy for Genomic and Proteomic Analyses
Source: PLoS One. 2012 Aug 9;7(8):e42982. doi: 10.1371/journal.pone.0042982 (PMC3415414; doi:10.1371/journal.pone.0042982)
Supplement: Table S2 — Summarised information about protein categories, secretion pathways and GO terms for IMPs, lipoproteins, cell-wall proteins, subunits of supramolecular cell-surface appendages and exoproteins, respectively, as predicted by the secretomics-based method in L. monocytogenes EGD-e. (PDF) [file pone.0042982.s002.pdf]

Table 2S: **Integral membrane proteins (IMPs) in *L. monocytogenes* EGD-e as revealed by the secretomics-based method.**

Protein ID	Annotation[a]	Protein Type[b]	Secretion pathway	GO[c]
Single-spanning integral membrane proteins				
Lmo0058	WXG100 protein secretion system, membrane component, EssA	ssIMP I	Sec, YidC, SPase I	0031226, 0005887, 0009986
Lmo0082	Protein of unknown function	ssIMP I	Sec, YidC, SPase I	0031226, 0005887, 0009986
Lmo0204	Actin-assembly inducing protein, ActA	ssIMP I	Sec, YidC, SPase I	0031226, 0005887, 0009986
Lmo0528	Protein of unknown function, COG5298	ssIMP I	Sec, YidC, SPase I	0031226, 0005887, 0009986
Lmo0530	Protein of unknown function	ssIMP I	Sec, YidC, SPase I	0031226, 0005887, 0009986
Lmo0552	Protein of unknown function, DUF916 domain, CscA-like	ssIMP I	Sec, YidC, SPase I	0031226, 0005887, 0009986
Lmo0586	Protein of unknown function, DUF916 domain, CscA-like	ssIMP I	Sec, YidC, SPase I	0031226, 0005887, 0009986
Lmo0701	Protein of unknown function	ssIMP I	Sec, YidC, SPase I	0031226, 0005887, 0009986
Lmo1021	Signal transduction histidine kinase, LiaS	ssIMP I	Sec, YidC, SPase I	0031226, 0005887, 0009986
Lmo1205	Cobalt transport protein, CbiN	ssIMP I	Sec, YidC, SPase I	0031226, 0005887, 0009986
Lmo1378	Signal transduction histidine kinase, LisK	ssIMP I	Sec, YidC, SPase I	0031226, 0005887, 0009986
Lmo1461	Protein of unknown function	ssIMP I	Sec, YidC, SPase I	0031226, 0005887, 0009986
Lmo1610	Protein of unknown function	ssIMP I	Sec, YidC, SPase I	0031226, 0005887, 0009986
Lmo2061	Protein of unknown function with GLE1 domain, COG4549	ssIMP I	Sec, YidC, SPase I	0031226, 0005887, 0009986
Lmo2410	Membrane protein of unknown function	ssIMP I	Sec, YidC, SPase I	0031226, 0005887, 0009986
Lmo2451	Sec translocon, subunit SecG	ssIMP I	Sec, YidC, SPase I	0031226, 0005887, 0009986
Lmo2534	F_0F_1 ATP synthase, subunit C	ssIMP I	Sec, YidC, SPase I	0031226, 0005887, 0009986
Lmo2706	Protein of unknown function	ssIMP I	Sec, YidC, SPase I	0031226, 0005887, 0009986
Lmo0821	Lipoprotein of unknown function	ssIMP I-Lipoprotein	Sec, YidC, Lgt, SPase II	0031226, 0005887, 0046658, 0009986
Lmo2793	Lipoprotein of unknown function	ssIMP I-Lipoprotein	Sec, YidC, Lgt, SPase II	0031226, 0005887, 0046658, 0009986
Lmo0060	WXG100 protein secretion system, membrane component, YukC (EssB)	ssIMP II	YidC	0031226, 0005887, 0009986
Lmo0066	Protein of unknown function, COG5444	ssIMP II	YidC	0031226, 0005887, 0009986
Lmo0292	Trypsin-like serine protease with C-terminal PDZ/DHR/GLGF domain	ssIMP II	YidC	0031226, 0005887, 0009986
Lmo0383	Methylmalonate-semialdehyde dehydrogenase	ssIMP II	YidC	0031226, 0005887, 0009986
Lmo0502	Sugar phosphate isomerase involved in capsule formation	ssIMP II	YidC	0031226, 0005887, 0009986
Lmo0702	Protein of unknown function	ssIMP II	YidC	0031226, 0005887, 0009986
Lmo0763	Phosphohydrolases	ssIMP II	YidC	0031226, 0005887, 0009986

Lmo0890	Sulphate transporter/antisigma-factor antagonist STAS	ssIMP II	YidC	0031226, 0005887, 0009986
Lmo0974	D-alanine--D-alanyl carrier protein ligase	ssIMP II	YidC	0031226, 0005887, 0009986
Lmo1075	Teichoic acids export protein ATP-binding subunit	ssIMP II	YidC	0031226, 0005887, 0009986
Lmo1097	Protein of unknown function	ssIMP II	YidC	0031226, 0005887, 0009986
Lmo1106	ATPase	ssIMP II	YidC	0031226, 0005887, 0009986
Lmo1194	Cobalamin (vitamin B12) biosynthesis CbiD	ssIMP II	YidC	0031226, 0005887, 0009986
Lmo1198	Cobalamin (vitamin B12) biosynthesis CbiG	ssIMP II	YidC	0031226, 0005887, 0009986
Lmo1374	Catalytic domain of dehydrogenase complex,biotin/lipoyl attachment	ssIMP II	YidC	0031226, 0005887, 0009986
Lmo1395	DNA-binding λ repressor-like, COG1426	ssIMP II	YidC	0031226, 0005887, 0009986
Lmo1414	Thiolase	ssIMP II	YidC	0031226, 0005887, 0009986
Lmo1433	Pyruvate/oxoglutarate dehydrogenase complex, dihydrolipoamide dehydrogenase	ssIMP II	YidC	0031226, 0005887, 0009986
Lmo1552	Valyl-tRNA synthetase, class Ia	ssIMP II	YidC	0031226, 0005887, 0009986
Lmo1663	Asparagine synthase, glutamine-hydrolyzing	ssIMP II	YidC	0031226, 0005887, 0009986
Lmo1820	Serine/threonine protein kinase	ssIMP II	YidC	0031226, 0005887, 0009986
Lmo1927	3-dehydroquinate synthase, AroB	ssIMP II	YidC	0031226, 0005887, 0009986
Lmo2202	β-ketoacyl-acyl carrier protein synthase III, FabH	ssIMP II	YidC	0031226, 0005887, 0009986
Lmo2390	Thioredoxin reductase	ssIMP II	YidC	0031226, 0005887, 0009986
Lmo2757	DNA helicase, ATP-dependent, RecQ type	ssIMP II	YidC	0031226, 0005887, 0009986
Lmo2769	ABC-type multidrug transport system	ssIMP II	YidC	0031226, 0005887, 0009986
Lmo2833	Maltose phosphorylase/glycosyl hydrolase/vacuolar acid trehalase	ssIMP II	YidC	0031226, 0005887, 0009986
Lmo0039	Carbamate kinase	ssIMP II, Unc-SP	Sec, YidC	0031226, 0005887, 0009986
Lmo0052	Signal transduction phosphoesterase YybT type with GDEF and DHH domains	ssIMP II, Unc-SP	Sec, YidC	0031226, 0005887, 0009986
Lmo0089	F_0F_1-type ATP synthase, δ subunit	ssIMP II, Unc-SP	Sec, YidC	0031226, 0005887, 0009986
Lmo0104	Protein of unknown function	ssIMP II, Unc-SP	Sec, YidC	0031226, 0005887, 0009986
Lmo0186	Protein of unknown function with 3D and G5 domains	ssIMP II, Unc-SP	Sec, YidC	0031226, 0005887, 0009986
Lmo0193	Macrolide transporter subunit MacA	ssIMP II, Unc-SP	Sec, YidC	0031226, 0005887, 0009986
Lmo0217	Septum formation initiator	ssIMP II, Unc-SP	Sec, YidC	0031226, 0005887, 0009986
Lmo0245	Sec translocon, subunit SecE	ssIMP II, Unc-SP	Sec, YidC	0031226, 0005887, 0009986
Lmo0289	Signal transduction YycH protein	ssIMP II, Unc-SP	Sec, YidC	0031226, 0005887, 0009986
Lmo0290	Protein of unknown function, COG4853	ssIMP II, Unc-SP	Sec, YidC	0031226, 0005887, 0009986
Lmo0299	Phosphotransferase system cellobiose-specific component IIB	ssIMP II, Unc-SP	Sec, YidC	0031226, 0005887, 0009986
Lmo0353	Acyl-CoA N-acyltransferase	ssIMP II, Unc-SP	Sec, YidC	0031226, 0005887, 0009986

Lmo0354	Acyl-coenzyme A synthetase/AMP-(fatty) acid ligase	ssIMP II, Unc-SP	Sec, YidC	0031226, 0005887, 0009986
Lmo0362	Twin-arginine translocase protein A, TatA	ssIMP II, Unc-SP	Sec, YidC	0031226, 0005887, 0009986
Lmo0376	Transcriptional regulator	ssIMP II, Unc-SP	Sec, YidC	0031226, 0005887, 0009986
Lmo0392	Protein of unknown function with DUF1432, COG4864	ssIMP II, Unc-SP	Sec, YidC	0031226, 0005887, 0009986
Lmo0393	Protein of unknown function	ssIMP II, Unc-SP	Sec, YidC	0031226, 0005887, 0009986
Lmo0399	Phosphotransferase system, fructose-specific IIB subunit	ssIMP II, Unc-SP	Sec, YidC	0031226, 0005887, 0009986
Lmo0427	Phosphotransferase system, fructose-specific IIB subunit	ssIMP II, Unc-SP	Sec, YidC	0031226, 0005887, 0009986
Lmo0441	Cell division protein FtsI/Penicillin-binding protein 2, transpeptidase	ssIMP II, Unc-SP	Sec, YidC	0031226, 0005887, 0009986
Lmo0442	Protein of unknown function	ssIMP II, Unc-SP	Sec, YidC	0031226, 0005887, 0009986
Lmo0443	Transcriptional regulator	ssIMP II, Unc-SP	Sec, YidC	0031226, 0005887, 0009986
Lmo0465	Protein of unknown function	ssIMP II, Unc-SP	Sec, YidC	0031226, 0005887, 0009986
Lmo0477	Protein of unknown function	ssIMP II, Unc-SP	Sec, YidC	0031226, 0005887, 0009986
Lmo0478	Protein of unknown function	ssIMP II, Unc-SP	Sec, YidC	0031226, 0005887, 0009986
Lmo0479	Protein of unknown function	ssIMP II, Unc-SP	Sec, YidC	0031226, 0005887, 0009986
Lmo0481	Streptococcal 67 kDa myosin-cross-reactive antigen like	ssIMP II, Unc-SP	Sec, YidC	0031226, 0005887, 0009986
Lmo0592	Protein of unknown function	ssIMP II, Unc-SP	Sec, YidC	0031226, 0005887, 0009986
Lmo0595	O-acetylhomoserine/O-acetylserine sulfhydrylase	ssIMP II, Unc-SP	Sec, YidC	0031226, 0005887, 0009986
Lmo0628	Protein of unknown function	ssIMP II, Unc-SP	Sec, YidC	0031226, 0005887, 0009986
Lmo0661	Carboxymuconolactone decarboxylase	ssIMP II, Unc-SP	Sec, YidC	0031226, 0005887, 0009986
Lmo0675	Protein of unknown function	ssIMP II, Unc-SP	Sec, YidC	0031226, 0005887, 0009986
Lmo0686	Flagellar motor protein MotB	ssIMP II, Unc-SP	Sec, YidC	0031226, 0005887, 0009986
Lmo0746	Protein of unknown function	ssIMP II, Unc-SP	Sec, YidC	0031226, 0005887, 0009986
Lmo0810	Spermidine/putrescine ABC transporter, substrate-binding protein	ssIMP II, Unc-SP	Sec, YidC	0031226, 0005887, 0009986
Lmo0914	Phosphotransferase system, lactose/cellobiose-specific IIB subunit	ssIMP II, Unc-SP	Sec, YidC	0031226, 0005887, 0009986
Lmo0926	Transcriptional regulator	ssIMP II, Unc-SP	Sec, YidC	0031226, 0005887, 0009986
Lmo0929	Sortase A, SrtA	ssIMP II, Unc-SP	Sec, YidC	0031226, 0005887, 0009986
Lmo0951	Protein of unknown function with α/β-hydrolase fold and DUF1801, COG4814	ssIMP II, Unc-SP	Sec, YidC	0031226, 0005887, 0009986
Lmo0962	Listeria epitope, LemA	ssIMP II, Unc-SP	Sec, YidC	0031226, 0005887, 0009986
Lmo0971	D-alanine esterification of (lipo-)teichoic acid protein, DltD	ssIMP II, Unc-SP	Sec, YidC	0031226, 0005887, 0009986
Lmo1026	Transcriptional regulator	ssIMP II, Unc-SP	Sec, YidC	0031226, 0005887, 0009986
Lmo1049	Thiamine/molybdopterin biosynthesis ThiF/MoeB-like protein	ssIMP II, Unc-SP	Sec, YidC	0031226, 0005887, 0009986
Lmo1095	Phosphotransferase system, lactose/cellobiose-specific IIB subunit	ssIMP II, Unc-SP	Sec, YidC	0031226, 0005887, 0009986

Lmo1103	Protein of unknown function	ssIMP II, Unc-SP	Sec, YidC	0031226, 0005887, 0009986
Lmo1128	Lysophospholipase	ssIMP II, Unc-SP	Sec, YidC	0031226, 0005887, 0009986
Lmo1215	Muramidase flagellum-specific with a single GW domain, FlgJ-type	ssIMP II, Unc-SP	Sec, YidC	0031226, 0005887, 0009986
Lmo1269	Signal peptidase of Type I, SipX	ssIMP II, Unc-SP	Sec, YidC	0031226, 0005887, 0009986
Lmo1270	Signal peptidase of Type I, SipY	ssIMP II, Unc-SP	Sec, YidC	0031226, 0005887, 0009986
Lmo1271	Signal peptidase of Type I, SipZ	ssIMP II, Unc-SP	Sec, YidC	0031226, 0005887, 0009986
Lmo1380	Protein of unknown function	ssIMP II, Unc-SP	Sec, YidC	0031226, 0005887, 0009986
Lmo1399	HD superfamily hydrolase	ssIMP II, Unc-SP	Sec, YidC	0031226, 0005887, 0009986
Lmo1484	DNA uptake competence protein, ComEA	ssIMP II, Unc-SP	Sec, YidC	0031226, 0005887, 0009986
Lmo1495	Protein of unknown function with DUF1510 domain	ssIMP II, Unc-SP	Sec, YidC	0031226, 0005887, 0009986
Lmo1499	Aminodeoxychorismate lyase	ssIMP II, Unc-SP	Sec, YidC	0031226, 0005887, 0009986
Lmo1511	Lysophospholipase	ssIMP II, Unc-SP	Sec, YidC	0031226, 0005887, 0009986
Lmo1529	Sec transcolon, subunit YajC	ssIMP II, Unc-SP	Sec, YidC	0031226, 0005887, 0009986
Lmo1547	Cell shape-determining protein, MreC	ssIMP II, Unc-SP	Sec, YidC	0031226, 0005887, 0009986
Lmo1715	S-adenosyl-L-methionine-dependent methyltransferase type 11	ssIMP II, Unc-SP	Sec, YidC	0031226, 0005887, 0009986
Lmo1720	Phosphotransferase system, lactose/cellobiose-specific IIB subunit	ssIMP II, Unc-SP	Sec, YidC	0031226, 0005887, 0009986
Lmo1813	Iron-sulphur-dependent L-serine dehydratase beta subunit	ssIMP II, Unc-SP	Sec, YidC	0031226, 0005887, 0009986
Lmo1861	Protein of unknown function, COG4698	ssIMP II, Unc-SP	Sec, YidC	0031226, 0005887, 0009986
Lmo1892	Membrane carboxypeptidase (penicillin-binding protein), PbpA	ssIMP II, Unc-SP	Sec, YidC	0031226, 0005887, 0009986
Lmo1898	Protein of unknown function, COG5353	ssIMP II, Unc-SP	Sec, YidC	0031226, 0005887, 0009986
Lmo1924	Prephenate dehydrogenase	ssIMP II, Unc-SP	Sec, YidC	0031226, 0005887, 0009986
Lmo2034	Cell division septal protein, FtsQ	ssIMP II, Unc-SP	Sec, YidC	0031226, 0005887, 0009986
Lmo2036	UDP-N-acetylmuramoylalanine-D-glutamate ligase, MurD	ssIMP II, Unc-SP	Sec, YidC	0031226, 0005887, 0009986
Lmo2040	Cell division protein, FtsL -like	ssIMP II, Unc-SP	Sec, YidC	0031226, 0005887, 0009986
Lmo2051	Protein of unknown function with PDZ domain	ssIMP II, Unc-SP	Sec, YidC	0031226, 0005887, 0009986
Lmo2056	Ca^{2+} chelating serine protease with SCP/PR1 domain	ssIMP II, Unc-SP	Sec, YidC	0031226, 0005887, 0009986
Lmo2089	Esterase/lipase	ssIMP II, Unc-SP	Sec, YidC	0031226, 0005887, 0009986
Lmo2181	Sortase B, SrtB	ssIMP II, Unc-SP	Sec, YidC	0031226, 0005887, 0009986
Lmo2229	Carboxypeptidase (penicillin-binding protein)	ssIMP II, Unc-SP	Sec, YidC	0031226, 0005887, 0009986
Lmo2257	Protein of unknown function	ssIMP II, Unc-SP	Sec, YidC	0031226, 0005887, 0009986
Lmo2258	Protein of unknown function	ssIMP II, Unc-SP	Sec, YidC	0031226, 0005887, 0009986
Lmo2280	Protein of unknown function, COG1422	ssIMP II, Unc-SP	Sec, YidC	0031226, 0005887, 0009986

Lmo2373	Phosphotransferase system, lactose/cellobiose-specific IIB subunit	ssIMP II, Unc-SP	Sec, YidC	0031226, 0005887, 0009986
Lmo2420	Protein of unknown function	ssIMP II, Unc-SP	Sec, YidC	0031226, 0005887, 0009986
Lmo2442	Protein of unknown function, DUF218 domain, COG1434	ssIMP II, Unc-SP	Sec, YidC	0031226, 0005887, 0009986
Lmo2485	Stress-responsive transcriptional regulator	ssIMP II, Unc-SP	Sec, YidC	0031226, 0005887, 0009986
Lmo2486	Protein of unknown function, UCP012569 type, COG3595	ssIMP II, Unc-SP	Sec, YidC	0031226, 0005887, 0009986
Lmo2518	Transcriptional regulator	ssIMP II, Unc-SP	Sec, YidC	0031226, 0005887, 0009986
Lmo2680	K+ transporting ATPase, C subunit, KdpC	ssIMP II, Unc-SP	Sec, YidC	0031226, 0005887, 0009986
Lmo2683	Phosphotransferase system, lactose/cellobiose-specific IIB subunit	ssIMP II, Unc-SP	Sec, YidC	0031226, 0005887, 0009986
Lmo2710	Protein of unknown function	ssIMP II, Unc-SP	Sec, YidC	0031226, 0005887, 0009986
Lmo2762	Phosphotransferase system, lactose/cellobiose-specific IIB subunit	ssIMP II, Unc-SP	Sec, YidC	0031226, 0005887, 0009986
Lmo2805	Protein of unknown function with DUF1310 domain	ssIMP II, Unc-SP	Sec, YidC	0031226, 0005887, 0009986
Lmo2806	Protein of unknown function with DUF1310 domain	ssIMP II, Unc-SP	Sec, YidC	0031226, 0005887, 0009986
Lmo2807	Protein of unknown function with DUF1310 domain	ssIMP II, Unc-SP	Sec, YidC	0031226, 0005887, 0009986
Lmo2808	Protein of unknown function with DUF1310 domain	ssIMP II, Unc-SP	Sec, YidC	0031226, 0005887, 0009986
Lmo2809	Protein of unknown function with DUF1310 domain	ssIMP II, Unc-SP	Sec, YidC	0031226, 0005887, 0009986
Lmo1303	Cell division suppressor protein, YneA, with LysM domain	ssIMP II-LysM-protein, Unc-SP	Sec, YidC	0031226, 0005887, 0009275, 0009986
Lmo1941	Protein of unknown function, YpbE-like, LysM and ATPase domains	ssIMP II-LysM-protein, Unc-SP	Sec, YidC	0031226, 0005887, 0009275, 0009986
Lmo0069	Protein of unknown function	ssIMP III	YidC	0031226, 0005887, 0009986
Lmo0504	Protein of unknown function	ssIMP III	YidC	0031226, 0005887, 0009986
Lmo0545	Transcriptional activator	ssIMP III	YidC	0031226, 0005887, 0009986
Lmo0825	Hydroxymethylglutaryl-CoA reductase	ssIMP III	YidC	0031226, 0005887, 0009986
Lmo1088	Glycosyl/glycerophosphate transferases involved in teichoic acid biosynthesis, TagB	ssIMP III	YidC	0031226, 0005887, 0009986
Lmo1238	Ribonuclease PH	ssIMP III	YidC	0031226, 0005887, 0009986
Lmo1306	Protein of unknown function, COG3763	ssIMP III	YidC	0031226, 0005887, 0009986
Lmo1351	Rhodanese-related sulfurtransferase	ssIMP III	YidC	0031226, 0005887, 0009986
Lmo1412	Topology modulation protein	ssIMP III	YidC	0031226, 0005887, 0009986
Lmo1538	Glycerol kinase	ssIMP III	YidC	0031226, 0005887, 0009986
Lmo1594	Negative regulator of septation ring formation	ssIMP III	YidC	0031226, 0005887, 0009986
Lmo1885	Xanthine phosphoribosyltransferase	ssIMP III	YidC	0031226, 0005887, 0009986
Lmo2302	Protein of unknown function	ssIMP III	YidC	0031226, 0005887, 0009986
Lmo2325	Protein of unknown function	ssIMP III	YidC	0031226, 0005887, 0009986
Lmo2533	F_0F_1 ATP synthase, subunit B	ssIMP III	YidC	0031226, 0005887, 0009986

Lmo2664	Threonine dehydrogenase, Zn-dependent dehydrogenase	ssIMP III	YidC	0031226, 0005887, 0009986
Lmo2836	Threonine dehydrogenase, Zn-dependent dehydrogenase	ssIMP III	YidC	0031226, 0005887, 0009986
Multi-spanning integral membrane proteins				
Lmo0014	Cytochrome AA3-600 quinol oxidase, subunit I	msIMP	YidC	0031226, 0005887, 0009986
Lmo0024	Phosphotransferase system, mannose/fructose/sorbose/N-acetylgalactosamine IID	msIMP	YidC	0031226, 0005887, 0009986
Lmo0027	Phosphotransferase system, β-glucoside-specific IIABC component	msIMP	YidC	0031226, 0005887, 0009986
Lmo0048	Post-translational modification protein of the autoinducing quorum-sensing peptide	msIMP	YidC	0031226, 0005887, 0009986
Lmo0061	WXG100 protein secretion system, ATPase component, YukAB (EssC)	msIMP	YidC	0031226, 0005887, 0009986
Lmo0098	Phosphotransferase system, mannose/fructose/sorbose family IID component	msIMP	YidC	0031226, 0005887, 0009986
Lmo0121	Phage-related protein	msIMP	YidC	0031226, 0005887, 0009986
Lmo0155	ABC-type Mn2+/Zn2+ transport system, permease component	msIMP	YidC	0031226, 0005887, 0009986
Lmo0176	Sugar uptake permease	msIMP	YidC	0031226, 0005887, 0009986
Lmo0321	Protein of unknown function	msIMP	YidC	0031226, 0005887, 0009986
Lmo0332	Protein of unknown function	msIMP	YidC	0031226, 0005887, 0009986
Lmo0349	Protein of unknown function	msIMP	YidC	0031226, 0005887, 0009986
Lmo0358	Phosphotransferase system, fructose IIC component	msIMP	YidC	0031226, 0005887, 0009986
Lmo0405	Phosphate transporter	msIMP	YidC	0031226, 0005887, 0009986
Lmo0424	Sugar transport	msIMP	YidC	0031226, 0005887, 0009986
Lmo0459	M trans-acting positive regulator	msIMP	YidC	0031226, 0005887, 0009986
Lmo0508	Phosphotransferase system, galactitol-specific IIC component	msIMP	YidC	0031226, 0005887, 0009986
Lmo0518	Protein of unknown function with DUF1304, COG3759	msIMP	YidC	0031226, 0005887, 0009986
Lmo0529	Glycosyltransferases, probably involved in cell wall biogenesis	msIMP	YidC	0031226, 0005887, 0009986
Lmo0543	Phosphotransferase system sorbitol-specific component IIBC	msIMP	YidC	0031226, 0005887, 0009986
Lmo0559	Mg2+ and Co2+ transporters	msIMP	YidC	0031226, 0005887, 0009986
Lmo0577	Protein of unknown function, COG3610	msIMP	YidC	0031226, 0005887, 0009986
Lmo0578	Threonine/serine exporter family, ThrE	msIMP	YidC	0031226, 0005887, 0009986
Lmo0589	Protein of unknown function, COG4852	msIMP	YidC	0031226, 0005887, 0009986
Lmo0604	Protein of unknown function	msIMP	YidC	0031226, 0005887, 0009986
Lmo0618	Protein kinase-like	msIMP	YidC	0031226, 0005887, 0009986
Lmo0647	Protein of unknown function	msIMP	YidC	0031226, 0005887, 0009986
Lmo0648	Mg2+ and Co2+ transporters	msIMP	YidC	0031226, 0005887, 0009986
Lmo0722	Pyruvate oxidase	msIMP	YidC	0031226, 0005887, 0009986

Lmo0733	Lambda repressor-like, DNA-binding	msIMP	YidC	0031226, 0005887, 0009986
Lmo0738	β-glucoside-specific PTS system components IIABC	msIMP	YidC	0031226, 0005887, 0009986
Lmo0744	ABC-type antimicrobial peptide transport system, ATPase component	msIMP	YidC	0031226, 0005887, 0009986
Lmo0779	Protein of unknown function with DUF986 domain, COG4811	msIMP	YidC	0031226, 0005887, 0009986
Lmo0781	Phosphotransferase system, mannose/fructose/sorbose family IID component	msIMP	YidC	0031226, 0005887, 0009986
Lmo0793	Protein of unknown function with DUF554 domain, COG1811	msIMP	YidC	0031226, 0005887, 0009986
Lmo0803	Na+/H+ antiporter	msIMP	YidC	0031226, 0005887, 0009986
Lmo0826	Na+/phosphate symporter	msIMP	YidC	0031226, 0005887, 0009986
Lmo0867	Protein of unknown function with DUF988 domain, COG4708	msIMP	YidC	0031226, 0005887, 0009986
Lmo0910	Protein of unknown function, UCP032908-like, COG4194	msIMP	YidC	0031226, 0005887, 0009986
Lmo0933	Undecaprenyl phosphate 4-deoxy-4-formamido-L-arabinose transferase	msIMP	YidC	0031226, 0005887, 0009986
Lmo0992	Tellurium resistance membrane protein, TerC	msIMP	YidC	0031226, 0005887, 0009986
Lmo0993	Cation transporter	msIMP	YidC	0031226, 0005887, 0009986
Lmo1013	Mechanosensitive ion channel MscS	msIMP	YidC	0031226, 0005887, 0009986
Lmo1015	ABC-type proline/glycine betaine transport system, permease component, GbuB	msIMP	YidC	0031226, 0005887, 0009986
Lmo1035	Phosphotransferase system, beta-glucoside-specific IIABC component	msIMP	YidC	0031226, 0005887, 0009986
Lmo1064	Mg2+ and Co2+ transporters, CorA-like	msIMP	YidC	0031226, 0005887, 0009986
Lmo1167	Aquaporin, glycerol uptake facilitator, GlpF	msIMP	YidC	0031226, 0005887, 0009986
Lmo1214	Protein of unknown function	msIMP	YidC	0031226, 0005887, 0009986
Lmo1230	Colicin V production protein	msIMP	YidC	0031226, 0005887, 0009986
Lmo1255	Phosphotransferase system, trehalose-specific IIBC component	msIMP	YidC	0031226, 0005887, 0009986
Lmo1261	Protein of unknown function with GYF domain	msIMP	YidC	0031226, 0005887, 0009986
Lmo1284	Protein of unknown function with DUF205 domain, COG0344	msIMP	YidC	0031226, 0005887, 0009986
Lmo1318	Peptidase M50, putative membrane-associated zinc metallopeptidase	msIMP	YidC	0031226, 0005887, 0009986
Lmo1337	Peptidase S54, rhomboid	msIMP	YidC	0031226, 0005887, 0009986
Lmo1346	Fimbrilin-protein exporter, membrane component, ComGB	msIMP	YidC	0031226, 0005887, 0009986
Lmo1353	Protein of unknown function with DUF1385 domain, COG3872	msIMP	YidC	0031226, 0005887, 0009986
Lmo1422	ABC-type proline/glycine betaine system, oxgall and bile exclusion system, BilEB	msIMP	YidC	0031226, 0005887, 0009986
Lmo1425	ABC-type proline/glycine betaine transport systems, permease component, OpuCD	msIMP	YidC	0031226, 0005887, 0009986
Lmo1427	ABC-type proline/glycine betaine transport systems, permease component, OpuCB	msIMP	YidC	0031226, 0005887, 0009986
Lmo1443	Branched-chain amino acid transporter	msIMP	YidC	0031226, 0005887, 0009986
Lmo1446	ABC-type Mn2+/Zn2+ transport systems, permease component, ZurM	msIMP	YidC	0031226, 0005887, 0009986

Lmo1482	Competence protein ComEC/Rec2	msIMP	YidC	0031226, 0005887, 0009986
Lmo1500	Selenite transport protein, SNARE-associated protein	msIMP	YidC	0031226, 0005887, 0009986
Lmo1516	Ammonium permease	msIMP	YidC	0031226, 0005887, 0009986
Lmo1539	Major intrinsic protein	msIMP	YidC	0031226, 0005887, 0009986
Lmo1650	Protein of unknown function with DUF1453 domain, COG4846	msIMP	YidC	0031226, 0005887, 0009986
Lmo1665	Protein of unknown function	msIMP	YidC	0031226, 0005887, 0009986
Lmo1740	ABC-type amino acid transport system, permease component	msIMP	YidC	0031226, 0005887, 0009986
Lmo1776	Protein of unknown function, COG4843	msIMP	YidC	0031226, 0005887, 0009986
Lmo1812	L-serine dehydratase, alpha subunit	msIMP	YidC	0031226, 0005887, 0009986
Lmo1844	Signal peptidase of Type II, lipoprotein signal peptidase, LspA	msIMP	YidC	0031226, 0005887, 0009986
Lmo1853	Heavy metal translocating P-type ATPase	msIMP	YidC	0031226, 0005887, 0009986
Lmo1911	Protein with GGDEF domain	msIMP	YidC	0031226, 0005887, 0009986
Lmo1912	Protein with GGDEF domain	msIMP	YidC	0031226, 0005887, 0009986
Lmo1919	Zinc metallopeptidase	msIMP	YidC	0031226, 0005887, 0009986
Lmo1971	Sugar-specific PTS, UlaA	msIMP	YidC	0031226, 0005887, 0009986
Lmo1999	Glucosamine 6-phosphate synthetase, amidotransferase and phosphosugar isomerase	msIMP	YidC	0031226, 0005887, 0009986
Lmo2000	Phosphotransferase system, mannose/fructose/sorbose family IID component	msIMP	YidC	0031226, 0005887, 0009986
Lmo2029	Protein of unknown function, YGGT-like, COG0762	msIMP	YidC	0031226, 0005887, 0009986
Lmo2037	UDP-N- phosphotransferase, MraY	msIMP	YidC	0031226, 0005887, 0009986
Lmo2075	O-sialoglycoprotein endopeptidase	msIMP	YidC	0031226, 0005887, 0009986
Lmo2081	Camphor resistance CrcB protein	msIMP	YidC	0031226, 0005887, 0009986
Lmo2096	Phosphotransferase system, galactitol-specific IIC component	msIMP	YidC	0031226, 0005887, 0009986
Lmo2105	Fe2+ transport system protein B	msIMP	YidC	0031226, 0005887, 0009986
Lmo2120	Protein of unknown function with DUF147 domain, CHP00159-like, COG1624	msIMP	YidC	0031226, 0005887, 0009986
Lmo2170	2-nitropropane dioxygenase, NPD	msIMP	YidC	0031226, 0005887, 0009986
Lmo2174	Protein with GGDEF domain	msIMP	YidC	0031226, 0005887, 0009986
Lmo2221	Protein of unknown function, P-loop nucleoside triphosphate hydrolases, COG4717	msIMP	YidC	0031226, 0005887, 0009986
Lmo2224	Protein of unknown function with DUF445 domain, UCP032178-like, COG4399	msIMP	YidC	0031226, 0005887, 0009986
Lmo2287	Tape measure domain bacteriophage A118	msIMP	YidC	0031226, 0005887, 0009986
Lmo2335	2-O-a-mannosyl-D-glycerate specific PTS transporten components IIABC	msIMP	YidC	0031226, 0005887, 0009986
Lmo2347	ABC-type amino acid transport system, permease component	msIMP	YidC	0031226, 0005887, 0009986
Lmo2367	Phosphoglucose isomerase	msIMP	YidC	0031226, 0005887, 0009986

Lmo2378	Na(+)/H(+) antiporter subunit A, MnhA	msIMP	YidC	0031226, 0005887, 0009986
Lmo2380	Multisubunit Na+/H+ antiporter, MnhB subunit	msIMP	YidC	0031226, 0005887, 0009986
Lmo2381	Monovalent cation/H+ antiporter subuni D	msIMP	YidC	0031226, 0005887, 0009986
Lmo2383	Monovalent cation/H+ antiporter subunit F	msIMP	YidC	0031226, 0005887, 0009986
Lmo2482	Prolipoprotein diacylglyceryltransferase, Lgt	msIMP	YidC	0031226, 0005887, 0009986
Lmo2490	Protein of unknown function, COG4897	msIMP	YidC	0031226, 0005887, 0009986
Lmo2502	PDZ/DHR/GLGF	msIMP	YidC	0031226, 0005887, 0009986
Lmo2519	Glycosyl transferase, family 4	msIMP	YidC	0031226, 0005887, 0009986
Lmo2526	UDP-N-acetylglucosamine enolpyruvyl transferase	msIMP	YidC	0031226, 0005887, 0009986
Lmo2550	Undecaprenyl phosphate 4-deoxy-4-formamido-L-arabinose transferase	msIMP	YidC	0031226, 0005887, 0009986
Lmo2602	MgtC/SapB transporter	msIMP	YidC	0031226, 0005887, 0009986
Lmo2638	NADH dehydrogenase, FAD-containing subunit	msIMP	YidC	0031226, 0005887, 0009986
Lmo2641	Terpenoid synthase	msIMP	YidC	0031226, 0005887, 0009986
Lmo2649	Sugar-specific PTS, UlaA	msIMP	YidC	0031226, 0005887, 0009986
Lmo2665	Phosphotransferase system, galactitol-specific IIC component	msIMP	YidC	0031226, 0005887, 0009986
Lmo2669	Protein of unknown function with DUF1113 domain, COG4905	msIMP	YidC	0031226, 0005887, 0009986
Lmo2679	Osmosensitive K+ channel histidine kinase	msIMP	YidC	0031226, 0005887, 0009986
Lmo2682	K+ transporting ATPase, A subunit, KdpA	msIMP	YidC	0031226, 0005887, 0009986
Lmo2717	Cytochrome bd-type quinol oxidase, subunit 2, CydB	msIMP	YidC	0031226, 0005887, 0009986
Lmo2718	Cytochrome bd-type quinol oxidase, subunit 1, CydA	msIMP	YidC	0031226, 0005887, 0009986
Lmo2733	2-O-a-mannosyl-D-glycerate specific PTS transporten components IIABC	msIMP	YidC	0031226, 0005887, 0009986
Lmo2767	Protein of unknown function, UCP033111-like, COG4858	msIMP	YidC	0031226, 0005887, 0009986
Lmo2772	Phosphotransferase system, beta-glucoside-specific IIABC component	msIMP	YidC	0031226, 0005887, 0009986
Lmo2778	Protein of unknown function	msIMP	YidC	0031226, 0005887, 0009986
Lmo2787	Phosphotransferase system, β-glucoside-specific IIABC component, BvrB	msIMP	YidC	0031226, 0005887, 0009986
Lmo0003	Na+-driven multi antimicrobial extrusion protein MatE	msIMP, Unc-SP	Sec, YidC	0031226, 0005887, 0009986
Lmo0008	Cardiolipin synthetase	msIMP, Unc-SP	Sec, YidC	0031226, 0005887, 0009986
Lmo0015	Cytochrome AA3-600 quinol oxidase, subunit III	msIMP, Unc-SP	Sec, YidC	0031226, 0005887, 0009986
Lmo0016	Cytochrome AA3-600 quinol oxidase, subunit IV, QoxD	msIMP, Unc-SP	Sec, YidC	0031226, 0005887, 0009986
Lmo0023	Phosphotransferase system, mannose/fructose/sorbose/N-acetylgalactosamine IIC	msIMP, Unc-SP	Sec, YidC	0031226, 0005887, 0009986
Lmo0034	Phosphotransferase system, lactose/cellobiose IIC component	msIMP, Unc-SP	Sec, YidC	0031226, 0005887, 0009986
Lmo0042	Selenite transport protein, SNARE-associated protein, DedA	msIMP, Unc-SP	Sec, YidC	0031226, 0005887, 0009986

Lmo0050	Signal transduction protein with a C-terminal ATPase domain	msIMP, Unc-SP	Sec, YidC	0031226, 0005887, 0009986
Lmo0070	Protein of unknown function	msIMP, Unc-SP	Sec, YidC	0031226, 0005887, 0009986
Lmo0073	Protein of unknown function	msIMP, Unc-SP	Sec, YidC	0031226, 0005887, 0009986
Lmo0074	Protein of unknown function	msIMP, Unc-SP	Sec, YidC	0031226, 0005887, 0009986
Lmo0088	F0F1-type ATP synthase, subunit c	msIMP, Unc-SP	Sec, YidC	0031226, 0005887, 0009986
Lmo0094	Protein of unknown function	msIMP, Unc-SP	Sec, YidC	0031226, 0005887, 0009986
Lmo0097	Phosphotransferase system, mannose/fructose/sorbose family IIC component	msIMP, Unc-SP	Sec, YidC	0031226, 0005887, 0009986
Lmo0107	ABC-type bacteriocin/lantibiotic exporters, ATP-binding/permease protein	msIMP, Unc-SP	Sec, YidC	0031226, 0005887, 0009986
Lmo0108	ABC-type bacteriocin/lantibiotic exporters, ATP-binding/permease protein	msIMP, Unc-SP	Sec, YidC	0031226, 0005887, 0009986
Lmo0128	Holin TcdE-like	msIMP, Unc-SP	Sec, YidC	0031226, 0005887, 0009986
Lmo0137	ABC-type dipeptide/oligopeptide/nickel transport system, permease component	msIMP, Unc-SP	Sec, YidC	0031226, 0005887, 0009986
Lmo0180	ABC-type sugar transport system, permease component	msIMP, Unc-SP	Sec, YidC	0031226, 0005887, 0009986
Lmo0195	ABC-type macrolide transport system, permease component	msIMP, Unc-SP	Sec, YidC	0031226, 0005887, 0009986
Lmo0215	Polysaccharide biosynthesis protein	msIMP, Unc-SP	Sec, YidC	0031226, 0005887, 0009986
Lmo0220	Peptidase M41, FtsH	msIMP, Unc-SP	Sec, YidC	0031226, 0005887, 0009986
Lmo0253	Peptidase M56, BlaR1	msIMP, Unc-SP	Sec, YidC	0031226, 0005887, 0009986
Lmo0283	ABC-type metal ion transport system, permease component	msIMP, Unc-SP	Sec, YidC	0031226, 0005887, 0009986
Lmo0288	Signal transduction histidine kinase	msIMP, Unc-SP	Sec, YidC	0031226, 0005887, 0009986
Lmo0296	Protein of unknown function	msIMP, Unc-SP	Sec, YidC	0031226, 0005887, 0009986
Lmo0298	Phosphotransferase system cellobiose-specific component IIC	msIMP, Unc-SP	Sec, YidC	0031226, 0005887, 0009986
Lmo0350	Protein of unknown function	msIMP, Unc-SP	Sec, YidC	0031226, 0005887, 0009986
Lmo0361	Twin-arginine translocase protein C, TatC	msIMP, Unc-SP	Sec, YidC	0031226, 0005887, 0009986
Lmo0373	Phosphotransferase system cellobiose-specific component IIC	msIMP, Unc-SP	Sec, YidC	0031226, 0005887, 0009986
Lmo0388	Protein of unknown function	msIMP, Unc-SP	Sec, YidC	0031226, 0005887, 0009986
Lmo0389	Low temperature requirement protein A, LtrA	msIMP, Unc-SP	Sec, YidC	0031226, 0005887, 0009986
Lmo0400	Phosphotransferase system, fructose-specific IIC component	msIMP, Unc-SP	Sec, YidC	0031226, 0005887, 0009986
Lmo0403	Protein of unknown function	msIMP, Unc-SP	Sec, YidC	0031226, 0005887, 0009986
Lmo0417	Protein of unknown function	msIMP, Unc-SP	Sec, YidC	0031226, 0005887, 0009986
Lmo0419	Protein of unknown function with DUF1211 and , COG3548	msIMP, Unc-SP	Sec, YidC	0031226, 0005887, 0009986
Lmo0421	Cell division membrane protein	msIMP, Unc-SP	Sec, YidC	0031226, 0005887, 0009986
Lmo0428	Phosphotransferase system, fructose-specific IIC component	msIMP, Unc-SP	Sec, YidC	0031226, 0005887, 0009986
Lmo0450	Protein of unknown function with DUF1275 and , COG3619	msIMP, Unc-SP	Sec, YidC	0031226, 0005887, 0009986

Lmo0452	Transglutaminase-like	msIMP, Unc-SP	Sec, YidC	0031226, 0005887, 0009986
Lmo0453	Protein of unknown function with DUF58 domain, COG1721	msIMP, Unc-SP	Sec, YidC	0031226, 0005887, 0009986
Lmo0456	Purine-cytosine permease and related proteins	msIMP, Unc-SP	Sec, YidC	0031226, 0005887, 0009986
Lmo0475	Protein of unknown function	msIMP, Unc-SP	Sec, YidC	0031226, 0005887, 0009986
Lmo0495	Protein of unknown function with DUF6, COG0697	msIMP, Unc-SP	Sec, YidC	0031226, 0005887, 0009986
Lmo0519	Drug resistance transporter EmrB/QacA subfamily	msIMP, Unc-SP	Sec, YidC	0031226, 0005887, 0009986
Lmo0523	Protein of unknown function	msIMP, Unc-SP	Sec, YidC	0031226, 0005887, 0009986
Lmo0524	Sulfate permease, transporter MFS superfamily	msIMP, Unc-SP	Sec, YidC	0031226, 0005887, 0009986
Lmo0527	Protein of unknown function	msIMP, Unc-SP	Sec, YidC	0031226, 0005887, 0009986
Lmo0531	Protein of unknown function and GGDEF domain	msIMP, Unc-SP	Sec, YidC	0031226, 0005887, 0009986
Lmo0544	Phosphotransferase system, enzyme II sorbitol-specific factor	msIMP, Unc-SP	Sec, YidC	0031226, 0005887, 0009986
Lmo0555	Amino acid/peptide transporter	msIMP, Unc-SP	Sec, YidC	0031226, 0005887, 0009986
Lmo0573	Xanthine/uracil/vitamin C permease	msIMP, Unc-SP	Sec, YidC	0031226, 0005887, 0009986
Lmo0584	Autoinducer-2 exporter family, AI-2E (PerM)	msIMP, Unc-SP	Sec, YidC	0031226, 0005887, 0009986
Lmo0593	Formate/nitrite family of transporters	msIMP, Unc-SP	Sec, YidC	0031226, 0005887, 0009986
Lmo0596	Acid-resistance membrane protein, COG3247	msIMP, Unc-SP	Sec, YidC	0031226, 0005887, 0009986
Lmo0600	Protein of unknown function with DUF1700 domain and , COG3752	msIMP, Unc-SP	Sec, YidC	0031226, 0005887, 0009986
Lmo0603	Protein of unknown function	msIMP, Unc-SP	Sec, YidC	0031226, 0005887, 0009986
Lmo0605	Na+-driven multidrug efflux pump	msIMP, Unc-SP	Sec, YidC	0031226, 0005887, 0009986
Lmo0607	ABC-type bacteriocin/lantibiotic exporters, ATP-binding/permease protein	msIMP, Unc-SP	Sec, YidC	0031226, 0005887, 0009986
Lmo0608	ABC-type bacteriocin/lantibiotic exporters, ATP-binding/permease protein	msIMP, Unc-SP	Sec, YidC	0031226, 0005887, 0009986
Lmo0616	Glycerophosphoryl diester phosphodiesterase	msIMP, Unc-SP	Sec, YidC	0031226, 0005887, 0009986
Lmo0621	Permease, COG0730	msIMP, Unc-SP	Sec, YidC	0031226, 0005887, 0009986
Lmo0622	Protein of unknown function	msIMP, Unc-SP	Sec, YidC	0031226, 0005887, 0009986
Lmo0639	Protein of unknown function	msIMP, Unc-SP	Sec, YidC	0031226, 0005887, 0009986
Lmo0642	Protein of unknown function	msIMP, Unc-SP	Sec, YidC	0031226, 0005887, 0009986
Lmo0644	Membrane-bound phosphoglycerol transferase, alkaline phosphatase superfamily	msIMP, Unc-SP	Sec, YidC	0031226, 0005887, 0009986
Lmo0645	Amino acid/polyamine transporter I	msIMP, Unc-SP	Sec, YidC	0031226, 0005887, 0009986
Lmo0653	Protein of unknown function with DUF975 domain, COG5523	msIMP, Unc-SP	Sec, YidC	0031226, 0005887, 0009986
Lmo0656	Protein of unknown function with DUF420 domain, COG2322	msIMP, Unc-SP	Sec, YidC	0031226, 0005887, 0009986
Lmo0668	ABC-type polysaccharide/polyol phosphate export system, permease component	msIMP, Unc-SP	Sec, YidC	0031226, 0005887, 0009986
Lmo0672	Protein of unknown function with DUF805 domain and , COG3152	msIMP, Unc-SP	Sec, YidC	0031226, 0005887, 0009986

Lmo0677	Flagellar export apparatus, membrane subunit FliQ	msIMP, Unc-SP	Sec, YidC	0031226, 0005887, 0009986
Lmo0679	Flagellar export apparatus, membrane subunit FlhB	msIMP, Unc-SP	Sec, YidC	0031226, 0005887, 0009986
Lmo0680	Flagellar export apparatus, membrane subunit FlhA	msIMP, Unc-SP	Sec, YidC	0031226, 0005887, 0009986
Lmo0685	Flagellar motor protein MotA	msIMP, Unc-SP	Sec, YidC	0031226, 0005887, 0009986
Lmo0713	Flagellar MS-ring protein, FliF	msIMP, Unc-SP	Sec, YidC	0031226, 0005887, 0009986
Lmo0723	Chemotaxis methyl-accepting receptor, signalling	msIMP, Unc-SP	Sec, YidC	0031226, 0005887, 0009986
Lmo0731	Protein of unknown function	msIMP, Unc-SP	Sec, YidC	0031226, 0005887, 0009986
Lmo0743	Protein of unknown function	msIMP, Unc-SP	Sec, YidC	0031226, 0005887, 0009986
Lmo0748	Protein of unknown function	msIMP, Unc-SP	Sec, YidC	0031226, 0005887, 0009986
Lmo0750	Protein of unknown function	msIMP, Unc-SP	Sec, YidC	0031226, 0005887, 0009986
Lmo0757	ABC-type transport system, multi-copper enzyme maturation, permease component	msIMP, Unc-SP	Sec, YidC	0031226, 0005887, 0009986
Lmo0766	ABC-type sugar transport systems, permease components	msIMP, Unc-SP	Sec, YidC	0031226, 0005887, 0009986
Lmo0771	Protein of unknown function	msIMP, Unc-SP	Sec, YidC	0031226, 0005887, 0009986
Lmo0782	Phosphotransferase system, sorbose-specific IIC subunit	msIMP, Unc-SP	Sec, YidC	0031226, 0005887, 0009986
Lmo0787	D-alanine/D-serine/glycine permease	msIMP, Unc-SP	Sec, YidC	0031226, 0005887, 0009986
Lmo0795	Chloramphenicol-sensitive protein, RarD	msIMP, Unc-SP	Sec, YidC	0031226, 0005887, 0009986
Lmo0798	lysine transporter	msIMP, Unc-SP	Sec, YidC	0031226, 0005887, 0009986
Lmo0804	Protein of unknown function	msIMP, Unc-SP	Sec, YidC	0031226, 0005887, 0009986
Lmo0805	Protein of unknown function	msIMP, Unc-SP	Sec, YidC	0031226, 0005887, 0009986
Lmo0808	ABC-type spermidine/putrescine transport system, permease component I	msIMP, Unc-SP	Sec, YidC	0031226, 0005887, 0009986
Lmo0809	ABC-type spermidine/putrescine transport system, permease component II	msIMP, Unc-SP	Sec, YidC	0031226, 0005887, 0009986
Lmo0818	Cation transport ATPase	msIMP, Unc-SP	Sec, YidC	0031226, 0005887, 0009986
Lmo0836	Phosphate-starvation-inducible E	msIMP, Unc-SP	Sec, YidC	0031226, 0005887, 0009986
Lmo0838	Sugar phosphate antiporter, UhpT	msIMP, Unc-SP	Sec, YidC	0031226, 0005887, 0009986
Lmo0841	Cation transport ATPase	msIMP, Unc-SP	Sec, YidC	0031226, 0005887, 0009986
Lmo0843	Protein of unknown function with DUF1294 domain, COG3326	msIMP, Unc-SP	Sec, YidC	0031226, 0005887, 0009986
Lmo0853	Small multidrug resistance protein	msIMP, Unc-SP	Sec, YidC	0031226, 0005887, 0009986
Lmo0854	Small multidrug resistance protein	msIMP, Unc-SP	Sec, YidC	0031226, 0005887, 0009986
Lmo0861	ABC-type sugar transport system, permease component	msIMP, Unc-SP	Sec, YidC	0031226, 0005887, 0009986
Lmo0876	Phosphotransferase system cellobiose-specific component IIC	msIMP, Unc-SP	Sec, YidC	0031226, 0005887, 0009986
Lmo0882	Protein of unknown function with DUF304 domain and , COG3402	msIMP, Unc-SP	Sec, YidC	0031226, 0005887, 0009986
Lmo0883	Protein of unknown function , UCP026631-like, COG3428	msIMP, Unc-SP	Sec, YidC	0031226, 0005887, 0009986

Lmo0901	Phosphotransferase system, cellobiose-specific IIC component	msIMP, Unc-SP	Sec, YidC	0031226, 0005887, 0009986
Lmo0908	Autoinducer-2 Exporter family, AI-2E (PerM)	msIMP, Unc-SP	Sec, YidC	0031226, 0005887, 0009986
Lmo0912	Formate/nitrite transporter	msIMP, Unc-SP	Sec, YidC	0031226, 0005887, 0009986
Lmo0915	Phosphotransferase system cellobiose-specific component IIC	msIMP, Unc-SP	Sec, YidC	0031226, 0005887, 0009986
Lmo0920	Protein of unknown function with DUF318 domain and , COG0701	msIMP, Unc-SP	Sec, YidC	0031226, 0005887, 0009986
Lmo0925	ABC-type multidrug transport system, permease component	msIMP, Unc-SP	Sec, YidC	0031226, 0005887, 0009986
Lmo0927	Membrane-bound phosphoglycerol transferase, alkaline phosphatase superfamily	msIMP, Unc-SP	Sec, YidC	0031226, 0005887, 0009986
Lmo0932	Selenite transport protein, SNARE-associated protein, YdjX	msIMP, Unc-SP	Sec, YidC	0031226, 0005887, 0009986
Lmo0937	Protein of unknown function	msIMP, Unc-SP	Sec, YidC	0031226, 0005887, 0009986
Lmo0947	Cyanate permease	msIMP, Unc-SP	Sec, YidC	0031226, 0005887, 0009986
Lmo0949	Protein of unknown function, UPF0118-like, COG0628	msIMP, Unc-SP	Sec, YidC	0031226, 0005887, 0009986
Lmo0952	Protein of unknown function	msIMP, Unc-SP	Sec, YidC	0031226, 0005887, 0009986
Lmo0954	Protein of unknown function	msIMP, Unc-SP	Sec, YidC	0031226, 0005887, 0009986
Lmo0963	Heat shock protein HtpX	msIMP, Unc-SP	Sec, YidC	0031226, 0005887, 0009986
Lmo0980	ABC-type polysaccharide/polyol phosphate export system, permease component	msIMP, Unc-SP	Sec, YidC	0031226, 0005887, 0009986
Lmo0985	MFS general substrate transporter	msIMP, Unc-SP	Sec, YidC	0031226, 0005887, 0009986
Lmo0987	Multidrug/hemolysin exporter, CylA/B	msIMP, Unc-SP	Sec, YidC	0031226, 0005887, 0009986
Lmo0990	Na+-driven multidrug efflux pump	msIMP, Unc-SP	Sec, YidC	0031226, 0005887, 0009986
Lmo0994	Protein of unknown function	msIMP, Unc-SP	Sec, YidC	0031226, 0005887, 0009986
Lmo0995	Fucose 4-O-acetylase and related acetyltransferases	msIMP, Unc-SP	Sec, YidC	0031226, 0005887, 0009986
Lmo0998	Abortive infection protein	msIMP, Unc-SP	Sec, YidC	0031226, 0005887, 0009986
Lmo0999	Protein of unknown function	msIMP, Unc-SP	Sec, YidC	0031226, 0005887, 0009986
Lmo1020	Cell wall-active antibiotics response protein	msIMP, Unc-SP	Sec, YidC	0031226, 0005887, 0009986
Lmo1024	Protein of unknown function	msIMP, Unc-SP	Sec, YidC	0031226, 0005887, 0009986
Lmo1025	Protein of unknown function, COG1289	msIMP, Unc-SP	Sec, YidC	0031226, 0005887, 0009986
Lmo1037	Protein of unknown function with DUF817 domain, COG3739	msIMP, Unc-SP	Sec, YidC	0031226, 0005887, 0009986
Lmo1056	Protein of unknown function	msIMP, Unc-SP	Sec, YidC	0031226, 0005887, 0009986
Lmo1061	Signal transduction histidine kinase, BaeS	msIMP, Unc-SP	Sec, YidC	0031226, 0005887, 0009986
Lmo1062	ABC-type transport system involved in lipoprotein release, permease component	msIMP, Unc-SP	Sec, YidC	0031226, 0005887, 0009986
Lmo1071	Cell cycle protein	msIMP, Unc-SP	Sec, YidC	0031226, 0005887, 0009986
Lmo1074	ABC-type polysaccharide/polyol, permease component, COG1682	msIMP, Unc-SP	Sec, YidC	0031226, 0005887, 0009986
Lmo1079	Protein of unknown function, COG4485	msIMP, Unc-SP	Sec, YidC	0031226, 0005887, 0009986

Lmo1100	Heavy metal translocating P-type ATPase, CadA	msIMP, Unc-SP	Sec, YidC	0031226, 0005887, 0009986
Lmo1101	Signal peptidase of Type II, LspB	msIMP, Unc-SP	Sec, YidC	0031226, 0005887, 0009986
Lmo1105	Protein of unknown function	msIMP, Unc-SP	Sec, YidC	0031226, 0005887, 0009986
Lmo1107	Transfer Clostridial Plasmid Conjugative System, Tcp-Conj protein family	msIMP, Unc-SP	Sec, YidC	0031226, 0005887, 0009986
Lmo1109	Protein of unknown function	msIMP, Unc-SP	Sec, YidC	0031226, 0005887, 0009986
Lmo1112	Cell divisionFtsK/SpoIIIE	msIMP, Unc-SP	Sec, YidC	0031226, 0005887, 0009986
Lmo1132	ABC-type transport system cysteine/glutathione, ATP-binding component	msIMP, Unc-SP	Sec, YidC	0031226, 0005887, 0009986
Lmo1148	Cobalamin synthase	msIMP, Unc-SP	Sec, YidC	0031226, 0005887, 0009986
Lmo1186	Ethanolamine utilisation protein, EutH	msIMP, Unc-SP	Sec, YidC	0031226, 0005887, 0009986
Lmo1192	Cobalamin biosynthesis protein	msIMP, Unc-SP	Sec, YidC	0031226, 0005887, 0009986
Lmo1206	Cobalt ABC transporter CbiQ, permease subunit, CbiQ	msIMP, Unc-SP	Sec, YidC	0031226, 0005887, 0009986
Lmo1224	ABC-type antimicrobial peptide transport system, permease component	msIMP, Unc-SP	Sec, YidC	0031226, 0005887, 0009986
Lmo1250	Major facilitator superfamily MFS-1	msIMP, Unc-SP	Sec, YidC	0031226, 0005887, 0009986
Lmo1291	Acyltransferase family 3	msIMP, Unc-SP	Sec, YidC	0031226, 0005887, 0009986
Lmo1352	Protein of unknown function	msIMP, Unc-SP	Sec, YidC	0031226, 0005887, 0009986
Lmo1385	Protein of unknown function with DUF161 domain and , COG1284	msIMP, Unc-SP	Sec, YidC	0031226, 0005887, 0009986
Lmo1386	DNA segregation ATPase FtsK/SpoIIIE and related proteins	msIMP, Unc-SP	Sec, YidC	0031226, 0005887, 0009986
Lmo1390	ABC-type uncharacterized transport system, permease component	msIMP, Unc-SP	Sec, YidC	0031226, 0005887, 0009986
Lmo1396	CDP-diacylglycerol--glycerol-3-phosphate 3-phosphatidyltransferase	msIMP, Unc-SP	Sec, YidC	0031226, 0005887, 0009986
Lmo1409	MFS general substrate transporter	msIMP, Unc-SP	Sec, YidC	0031226, 0005887, 0009986
Lmo1410	Protein of unknown function	msIMP, Unc-SP	Sec, YidC	0031226, 0005887, 0009986
Lmo1417	Permease of the major facilitator superfamily	msIMP, Unc-SP	Sec, YidC	0031226, 0005887, 0009986
Lmo1419	Autoinducer-2 exporter family, AI-2E (PerM)	msIMP, Unc-SP	Sec, YidC	0031226, 0005887, 0009986
Lmo1424	Mn2+ and Fe2+ transporters of the NRAMP family, MntH-like	msIMP, Unc-SP	Sec, YidC	0031226, 0005887, 0009986
Lmo1429	Thiamine transporter YuaJ	msIMP, Unc-SP	Sec, YidC	0031226, 0005887, 0009986
Lmo1432	Cytochrome c oxidase, subunit I	msIMP, Unc-SP	Sec, YidC	0031226, 0005887, 0009986
Lmo1440	Protein of unknown function with DUF1189 domain	msIMP, Unc-SP	Sec, YidC	0031226, 0005887, 0009986
Lmo1442	AzlC-like	msIMP, Unc-SP	Sec, YidC	0031226, 0005887, 0009986
Lmo1464	Diacylglycerol kinase	msIMP, Unc-SP	Sec, YidC	0031226, 0005887, 0009986
Lmo1466	Membrane-associated HD superfamily hydrolase	msIMP, Unc-SP	Sec, YidC	0031226, 0005887, 0009986
Lmo1506	Macrolide exporter family, MacB	msIMP, Unc-SP	Sec, YidC	0031226, 0005887, 0009986
Lmo1508	Signal transduction histidine kinase	msIMP, Unc-SP	Sec, YidC	0031226, 0005887, 0009986

Lmo1526	Protein of unknown function, COG5416	msIMP, Unc-SP	Sec, YidC	0031226, 0005887, 0009986
Lmo1546	Cell shape-determining protein, MreD	msIMP, Unc-SP	Sec, YidC	0031226, 0005887, 0009986
Lmo1550	Type 4 prepilin peptidase, ComC	msIMP, Unc-SP	Sec, YidC	0031226, 0005887, 0009986
Lmo1569	Protein affecting phage T7 exclusion by the F plasmid, FxsA	msIMP, Unc-SP	Sec, YidC	0031226, 0005887, 0009986
Lmo1584	Protein with RDD domain	msIMP, Unc-SP	Sec, YidC	0031226, 0005887, 0009986
Lmo1617	Drug resistance transporter EmrB/QacA subfamily	msIMP, Unc-SP	Sec, YidC	0031226, 0005887, 0009986
Lmo1624	Polysaccharide biosynthesis protein	msIMP, Unc-SP	Sec, YidC	0031226, 0005887, 0009986
Lmo1625	Polysaccharide biosynthesis protein	msIMP, Unc-SP	Sec, YidC	0031226, 0005887, 0009986
Lmo1626	Protein of unknown function	msIMP, Unc-SP	Sec, YidC	0031226, 0005887, 0009986
Lmo1637	ABC-type transport system, multi-copper enzyme maturation, permease component	msIMP, Unc-SP	Sec, YidC	0031226, 0005887, 0009986
Lmo1640	Protein of unknown function	msIMP, Unc-SP	Sec, YidC	0031226, 0005887, 0009986
Lmo1651	ABC-type bacteriocin/lantibiotic exporters, ATP-binding/permease protein	msIMP, Unc-SP	Sec, YidC	0031226, 0005887, 0009986
Lmo1652	ABC-type bacteriocin/lantibiotic exporters, ATP-binding/permease protein	msIMP, Unc-SP	Sec, YidC	0031226, 0005887, 0009986
Lmo1655	VanZ like protein	msIMP, Unc-SP	Sec, YidC	0031226, 0005887, 0009986
Lmo1672	O-succinylbenzoic acid--CoA ligase	msIMP, Unc-SP	Sec, YidC	0031226, 0005887, 0009986
Lmo1677	1,4-dihydroxy-2-naphthoate octaprenyltransferase	msIMP, Unc-SP	Sec, YidC	0031226, 0005887, 0009986
Lmo1682	MFS general substrate transporter	msIMP, Unc-SP	Sec, YidC	0031226, 0005887, 0009986
Lmo1695	Lysyl-tRNA synthetase	msIMP, Unc-SP	Sec, YidC	0031226, 0005887, 0009986
Lmo1697	Co/Zn/Cd cation transporter	msIMP, Unc-SP	Sec, YidC	0031226, 0005887, 0009986
Lmo1706	Ribonuclease BN-related	msIMP, Unc-SP	Sec, YidC	0031226, 0005887, 0009986
Lmo1712	MFS general substrate transporter	msIMP, Unc-SP	Sec, YidC	0031226, 0005887, 0009986
Lmo1723	Protein of unknown function	msIMP, Unc-SP	Sec, YidC	0031226, 0005887, 0009986
Lmo1732	ABC-type sugar transport system, permease component	msIMP, Unc-SP	Sec, YidC	0031226, 0005887, 0009986
Lmo1741	Signal transduction histidine kinase	msIMP, Unc-SP	Sec, YidC	0031226, 0005887, 0009986
Lmo1746	ABC-type transport system, involved in lipoprotein release, permease component	msIMP, Unc-SP	Sec, YidC	0031226, 0005887, 0009986
Lmo1748	Protein of unknown function	msIMP, Unc-SP	Sec, YidC	0031226, 0005887, 0009986
Lmo1761	Na+-dependent transporters of the SNF family	msIMP, Unc-SP	Sec, YidC	0031226, 0005887, 0009986
Lmo1762	Protein of unknown function	msIMP, Unc-SP	Sec, YidC	0031226, 0005887, 0009986
Lmo1839	Xanthine/uracil permease, PyrP	msIMP, Unc-SP	Sec, YidC	0031226, 0005887, 0009986
Lmo1845	Hypoxanthine/guanosine uptake transporter, PbuG	msIMP, Unc-SP	Sec, YidC	0031226, 0005887, 0009986
Lmo1846	Multidrug efflux protein	msIMP, Unc-SP	Sec, YidC	0031226, 0005887, 0009986
Lmo1848	ABC-type iron (Fe^{2+})/zinc (Zn^{2+})/copper (Cu^{2+}) transprt system	msIMP, Unc-SP	Sec, YidC	0031226, 0005887, 0009986

Lmo1864	HylII	msIMP, Unc-SP	Sec, YidC	0031226, 0005887, 0009986
Lmo1869	Aromatic acid exporter family, ArAE	msIMP, Unc-SP	Sec, YidC	0031226, 0005887, 0009986
Lmo1870	Selenite transport protein, SNARE-associated protein	msIMP, Unc-SP	Sec, YidC	0031226, 0005887, 0009986
Lmo1884	Xanthine permease	msIMP, Unc-SP	Sec, YidC	0031226, 0005887, 0009986
Lmo1909	Protein of unknown function with DUF161 domain, COG1284	msIMP, Unc-SP	Sec, YidC	0031226, 0005887, 0009986
Lmo1920	Protein of unknown function with DUF1405 domain, COG4347	msIMP, Unc-SP	Sec, YidC	0031226, 0005887, 0009986
Lmo1947	Signal transduction histidine kinase, ResE	msIMP, Unc-SP	Sec, YidC	0031226, 0005887, 0009986
Lmo1957	ABC Fe3+-siderophore transport system, permease component, FhuG	msIMP, Unc-SP	Sec, YidC	0031226, 0005887, 0009986
Lmo1963	ABC-type transport system, multi-copper enzyme maturation, permease component	msIMP, Unc-SP	Sec, YidC	0031226, 0005887, 0009986
Lmo1966	5-bromo-4-chloroindolyl phosphate hydrolysis protein	msIMP, Unc-SP	Sec, YidC	0031226, 0005887, 0009986
Lmo1980	Protein of unknown function	msIMP, Unc-SP	Sec, YidC	0031226, 0005887, 0009986
Lmo2008	ABC-type sugar transport system, permease component	msIMP, Unc-SP	Sec, YidC	0031226, 0005887, 0009986
Lmo2011	Signal transduction protein with a C-terminal ATPase domain	msIMP, Unc-SP	Sec, YidC	0031226, 0005887, 0009986
Lmo2012	Protein of unknown function with DUF624 domain and , COG5578	msIMP, Unc-SP	Sec, YidC	0031226, 0005887, 0009986
Lmo2043	MFS general substrate transporter	msIMP, Unc-SP	Sec, YidC	0031226, 0005887, 0009986
Lmo2045	Protein of unknown function , UCP030092-like	msIMP, Unc-SP	Sec, YidC	0031226, 0005887, 0009986
Lmo2057	Protohaem IX farnesyltransferase, CtaB	msIMP, Unc-SP	Sec, YidC	0031226, 0005887, 0009986
Lmo2059	Voltage-gated potassium channel	msIMP, Unc-SP	Sec, YidC	0031226, 0005887, 0009986
Lmo2063	Protein of unknown function	msIMP, Unc-SP	Sec, YidC	0031226, 0005887, 0009986
Lmo2064	Large-conductance mechanosensitive channel, MscL	msIMP, Unc-SP	Sec, YidC	0031226, 0005887, 0009986
Lmo2065	Protein of unknown function	msIMP, Unc-SP	Sec, YidC	0031226, 0005887, 0009986
Lmo2066	Protein of unknown function	msIMP, Unc-SP	Sec, YidC	0031226, 0005887, 0009986
Lmo2071	Protein of unknown function	msIMP, Unc-SP	Sec, YidC	0031226, 0005887, 0009986
Lmo2082	Camphor resistance CrcB protein	msIMP, Unc-SP	Sec, YidC	0031226, 0005887, 0009986
Lmo2087	Multidrug efflux pump, VmrA	msIMP, Unc-SP	Sec, YidC	0031226, 0005887, 0009986
Lmo2115	Bacitracin exporter, BceAB (BarAB, YtsCD)	msIMP, Unc-SP	Sec, YidC	0031226, 0005887, 0009986
Lmo2116	Protein of unknown function	msIMP, Unc-SP	Sec, YidC	0031226, 0005887, 0009986
Lmo2123	ABC-type maltose transport systems, permease component	msIMP, Unc-SP	Sec, YidC	0031226, 0005887, 0009986
Lmo2124	ABC-type sugar transport systems, permease components	msIMP, Unc-SP	Sec, YidC	0031226, 0005887, 0009986
Lmo2127	Abortive infection protein	msIMP, Unc-SP	Sec, YidC	0031226, 0005887, 0009986
Lmo2129	Protein of unknown function	msIMP, Unc-SP	Sec, YidC	0031226, 0005887, 0009986
Lmo2130	Amino acid transporters	msIMP, Unc-SP	Sec, YidC	0031226, 0005887, 0009986

Lmo2145	Niacin/nicotinamide transporter, NiaY	msIMP, Unc-SP	Sec, YidC	0031226, 0005887, 0009986
Lmo2147	Sulfate exporter family, efflux pump, YeiH	msIMP, Unc-SP	Sec, YidC	0031226, 0005887, 0009986
Lmo2148	Protein of unknown function with DUF1361 domain and , COG4330	msIMP, Unc-SP	Sec, YidC	0031226, 0005887, 0009986
Lmo2150	Protein of unknown function	msIMP, Unc-SP	Sec, YidC	0031226, 0005887, 0009986
Lmo2169	Protein of unknown function	msIMP, Unc-SP	Sec, YidC	0031226, 0005887, 0009986
Lmo2177	Protein of unknown function	msIMP, Unc-SP	Sec, YidC	0031226, 0005887, 0009986
Lmo2194	ABC-type dipeptide/oligopeptide/nickel transport system, permease component	msIMP, Unc-SP	Sec, YidC	0031226, 0005887, 0009986
Lmo2195	ABC-type dipeptide/oligopeptide/nickel transport system, permease component	msIMP, Unc-SP	Sec, YidC	0031226, 0005887, 0009986
Lmo2197	Protein of unknown function	msIMP, Unc-SP	Sec, YidC	0031226, 0005887, 0009986
Lmo2204	Protein of unknown function	msIMP, Unc-SP	Sec, YidC	0031226, 0005887, 0009986
Lmo2207	Protein interacting with FtsH	msIMP, Unc-SP	Sec, YidC	0031226, 0005887, 0009986
Lmo2214	ABC-type transporter, EcsB	msIMP, Unc-SP	Sec, YidC	0031226, 0005887, 0009986
Lmo2218	Protein of unknown function	msIMP, Unc-SP	Sec, YidC	0031226, 0005887, 0009986
Lmo2226	Protein of unknown function	msIMP, Unc-SP	Sec, YidC	0031226, 0005887, 0009986
Lmo2228	Protein of unknown function	msIMP, Unc-SP	Sec, YidC	0031226, 0005887, 0009986
Lmo2231	Cation efflux protein	msIMP, Unc-SP	Sec, YidC	0031226, 0005887, 0009986
Lmo2232	Hemolysin, CBS domains	msIMP, Unc-SP	Sec, YidC	0031226, 0005887, 0009986
Lmo2237	MFS general substrate transporter	msIMP, Unc-SP	Sec, YidC	0031226, 0005887, 0009986
Lmo2238	MFS general substrate transporter	msIMP, Unc-SP	Sec, YidC	0031226, 0005887, 0009986
Lmo2239	Protein of unknown function	msIMP, Unc-SP	Sec, YidC	0031226, 0005887, 0009986
Lmo2254	Xanthine/uracil/vitamin C permease	msIMP, Unc-SP	Sec, YidC	0031226, 0005887, 0009986
Lmo2265	Protein of unknown function with DUF1516 domain	msIMP, Unc-SP	Sec, YidC	0031226, 0005887, 0009986
Lmo2330	Protein of unknown function	msIMP, Unc-SP	Sec, YidC	0031226, 0005887, 0009986
Lmo2348	ABC-type amino acid transport system, permease component	msIMP, Unc-SP	Sec, YidC	0031226, 0005887, 0009986
Lmo2353	NhaP-type Na+/H+ and K+/H+ antiporters	msIMP, Unc-SP	Sec, YidC	0031226, 0005887, 0009986
Lmo2355	MFS general substrate transporter	msIMP, Unc-SP	Sec, YidC	0031226, 0005887, 0009986
Lmo2357	Acid-resistance membrane protein, COG3247	msIMP, Unc-SP	Sec, YidC	0031226, 0005887, 0009986
Lmo2371	ABC-type transport system, involved in lipoprotein release, permease component	msIMP, Unc-SP	Sec, YidC	0031226, 0005887, 0009986
Lmo2377	MFS general substrate transporter	msIMP, Unc-SP	Sec, YidC	0031226, 0005887, 0009986
Lmo2382	Monovalent cation/H+ antiporter subunit E	msIMP, Unc-SP	Sec, YidC	0031226, 0005887, 0009986
Lmo2384	Monovalent cation/H+ antiporter subunitGF	msIMP, Unc-SP	Sec, YidC	0031226, 0005887, 0009986
Lmo2387	Chloride channel, core	msIMP, Unc-SP	Sec, YidC	0031226, 0005887, 0009986

Lmo2399	Hemolysins and related proteins containing CBS domains	msIMP, Unc-SP	Sec, YidC	0031226, 0005887, 0009986
Lmo2404	4-toluene sulfonate uptake permease family	msIMP, Unc-SP	Sec, YidC	0031226, 0005887, 0009986
Lmo2405	Protein of unknown function with DUF1634 domain and , COG4272	msIMP, Unc-SP	Sec, YidC	0031226, 0005887, 0009986
Lmo2409	Protein of unknown function	msIMP, Unc-SP	Sec, YidC	0031226, 0005887, 0009986
Lmo2418	ABC-type metal ion transport system, permease component	msIMP, Unc-SP	Sec, YidC	0031226, 0005887, 0009986
Lmo2421	Signal transduction histidine kinase	msIMP, Unc-SP	Sec, YidC	0031226, 0005887, 0009986
Lmo2423	Cation efflux protein	msIMP, Unc-SP	Sec, YidC	0031226, 0005887, 0009986
Lmo2427	Rod shape-determining protein RodA	msIMP, Unc-SP	Sec, YidC	0031226, 0005887, 0009986
Lmo2428	Rod shape-determining protein RodA	msIMP, Unc-SP	Sec, YidC	0031226, 0005887, 0009986
Lmo2430	Transport system permease protein	msIMP, Unc-SP	Sec, YidC	0031226, 0005887, 0009986
Lmo2435	Protein of unknown function, COG5658	msIMP, Unc-SP	Sec, YidC	0031226, 0005887, 0009986
Lmo2463	Drug exporters of the RND superfamily	msIMP, Unc-SP	Sec, YidC	0031226, 0005887, 0009986
Lmo2466	Protein of unknown function , COG4269	msIMP, Unc-SP	Sec, YidC	0031226, 0005887, 0009986
Lmo2469	Amino acid/polyamine transporter I	msIMP, Unc-SP	Sec, YidC	0031226, 0005887, 0009986
Lmo2484	Protein of unknown function , COG1950	msIMP, Unc-SP	Sec, YidC	0031226, 0005887, 0009986
Lmo2492	Protein of unknown function	msIMP, Unc-SP	Sec, YidC	0031226, 0005887, 0009986
Lmo2497	Phosphate transport system permease protein 2	msIMP, Unc-SP	Sec, YidC	0031226, 0005887, 0009986
Lmo2498	Phosphate ABC transporter, permease protein PstC	msIMP, Unc-SP	Sec, YidC	0031226, 0005887, 0009986
Lmo2500	Signal transduction histidine kinase, PhoR	msIMP, Unc-SP	Sec, YidC	0031226, 0005887, 0009986
Lmo2503	Cardiolipin synthetase	msIMP, Unc-SP	Sec, YidC	0031226, 0005887, 0009986
Lmo2506	Cell division protein, FtsX	msIMP, Unc-SP	Sec, YidC	0031226, 0005887, 0009986
Lmo2508	Protein of unknown function with DUF161 domain and , COG1284	msIMP, Unc-SP	Sec, YidC	0031226, 0005887, 0009986
Lmo2527	Protein of unknown function with unlceaved signal peptide, YwzB-like, COG4836	msIMP, Unc-SP	Sec, YidC	0031226, 0005887, 0009986
Lmo2535	F0F1 ATP synthase, subunit A	msIMP, Unc-SP	Sec, YidC	0031226, 0005887, 0009986
Lmo2536	F0F1 ATP synthase, subunit I	msIMP, Unc-SP	Sec, YidC	0031226, 0005887, 0009986
Lmo2549	Wall teichoic acid glycosylation protein, GtcA	msIMP, Unc-SP	Sec, YidC	0031226, 0005887, 0009986
Lmo2553	Protein of unknown function , CHP00374-like, COG0392	msIMP, Unc-SP	Sec, YidC	0031226, 0005887, 0009986
Lmo2563	Peptidase M50	msIMP, Unc-SP	Sec, YidC	0031226, 0005887, 0009986
Lmo2567	Protein of unknown function	msIMP, Unc-SP	Sec, YidC	0031226, 0005887, 0009986
Lmo2570	Peptide-antibiotic killer factor immunity protein, SdpI family, SdpC (YvaZ)	msIMP, Unc-SP	Sec, YidC	0031226, 0005887, 0009986
Lmo2575	Cation efflux protein	msIMP, Unc-SP	Sec, YidC	0031226, 0005887, 0009986
Lmo2581	ABC-type antimicrobial peptide transport system, permease component	msIMP, Unc-SP	Sec, YidC	0031226, 0005887, 0009986

Lmo2582	Signal transduction histidine kinase	msIMP, Unc-SP	Sec, YidC	0031226, 0005887, 0009986
Lmo2588	Drug resistance transporter EmrB/QacA subfamily	msIMP, Unc-SP	Sec, YidC	0031226, 0005887, 0009986
Lmo2599	Cobalt transport protein	msIMP, Unc-SP	Sec, YidC	0031226, 0005887, 0009986
Lmo2634	Cobalt transport protein	msIMP, Unc-SP	Sec, YidC	0031226, 0005887, 0009986
Lmo2635	1,4-dihydroxy-2-naphthoate octaprenyltransferase	msIMP, Unc-SP	Sec, YidC	0031226, 0005887, 0009986
Lmo2681	K+ transporting ATPase, B subunit, KdpB	msIMP, Unc-SP	Sec, YidC	0031226, 0005887, 0009986
Lmo2684	Phosphotransferase system, lactose/cellobiose IIC component	msIMP, Unc-SP	Sec, YidC	0031226, 0005887, 0009986
Lmo2689	Cation transport ATPase	msIMP, Unc-SP	Sec, YidC	0031226, 0005887, 0009986
Lmo2708	Phosphotransferase system, lactose/cellobiose IIC component	msIMP, Unc-SP	Sec, YidC	0031226, 0005887, 0009986
Lmo2715	ABC transporter, cysteine exporter family, permease/ATP-binding protein, CydD	msIMP, Unc-SP	Sec, YidC	0031226, 0005887, 0009986
Lmo2716	ABC transporter, cysteine exporter family, permease/ATP-binding protein, CydC	msIMP, Unc-SP	Sec, YidC	0031226, 0005887, 0009986
Lmo2725	Na+-driven multidrug efflux pump	msIMP, Unc-SP	Sec, YidC	0031226, 0005887, 0009986
Lmo2738	Hemolysins and related proteins containing CBS domains	msIMP, Unc-SP	Sec, YidC	0031226, 0005887, 0009986
Lmo2741	Drug efflux system protein, MdtG	msIMP, Unc-SP	Sec, YidC	0031226, 0005887, 0009986
Lmo2751	ABC-type bacteriocin/lantibiotic exporters, ATP-binding/permease protein	msIMP, Unc-SP	Sec, YidC	0031226, 0005887, 0009986
Lmo2752	ABC-type bacteriocin/lantibiotic exporters, ATP-binding/permease protein	msIMP, Unc-SP	Sec, YidC	0031226, 0005887, 0009986
Lmo2763	Phosphotransferase system cellobiose-specific component IIC	msIMP, Unc-SP	Sec, YidC	0031226, 0005887, 0009986
Lmo2768	Protein of unknown function	msIMP, Unc-SP	Sec, YidC	0031226, 0005887, 0009986
Lmo2775	Bacteriocin-associated integral membrane protein	msIMP, Unc-SP	Sec, YidC	0031226, 0005887, 0009986
Lmo2777	MFS general substrate transporter	msIMP, Unc-SP	Sec, YidC	0031226, 0005887, 0009986
Lmo2783	Phosphotransferase system cellobiose-specific component IIC	msIMP, Unc-SP	Sec, YidC	0031226, 0005887, 0009986
Lmo2799	Phosphotransferase system, mannitol-specific IIBC component	msIMP, Unc-SP	Sec, YidC	0031226, 0005887, 0009986
Lmo2816	MFS general substrate transporter	msIMP, Unc-SP	Sec, YidC	0031226, 0005887, 0009986
Lmo2818	MFS general substrate transporter	msIMP, Unc-SP	Sec, YidC	0031226, 0005887, 0009986
Lmo2826	MFS general substrate transporter	msIMP, Unc-SP	Sec, YidC	0031226, 0005887, 0009986
Lmo2837	ABC-type sugar transport system, permease component	msIMP, Unc-SP	Sec, YidC	0031226, 0005887, 0009986
Lmo2838	ABC-type sugar transport systems, permease components	msIMP, Unc-SP	Sec, YidC	0031226, 0005887, 0009986
Lmo2850	MFS general substrate transporter	msIMP, Unc-SP	Sec, YidC	0031226, 0005887, 0009986
Lmo0029	Protein of unknown function	msIMP, SP	Sec, YidC, SPase I	0031226, 0005887, 0009986
Lmo0037	Amino acid/polyamine transporter I	msIMP, SP	Sec, YidC, SPase I	0031226, 0005887, 0009986
Lmo0057	WXG100 protein secretion system, membrane component, EsaA	msIMP, SP	Sec, YidC, SPase I	0031226, 0005887, 0009986
Lmo0136	ABC-type dipeptide/oligopeptide/nickel transport system, permease component	msIMP, SP	Sec, YidC, SPase I	0031226, 0005887, 0009986

Lmo0169	Sugar uptake permease	msIMP, SP	Sec, YidC, SPase I	0031226, 0005887, 0009986
Lmo0179	ABC-type sugar transport system, permease component	msIMP, SP	Sec, YidC, SPase I	0031226, 0005887, 0009986
Lmo0234	Protein with PIN domain superfamily, COG4956	msIMP, SP	Sec, YidC, SPase I	0031226, 0005887, 0009986
Lmo0365	High-affinity Fe2+/Pb2+ permease	msIMP, SP	Sec, YidC, SPase I	0031226, 0005887, 0009986
Lmo0404	Protein of unknown function	msIMP, SP	Sec, YidC, SPase I	0031226, 0005887, 0009986
Lmo0414	Divalent heavy-metal cations transporter	msIMP, SP	Sec, YidC, SPase I	0031226, 0005887, 0009986
Lmo0444	Phage infection protein, YhgE, C-terminal	msIMP, SP	Sec, YidC, SPase I	0031226, 0005887, 0009986
Lmo0448	Amino acid/polyamine transporter I	msIMP, SP	Sec, YidC, SPase I	0031226, 0005887, 0009986
Lmo0449	Protein of unknown function	msIMP, SP	Sec, YidC, SPase I	0031226, 0005887, 0009986
Lmo0576	Protein of unknown function with DUF1085 domain	msIMP, SP	Sec, YidC, SPase I	0031226, 0005887, 0009986
Lmo0591	Protein of unknown function with DUF1295 domain, COG3752	msIMP, SP	Sec, YidC, SPase I	0031226, 0005887, 0009986
Lmo0626	Protein of unknown function	msIMP, SP	Sec, YidC, SPase I	0031226, 0005887, 0009986
Lmo0632	Phosphotransferase system, fructose-specific IIC component	msIMP, SP	Sec, YidC, SPase I	0031226, 0005887, 0009986
Lmo0650	Phage infection protein, YhgE	msIMP, SP	Sec, YidC, SPase I	0031226, 0005887, 0009986
Lmo0666	Protein of unknown function with DUF423 domain, COG2363	msIMP, SP	Sec, YidC, SPase I	0031226, 0005887, 0009986
Lmo0676	Flagellar export apparatus, membrane subunit FliP	msIMP, SP	Sec, YidC, SPase I	0031226, 0005887, 0009986
Lmo0678	Flagellar export apparatus, membrane subunit FliR	msIMP, SP	Sec, YidC, SPase I	0031226, 0005887, 0009986
Lmo0767	ABC-type sugar transport system, permease component	msIMP, SP	Sec, YidC, SPase I	0031226, 0005887, 0009986
Lmo0831	Malonate transporter, MdcF	msIMP, SP	Sec, YidC, SPase I	0031226, 0005887, 0009986
Lmo0839	MFS general substrate transporter	msIMP, SP	Sec, YidC, SPase I	0031226, 0005887, 0009986
Lmo0847	ABC-type amino acid transport system, permease component	msIMP, SP	Sec, YidC, SPase I	0031226, 0005887, 0009986
Lmo0860	ABC-type sugar transport systems, permease components	msIMP, SP	Sec, YidC, SPase I	0031226, 0005887, 0009986
Lmo0872	Arabinose efflux permease	msIMP, SP	Sec, YidC, SPase I	0031226, 0005887, 0009986
Lmo0897	Sulphate anion transporter	msIMP, SP	Sec, YidC, SPase I	0031226, 0005887, 0009986
Lmo0921	Protein of unknown function with DUF1980 domain, COG3689	msIMP, SP	Sec, YidC, SPase I	0031226, 0005887, 0009986
Lmo0959	Glycosyl transferase, family 4	msIMP, SP	Sec, YidC, SPase I	0031226, 0005887, 0009986
Lmo0973	D-alanine esterification of lipoteichoic acid and wall teichoic acid protein, DltB	msIMP, SP	Sec, YidC, SPase I	0031226, 0005887, 0009986
Lmo0981	MFS general substrate transporter	msIMP, SP	Sec, YidC, SPase I	0031226, 0005887, 0009986
Lmo0991	Tellurium resistance membrane protein, TerC	msIMP, SP	Sec, YidC, SPase I	0031226, 0005887, 0009986
Lmo1004	Protein of unknown function with DUF218 domain, COG1434	msIMP, SP	Sec, YidC, SPase I	0031226, 0005887, 0009986
Lmo1040	ABC-type molybdate transport system, permease component	msIMP, SP	Sec, YidC, SPase I	0031226, 0005887, 0009986
Lmo1131	ABC-type transport system cysteine/glutathione, ATP-binding component	msIMP, SP	Sec, YidC, SPase I	0031226, 0005887, 0009986

Lmo1190	Dimethylbenzimidazole porter, CblT	msIMP, SP	Sec, YidC, SPase I	0031226, 0005887, 0009986
Lmo1204	Cobalamin (vitamin B12) biosynthesis CbiM	msIMP, SP	Sec, YidC, SPase I	0031226, 0005887, 0009986
Lmo1210	Orotate transporter, OroP	msIMP, SP	Sec, YidC, SPase I	0031226, 0005887, 0009986
Lmo1211	Orotate transporter, OroP	msIMP, SP	Sec, YidC, SPase I	0031226, 0005887, 0009986
Lmo1226	Drug exporters of the RND superfamily	msIMP, SP	Sec, YidC, SPase I	0031226, 0005887, 0009986
Lmo1252	Protein of unknown function with DUF161 domain, COG1284	msIMP, SP	Sec, YidC, SPase I	0031226, 0005887, 0009986
Lmo1300	Arsenical pump membrane protein	msIMP, SP	Sec, YidC, SPase I	0031226, 0005887, 0009986
Lmo1316	Phosphatidate cytidylyltransferase, CdsA	msIMP, SP	Sec, YidC, SPase I	0031226, 0005887, 0009986
Lmo1391	Deoxyribonucleoside permease, RnsD	msIMP, SP	Sec, YidC, SPase I	0031226, 0005887, 0009986
Lmo1416	Glycopeptide antibiotics resistance protein	msIMP, SP	Sec, YidC, SPase I	0031226, 0005887, 0009986
Lmo1527	Sec transcolon, bifunctional subunit SecDF	msIMP, SP	Sec, YidC, SPase I	0031226, 0005887, 0009986
Lmo1568	Protein of unknown function with DUF441 domain, COG2707	msIMP, SP	Sec, YidC, SPase I	0031226, 0005887, 0009986
Lmo1623	Phosphatidic acid phosphatase type 2/haloperoxidase	msIMP, SP	Sec, YidC, SPase I	0031226, 0005887, 0009986
Lmo1686	Protein of unknown function with DUF939 domain, COG4129	msIMP, SP	Sec, YidC, SPase I	0031226, 0005887, 0009986
Lmo1690	Protein of unknown function with DUF457 domain, COG1988	msIMP, SP	Sec, YidC, SPase I	0031226, 0005887, 0009986
Lmo1696	Glycopeptide antibiotics resistance protein	msIMP, SP	Sec, YidC, SPase I	0031226, 0005887, 0009986
Lmo1731	ABC-type sugar transport systems, permease components	msIMP, SP	Sec, YidC, SPase I	0031226, 0005887, 0009986
Lmo1945	Riboflavin uptake transporter, YpaA	msIMP, SP	Sec, YidC, SPase I	0031226, 0005887, 0009986
Lmo1958	ABC Fe3+-siderophore transport system, permease component, FhuB	msIMP, SP	Sec, YidC, SPase I	0031226, 0005887, 0009986
Lmo2001	Phosphotransferase system, mannose/fructose/N-acetylgalactosamine-specific IIC	msIMP, SP	Sec, YidC, SPase I	0031226, 0005887, 0009986
Lmo2009	ABC-type polysaccharide transport system, permease component	msIMP, SP	Sec, YidC, SPase I	0031226, 0005887, 0009986
Lmo2017	Phosphatidic acid phosphatase type 2/haloperoxidase	msIMP, SP	Sec, YidC, SPase I	0031226, 0005887, 0009986
Lmo2058	Cytochrome oxidase assembly protein, CtaA	msIMP, SP	Sec, YidC, SPase I	0031226, 0005887, 0009986
Lmo2062	Copper resistance D	msIMP, SP	Sec, YidC, SPase I	0031226, 0005887, 0009986
Lmo2070	Abortive infection protein	msIMP, SP	Sec, YidC, SPase I	0031226, 0005887, 0009986
Lmo2092	BCCT transporter	msIMP, SP	Sec, YidC, SPase I	0031226, 0005887, 0009986
Lmo2122	Maltodextrose utilization protein MalA	msIMP, SP	Sec, YidC, SPase I	0031226, 0005887, 0009986
Lmo2135	Phosphotransferase system, fructose-specific IIC component	msIMP, SP	Sec, YidC, SPase I	0031226, 0005887, 0009986
Lmo2140	ABC-type Na+ efflux pump, permease component	msIMP, SP	Sec, YidC, SPase I	0031226, 0005887, 0009986
Lmo2171	MFS general substrate transporter	msIMP, SP	Sec, YidC, SPase I	0031226, 0005887, 0009986
Lmo2183	Transport system permease protein	msIMP, SP	Sec, YidC, SPase I	0031226, 0005887, 0009986
Lmo2249	Phosphate transporter	msIMP, SP	Sec, YidC, SPase I	0031226, 0005887, 0009986

Lmo2250	ABC arginine/histidine transport system, permease component	msIMP, SP	Sec, YidC, SPase I	0031226, 0005887, 0009986
Lmo2255	Protein of unknown function	msIMP, SP	Sec, YidC, SPase I	0031226, 0005887, 0009986
Lmo2264	Protein of unknown function, GlnB-like, COG1284	msIMP, SP	Sec, YidC, SPase I	0031226, 0005887, 0009986
Lmo2279	Holin phage A118	msIMP, SP	Sec, YidC, SPase I	0031226, 0005887, 0009986
Lmo2360	Phage infection protein, YhgE	msIMP, SP	Sec, YidC, SPase I	0031226, 0005887, 0009986
Lmo2362	Glutamate:g-aminobutyrate antiporter	msIMP, SP	Sec, YidC, SPase I	0031226, 0005887, 0009986
Lmo2379	Multisubunit Na+/H+ antiporter, MnhB subunit	msIMP, SP	Sec, YidC, SPase I	0031226, 0005887, 0009986
Lmo2386	Acid phosphatase/vanadium-dependent haloperoxidase	msIMP, SP	Sec, YidC, SPase I	0031226, 0005887, 0009986
Lmo2443	Protein of unknown function	msIMP, SP	Sec, YidC, SPase I	0031226, 0005887, 0009986
Lmo2612	Sec translocon, subunit SecY	msIMP, SP	Sec, YidC, SPase I	0031226, 0005887, 0009986
Lmo2640	Heptaprenyl diphosphate synthase	msIMP, SP	Sec, YidC, SPase I	0031226, 0005887, 0009986
Lmo2688	Cell cycle protein	msIMP, SP	Sec, YidC, SPase I	0031226, 0005887, 0009986
Lmo2745	ABC-type bacteriocin/lantibiotic exporters, ATP-binding/permease protein	msIMP, SP	Sec, YidC, SPase I	0031226, 0005887, 0009986
Lmo2845	MFS general substrate transporter	msIMP, SP	Sec, YidC, SPase I	0031226, 0005887, 0009986
Lmo0013	AA3-600 quinol oxidase subunit II, QoxA	msIMP-Lipoprotein	Sec, YidC, Lgt, SPase II	0031226, 0005887, 0046658, 0009986
Lmo0269	ABC-type dipeptide/oligopeptide/nickel transport systems, permease component	msIMP-Lipoprotein	Sec, YidC, Lgt, SPase II	0031226, 0005887, 0046658, 0009986
Lmo0641	Heavy metal translocating P-type ATPase	msIMP-Lipoprotein	Sec, YidC, Lgt, SPase II	0031226, 0005887, 0046658, 0009986
Lmo1379	YidC insertase, OxaA-like protein, OxaA1 (YqjG)	msIMP-Lipoprotein	Sec, YidC, Lgt, SPase II	0031226, 0005887, 0046658, 0009986
Lmo2687	Cell division protein, FtsW	msIMP-Lipoprotein	Sec, YidC, Lgt, SPase II	0031226, 0005887, 0046658, 0009986
Lmo2854	YidC insertase, OxaA-like protein, OxaA2 (SpoIIIJ)	msIMP-Lipoprotein	Sec, YidC, Lgt, SPase II	0031226, 0005887, 0046658, 0009986

[a]Some annotations were corrected respective to the homology search performed as described in the Material & Methods section. More extensive and detailed annotations are available in Table 1S.

[b]Single-spanning IMP (ss-IMP) are categorized into (i) Type I, *i.e.* IMP exhibiting a cleavable SP in addition to another TMD positioned upstream, (i) Type II, *i.e.* IMP exhibiting a signal anchor, which is a TMD with N_{in}-C_{out} orientation (Type I module), that could be an uncleaved SP (Unc-SP), (iii) Type III, *i.e.* IMP exhibiting an reverse signal-anchor, which is a TMD with N_{out}-C_{in} orientation (Type II module).

[c]Subcellular location follow the GO (Gene Ontology) for cellular component. Besides location to intrinsic to the cytoplasmic membrane (GO:0031226), IMPs are more precisely integral to cytoplasmic membrane (GO:0005887) and consequently at cell surface (GO:0009986).
