# Supplementary material for: Subcellular Localization of Extracytoplasmic Proteins in Monoderm Bacteria: Rational Secretomics-Based Strategy for Genomic and Proteomic Analyses
Source: PLoS One. 2012 Aug 9;7(8):e42982. doi: 10.1371/journal.pone.0042982 (PMC3415414; doi:10.1371/journal.pone.0042982)
Supplement: Table S3 — Summarised information about protein categories, secretion pathways and GO terms for IMPs, lipoproteins, cell-wall proteins, subunits of supramolecular cell-surface appendages and exoproteins, respectively, as predicted by the secretomics-based method in L. monocytogenes EGD-e. (PDF) [file pone.0042982.s003.pdf]

Table 3S: **The 74 lipoproteins in *L. monocytogenes* EGD-e as revealed by the secretomics-based method.**

Protein ID	Annotation[a]	Protein category	Secretion pathway[b]	GO[c]
Lmo0047	Peptidase M4, PepSY	Lipoprotein	Sec, Lgt, SPase II	0031226, 0046658, 0009986
Lmo0135	ABC-type dipeptide transport system, substrate-binding protein family 5 component	Lipoprotein	Sec, Lgt, SPase II	0031226, 0046658, 0009986,0005576
Lmo0152	ABC-type oligopeptide transport system, substrate-binding protein family 5 component	Lipoprotein	Sec, Lgt, SPase II	0031226, 0046658, 0009986,0005576
Lmo0153	ABC-type metal ion transport system, substrate-binding protein component, surface adhesin	Lipoprotein	Sec, Lgt, SPase II	0031226, 0046658, 0009986,0005576
Lmo0181	ABC-type sugar transport system, substrate-binding protein family 1 component	Lipoprotein	Sec, Lgt, SPase II	0031226, 0046658, 0009986,0005576
Lmo0207	Lipoprotein of unknown function with DUF1307 domain, YehR-like, COG4808	Lipoprotein	Sec, Lgt, SPase II	0031226, 0046658, 0009986,0005576
Lmo0255	Lipoprotein of unknown function with DUF1307 domain, YehR-like, COG4808	Lipoprotein	Sec, Lgt, SPase II	0031226, 0046658, 0009986,0005576
Lmo0285	ABC-type metal ion transport system, substrate-binding protein component, surface antigen	Lipoprotein	Sec, Lgt, SPase II	0031226, 0046658, 0009986,0005576
Lmo0303	Protein of unknown function, lysin rich	Lipoprotein	Sec, Lgt, SPase II	0031226, 0046658, 0009986,0005576
Lmo0324	Protein of unknown function	Lipoprotein	Sec, Lgt, SPase II	0031226, 0046658, 0009986
Lmo0355	Fumarate reductase flavoprotein subunit	Lipoprotein	Sec, Lgt, SPase II	0031226, 0046658, 0009986,0005576
Lmo0366	Iron/lead transport substrate-binding protein, YcdO-like, PbrT-like	Lipoprotein	Sec, Lgt, SPase II	0031226, 0046658, 0009986
Lmo0460	Adhesin with RGD motif, LRR (Leucine-rich repeat) and DUF285 domain, RNI-like	Lipoprotein	Sec, Lgt, SPase II	0031226, 0046658, 0009986,0005576
Lmo0510	Protein of unknown function	Lipoprotein	Sec, Lgt, SPase II	0031226, 0046658, 0009986
Lmo0517	Phosphoglycerate mutase	Lipoprotein	Sec, Lgt, SPase II	0031226, 0046658, 0009986,0005576
Lmo0541	ABC-type Fe^{3+}-hydroxamate transport system, substrate-binding protein component	Lipoprotein	Sec, Lgt, SPase II	0031226, 0046658, 0009986,0005576
Lmo0617	Protein of unknown function	Lipoprotein	Sec, Lgt, SPase II	0031226, 0046658, 0009986,0005576
Lmo0768	ABC-type sugar transport system, substrate-binding protein family 1 component	Lipoprotein	Sec, Lgt, SPase II	0031226, 0046658, 0009986,0005576
Lmo0791	Protein of unknown function, YcdA-like spore lipoprotein	Lipoprotein	Sec, Lgt, SPase II	0031226, 0046658, 0009986,0005576
Lmo0859	ABC-type trehalose/maltose transport system, substrate-binding protein component	Lipoprotein	Sec, Lgt, SPase II	0031226, 0046658, 0009986,0005576
Lmo0945	Metallo-β-lactamase family protein, DNA-binding ComEC/Rec2-like protein	Lipoprotein	Sec, Lgt, SPase II	0031226, 0046658, 0009986
Lmo0953	Protein of unknown function	Lipoprotein	Sec, Lgt, SPase II	0031226, 0046658, 0009986
Lmo1016	ABC-type proline/glycine betaine transport system, substrate-binding protein component	Lipoprotein	Sec, Lgt, SPase II	0031226, 0046658, 0009986,0005576
Lmo1041	ABC-type molybdate transport system, substrate-binding protein component	Lipoprotein	Sec, Lgt, SPase II	0031226, 0046658, 0009986
Lmo1068	Protein of unknown function	Lipoprotein	Sec, Lgt, SPase II	0031226, 0046658, 0009986,0005576
Lmo1073	ABC-type Fe^{3+}-hydroxamate transport system, substrate-binding protein component	Lipoprotein	Sec, Lgt, SPase II	0031226, 0046658, 0009986,0005576
Lmo1265	Protein of unknown function	Lipoprotein	Sec, Lgt, SPase II	0031226, 0046658, 0009986

Lmo1340	Protein of unknown function, YqgU-like	Lipoprotein	Sec, Lgt, SPase II	0031226, 0046658, 0009986
Lmo1388	CD4+ T-cell stimulating antigen, TcsA, substrate-binding protein ABC transport system	Lipoprotein	Sec, Lgt, SPase II	0031226, 0046658, 0009986,0005576
Lmo1426	ABC-type betaine/choline transport system, osmoprotectant, OpuCC	Lipoprotein	Sec, Lgt, SPase II	0031226, 0046658, 0009986
Lmo1444	Peptidylprolyl isomerase, foldase, PrsA1	Lipoprotein	Sec, Lgt, SPase II	0031226, 0046658, 0009986,0005576
Lmo1649	Protein of unknown function	Lipoprotein	Sec, Lgt, SPase II	0031226, 0046658, 0009986
Lmo1653	Protein of unknown function	Lipoprotein	Sec, Lgt, SPase II	0031226, 0046658, 0009986
Lmo1671	ABC-type metal ion transport system, substrate-binding protein component, surface adhesin	Lipoprotein	Sec, Lgt, SPase II	0031226, 0046658, 0009986
Lmo1730	ABC-type sugar transport system, substrate-binding protein component	Lipoprotein	Sec, Lgt, SPase II	0031226, 0046658, 0009986,0005576
Lmo1738	ABC-type amino acid transport/signal transduction, surface adhesin	Lipoprotein	Sec, Lgt, SPase II	0031226, 0046658, 0009986
Lmo1757	Sex pheromone cAM373 biosynthesis, CamS	Lipoprotein	Sec, Lgt, SPase II	0031226, 0046658, 0009986
Lmo1800	Receptor-linked protein tyrosine/serine phosphatase	Lipoprotein	Sec, Lgt, SPase II	0031226, 0046658, 0009986,0005576
Lmo1847	ABC transport, metal-binding protein, surface adhesin, lipoprotein promoting entry, LpeA	Lipoprotein	Sec, Lgt, SPase II	0031226, 0046658, 0009986
Lmo1903	Thioredoxin, Bacteriocin transport accessory protein	Lipoprotein	Sec, Lgt, SPase II	0031226, 0046658, 0009986
Lmo1959	ABC-type Fe^{3+}-hydroxamate transport system, substrate-binding protein component	Lipoprotein	Sec, Lgt, SPase II	0031226, 0046658, 0009986,0005576
Lmo2007	ABC-type sugar transport system, substrate-binding protein component	Lipoprotein	Sec, Lgt, SPase II	0031226, 0046658, 0009986,0005576
Lmo2023	L-aspartate oxidase, NadB	Lipoprotein	Sec, Lgt, SPase II	0031226, 0046658, 0009986
Lmo2079	Protein of unknown function	Lipoprotein	Sec, Lgt, SPase II	0031226, 0046658, 0009986,0005576
Lmo2080	Protein of unknown function	Lipoprotein	Sec, Lgt, SPase II	0031226, 0046658, 0009986
Lmo2125	ABC-type maltose transporter, substrate-binding protein family 1 component	Lipoprotein	Sec, Lgt, SPase II	0031226, 0046658, 0009986,0005576
Lmo2184	ABC-type Fe^{3+}-hydroxamate transport system, substrate-binding protein component	Lipoprotein	Sec, Lgt, SPase II	0031226, 0046658, 0009986,0005576
Lmo2196	ABC-type oligopeptide transport system, substrate-binding protein family 5 component	Lipoprotein	Sec, Lgt, SPase II	0031226, 0046658, 0009986,0005576
Lmo2219	Peptidylprolyl isomerase, foldase, PrsA2	Lipoprotein	Sec, Lgt, SPase II	0031226, 0046658, 0009986,0005576
Lmo2331	Protein of unknown function	Lipoprotein	Sec, Lgt, SPase II	0031226, 0046658, 0009986,0005576
Lmo2349	ABC-type amino acid transport/signal transduction, surface adhesin	Lipoprotein	Sec, Lgt, SPase II	0031226, 0046658, 0009986,0005576
Lmo2416	Protein of unknown function	Lipoprotein	Sec, Lgt, SPase II	0031226, 0046658, 0009986
Lmo2417	ABC-type metal ion transport system, substrate-binding protein component, surface antigen	Lipoprotein	Sec, Lgt, SPase II	0031226, 0046658, 0009986,0005576
Lmo2431	ABC-type Fe^{3+}-hydroxamate transport system, substrate-binding protein component	Lipoprotein	Sec, Lgt, SPase II	0031226, 0046658, 0009986,0005576
Lmo2446	Glycoside hydrolase, family 31	Lipoprotein	Sec, Lgt, SPase II	0031226, 0046658, 0009986
Lmo2499	ABC-type phosphate transport system, substrate-binding protein component	Lipoprotein	Sec, Lgt, SPase II	0031226, 0046658, 0009986,0005576
Lmo2569	ABC-type oligopeptide transport system, substrate-binding protein component	Lipoprotein	Sec, Lgt, SPase II	0031226, 0046658, 0009986
Lmo2578	α/β hydrolase with a lipobox motif	Lipoprotein	Sec, Lgt, SPase II	0031226, 0046658, 0009986

Lmo2594	Protein of unknown function	Lipoprotein	Sec, Lgt, SPase II	0031226, 0046658, 0009986
Lmo2595	Protein of unknown function	Lipoprotein	Sec, Lgt, SPase II	0031226, 0046658, 0009986,0005576
Lmo2636	Thiamine biosynthesis lipoprotein ApbE-like	Lipoprotein	Sec, Lgt, SPase II	0031226, 0046658, 0009986,0005576
Lmo2637	Major membrane immunogen, electron transport complex RnfABCDGE type	Lipoprotein	Sec, Lgt, SPase II	0031226, 0046658, 0009986,0005576
Lmo2642	Phosphoesterase	Lipoprotein	Sec, Lgt, SPase II	0031226, 0046658, 0009986
Lmo2812	D-alanyl-D-alanine carboxypeptidase	Lipoprotein	Sec, Lgt, SPase II	0031226, 0046658, 0009986
Lmo2839	ABC-type sugar transport system, substrate-binding protein component	Lipoprotein	Sec, Lgt, SPase II	0031226, 0046658, 0009986,0005576
Lmo1136	Protein of unknown function with LRR (Leucine-rich repeat), internalin-like	LPXTG-Lipoprotein	Sec, Lgt, SPase II,SrtA	0031226, 0046658, 0009275, 0009986
Lmo0013	AA3-600 quinol oxidase subunit II, QoxA	msIMP-Lipoprotein	Sec, YidC, Lgt, SPase II	0031226, 0005887, 0046658, 0009986,0005576
Lmo0269	ABC-type dipeptide/oligopeptide/nickel transport systems, permease component	msIMP-Lipoprotein	Sec, YidC, Lgt, SPase II	0031226, 0005887, 0046658, 0009986
Lmo0641	Heavy metal translocating P-type ATPase	msIMP-Lipoprotein	Sec, YidC, Lgt, SPase II	0031226, 0005887, 0046658, 0009986
Lmo0821	Protein of unknown function	ssIMP I-Lipoprotein	Sec, YidC, Lgt, SPase II	0031226, 0005887, 0046658, 0009986
Lmo1379	YidC insertase, OxaA-like protein, OxaA1 (YqjG)	msIMP-Lipoprotein	Sec, YidC, Lgt, SPase II	0031226, 0005887, 0046658, 0009986
Lmo2687	Bacterial cell division protein, FtsW	msIMP-Lipoprotein	Sec, YidC, Lgt, SPase II	0031226, 0005887, 0046658, 0009986
Lmo2793	Protein of unknown function	ssIMP I-Lipoprotein	Sec, YidC, Lgt, SPase II	0031226, 0005887, 0046658, 0009986
Lmo2854	YidC insertase, OxaA-like protein, OxaA2 (SpoIIIJ)	msIMP-Lipoprotein	Sec, YidC, Lgt, SPase II	0031226, 0005887, 0046658, 0009986,0005576

[a]Some annotations were corrected respective to the similarity search performed as described in the Material & Methods section. More extensive and detailed annotations are available in Table 1S.

[b]Following secretion *via* the Sec translocon, protein exhibiting a signal peptide of Type II (SP II) are taken in charge by the lipoprotein maturation pathway consisting of the prolipoprotein diacylglyceryltransferase (Lgt) and signal peptidases of Type II (SPase II) in *L. monocytogenes* EGD-e (Table 1).

[c]Subcellular location follow the GO (Gene Ontology) for cellular component. Besides location to intrinsic to the cytoplasmic membrane (GO:0031226), lipoproteins are more precisely anchored to cytoplasmic membrane (GO:0046658) and consequently at cell surface (GO:0009986). In some cases, the release of lipoproteins in the extracellular milieu (GO:0005876) can be expected by the presence of a glycine residue at position +2 of the SP cleavage site.
