# Supplementary material for: Subcellular Localization of Extracytoplasmic Proteins in Monoderm Bacteria: Rational Secretomics-Based Strategy for Genomic and Proteomic Analyses
Source: PLoS One. 2012 Aug 9;7(8):e42982. doi: 10.1371/journal.pone.0042982 (PMC3415414; doi:10.1371/journal.pone.0042982)
Supplement: Table S4 — Summarised information about protein categories, secretion pathways and GO terms for IMPs, lipoproteins, cell-wall proteins, subunits of supramolecular cell-surface appendages and exoproteins, respectively, as predicted by the secretomics-based method in L. monocytogenes EGD-e. (PDF) [file pone.0042982.s004.pdf]

Table 4S: **The 58 secreted proteins anchored to the cell wall in *L. monocytogenes* EGD-e as revealed by the secretomics-based method.**

Protein ID	Annotation[a]	Protein category	Secretion pathway[b]	GO[c]
Covalent anchoring				
Lmo0130	Bifunctional 2',3'-cyclic nucleotide 2'-phosphodiesterase/3'-nucleotidase, CpdB	LPXTG-protein	Sec, SPase I, SrtA	0009275, 0009986
Lmo0159	Collagen-binding protein	LPXTG-protein	Sec, SPase I, SrtA	0009275, 0009986
Lmo0160	Collagen-binding protein	LPXTG-protein	Sec, SPase I, SrtA	0009275, 0009986
Lmo0171	Protein of unknown function with LRR (Leucine-rich repeat), PKD, Ig-like, internalin-like	LPXTG-protein	Sec, SPase I, SrtA	0009275, 0009986
Lmo0175	Protein of unknown function with PKD, DUF1085 motif	LPXTG-protein	Sec, SPase I, SrtA	0009275, 0009986
Lmo0262	Internalin G, InlG, with LRR (Leucine-rich repeat)	LPXTG-protein	Sec, SPase I, SrtA	0009275, 0009986
Lmo0263	Internalin H, InlH, with LRR (Leucine-rich repeat)	LPXTG-protein	Sec, SPase I, SrtA	0009275, 0009986
Lmo0264	Internalin E, InlE, with LRR (Leucine-rich repeat)	LPXTG-protein	Sec, SPase I, SrtA	0009275, 0009986
Lmo0320	Virulence protein with LPXTG motif, Vip, recognition of Gp96 receptor	LPXTG-protein	Sec, SPase I, SrtA	0009275, 0009986
Lmo0327	Protein of unknown function with LRR (Leucine-rich repeat), DUF1085, internalin-like	LPXTG-protein	Sec, SPase I, SrtA	0009275, 0009986
Lmo0331	Protein of unknown function with LRR (Leucine-rich repeat), PKD motif, internalin-like	LPXTG-protein	Sec, SPase I, SrtA	0009275, 0009986
Lmo0333	Internalin I, InlI, with LRR (Leucine-rich repeat), PKD motif	LPXTG-protein	Sec, SPase I, SrtA	0009275, 0009986
Lmo0409	Internalin F, InlF, with LRR (Leucine-rich repeat)	LPXTG-protein	Sec, SPase I, SrtA	0009275, 0009986
Lmo0433	Internalin A, InlA, with LRR (Leucine-rich repeat)	LPXTG-protein	Sec, SPase I, SrtA	0009275, 0009986
Lmo0435	Biofilm-associated protein, BapL	LPXTG-protein	Sec, SPase I, SrtA	0009275, 0009986
Lmo0463	Protein of unknown function	LPXTG-protein	Sec, SPase I, SrtA	0009275, 0009986
Lmo0514	Protein of unknown function with LRR (Leucine-rich repeat), PKD motif, internalin-like	LPXTG-protein	Sec, SPase I, SrtA	0009275, 0009986
Lmo0550	Protein of unknown function with LPXTG motif, CscD-like	LPXTG-protein	Sec, SPase I, SrtA	0009275, 0009986
Lmo0610	Protein of unknown function with LRR (Leucine-rich repeat), PKD motif, internalin-like	LPXTG-protein	Sec, SPase I, SrtA	0009275, 0009986
Lmo0627	Collagen-binding protein	LPXTG-protein	Sec, SPase I, SrtA	0009275, 0009986
Lmo0725	Protein of unknown function	LPXTG-protein	Sec, SPase I, SrtA	0009275, 0009986
Lmo0732	Protein of unknown function with LRR, DUF1085, bacterial Ig-like, internalin-like	LPXTG-protein	Sec, SPase I, SrtA	0009275, 0009986
Lmo0801	Protein of unknown function with LRR (Leucine-rich repeat), PKD motif, internalin-like	LPXTG-protein	Sec, SPase I, SrtA	0009275, 0009986
Lmo0835	Protein of unknown function	LPXTG-protein	Sec, SPase I, SrtA	0009275, 0009986
Lmo0842	Invasin/intimin cell-adhesion protein with bacterial Ig-like domain	LPXTG-protein	Sec, SPase I, SrtA	0009275, 0009986
Lmo1115	Collagen-binding protein	LPXTG-protein	Sec, SPase I, SrtA	0009275, 0009986

Lmo1289	Protein of unknown function with LRR (Leucine-rich repeat), PKD motif, internalin-like	LPXTG-protein	Sec, SPase I, SrtA	0009275, 0009986
Lmo1290	Internalin K, InlK, Recruitment of the Major Vault Protein, with PKD motif	LPXTG-protein	Sec, SPase I, SrtA	0009275, 0009986
Lmo1413	Protein of unknown function with DUF1085 domain	LPXTG-protein	Sec, SPase I, SrtA	0009275, 0009986
Lmo1666	Bacterial adhesin with hyalin, cadherin domains, PKD repeats and RGD motif	LPXTG-protein	Sec, SPase I, SrtA	0009275, 0009986
Lmo1799	Protein of unkonwn function with Ig fold, transmembrane, ATPase	LPXTG-protein	Sec, SPase I, SrtA	0009275, 0009986
Lmo2026	Protein of unknown function with LRR, DUF1085, bacterial Ig-like, internalin-like	LPXTG-protein	Sec, SPase I, SrtA	0009275, 0009986
Lmo2085	Collagen-binding protein	LPXTG-protein	Sec, SPase I, SrtA	0009275, 0009986
Lmo2178	Collagen-binding protein	LPXTG-protein	Sec, SPase I, SrtA	0009275, 0009986
Lmo2179	Protein of unknown function with DUF1085 domain, Cna-like	LPXTG-protein	Sec, SPase I, SrtA	0009275, 0009986
Lmo2185	Surface virulence-associated protein, SvpA (P64), NEAr transporter	LPXTG-protein	Sec, SPase I, SrtB	0009275, 0009986
Lmo2186	Surface virulence-associated protein, SvpB, NEAr transporter	LPXTG-protein	Sec, SPase I, SrtB	0009275, 0009986
Lmo2396	Protein of unknown function with LRR (Leucine-rich repeat), DUF1085, internalin-like	LPXTG-protein	Sec, SPase I, SrtA	0009275, 0009986
Lmo2576	Collagen-binding protein	LPXTG-protein	Sec, SPase I, SrtA	0009275, 0009986
Lmo2714	Protein of unknown function	LPXTG-protein	Sec, SPase I, SrtA	0009275, 0009986
Lmo2821	Internalin J, InlJ, with LRR (Leucine-rich repeat)	LPXTG-protein	Sec, SPase I, SrtA	0009275, 0009986
Lmo0880	Collagen-binding protein with peptidoglycan-binding LysMs	LPXTG-LysM-protein	Sec, SPase I, SrtA	0009275, 0009986
Lmo1136	Protein of unknown function with LRR (Leucine-rich repeat), internalin-like	LPXTG-Lipoprotein	Sec, Lgt, SPase II, SrtA	0031226, 0046658, 0009275, 0009986
Non-covalent anchoring				
Lmo0582	Cell-wall hydrolase, protein of 60 KDa (P60), invasion associated protein, Iap	LysM-protein	Sec, SPase I	0009275, 0009986
Lmo2522	Protein of unknown function	LysM-protein	Sec, SPase I	0009275, 0009986
Lmo2691	Autolysin, N-acetylmuramoyl-L-alanine amidase, MurA	LysM-protein	Sec, SPase I	0009275, 0009986
Lmo1303	Cell division suppressor protein, YneA	ssIMP II-LysM-protein	Sec, YidC	0031226, 0005887, 0009275, 0009986
Lmo1941	Protein of unknown function, YpbE-like, ATPase	ssIMP II-LysM-protein	Sec, YidC	0031226, 0005887, 0009275, 0009986
Lmo0549	Protein of unknown function with LRR (Leucine-rich repeat), CscB-like	WXL-protein	Sec, SPase I	0009275, 0009986
Lmo0551	Protein of unknown function, CscB-like	WXL-protein	Sec, SPase I	0009275, 0009986
Lmo0585	Protein of unknown function, CscB-like	WXL-protein	Sec, SPase I	0009275, 0009986
Lmo0587	Protein of unknown function, CscB-like	WXL-protein	Sec, SPase I	0009275, 0009986
Lmo0434	Internalin B, InlB, with LRR (Leucine-rich repeat)	GW-protein	Sec, SPase I	0009275, 0009986
Lmo1076	N-acetylmuramoyl-L-alanine amidase, Auto	GW-protein	Sec, SPase I	0009275, 0009986
Lmo2203	N-acetylmuramoyl-L-alanine amidase	GW-protein	Sec, SPase I	0009275, 0009986
Lmo2558	N-acetylmuramoyl-L-alanine amidase family 2, autolysin, Ami	GW-protein	Sec, SPase I	0009275, 0009986

Lmo2591	N-acetylmuramoyl-L-alanine amidase	GW-protein	Sec, SPase I	0009275, 0009986
Lmo1851	Peptidase S41	PGBD1-protein	Sec, SPase I	0009275, 0009986

[a]Some annotations were corrected respective to the similarity search performed as described in the Material & Methods section. More extensive and detailed annotations are available in Table 1S.

[b]Following secretion *via* the Sec translocon, N-terminal signal peptide of Type I (SP I) is cleaved by signal peptidases of Type I (SPase I) whereas C-terminal LPXTG domain is cleaved and covalently linked to peptidoglycan precursor by sortase A (SrtA) or SrtB in *L. monocytogenes* EGD-e (Table 1).

[c]Subcellular location follow the GO (Gene Ontology) for cellular component. LPXTG-, LysM-, WXL-, GW- and PGBD1-proteins are anchored to the cell wall (GO:0009275) and consequently at cell surface (GO:0009986).
