# Supplementary material for: Subcellular Localization of Extracytoplasmic Proteins in Monoderm Bacteria: Rational Secretomics-Based Strategy for Genomic and Proteomic Analyses
Source: PLoS One. 2012 Aug 9;7(8):e42982. doi: 10.1371/journal.pone.0042982 (PMC3415414; doi:10.1371/journal.pone.0042982)
Supplement: Table S5 — Summarised information about protein categories, secretion pathways and GO terms for IMPs, lipoproteins, cell-wall proteins, subunits of supramolecular cell-surface appendages and exoproteins, respectively, as predicted by the secretomics-based method in L. monocytogenes EGD-e. (PDF) [file pone.0042982.s005.pdf]

Table 5S: **The 16 secreted proteins part of supramolecular cell-surface appendage in *L. monocytogenes* EGD-e as revealed by the secretomics-based method.**

Protein ID	Annotation[a]	Protein Type	Secretion pathway[b]	GO[c]
Pseudo-pilus				
Lmo1341	Type 4 pseudo-prepilin, ComGG	FPE-substrate	FPE, ComC	0043234, 0009986
Lmo1342	Type 4 pseudo-prepilin, ComGF	FPE-substrate	FPE, ComC	0043234, 0009986
Lmo1343	Type 4 pseudo-prepilin, ComGE	FPE-substrate	FPE, ComC	0043234, 0009986
Lmo1344	Type 4 pseudo-prepilin, ComGD	FPE-substrate	FPE, ComC	0043234, 0009986
Lmo1345	Type 4 pseudo-prepilin, ComGC	FPE-substrate	FPE, ComC	0043234, 0009986
Flagellum				
Lmo0682	Flagellar basal body rod protein, FlgG	FEA-substrate	FEA	0043234, 0009986, 00019861
Lmo0690	Flagellin, FlaA	FEA-substrate	FEA	0043234, 0009986, 00019861
Lmo0695	Flagellar hook protein, FliK	FEA-substrate	FEA	0043234, 0009986, 00019861
Lmo0696	Flagellar hook capping protein, FlgD	FEA-substrate	FEA	0043234, 0009986, 00019861
Lmo0697	Flagellar hook protein, FlgE	FEA-substrate	FEA	0043234, 0009986, 00019861
Lmo0705	Flagellar hook-associated, FlgK	FEA-substrate	FEA	0043234, 0009986, 00019861
Lmo0706	Flagellar hook-associated, FlgL	FEA-substrate	FEA	0043234, 0009986, 00019861
Lmo0707	Flagellar capping protein, FliD	FEA-substrate	FEA	0043234, 0009986, 00019861
Lmo0710	Flagellar basal body rod protein, FlgB	FEA-substrate	FEA	0043234, 0009986, 00019861
Lmo0711	Flagellar basal body rod protein, FlgC	FEA-substrate	FEA	0043234, 0009986, 00019861
Lmo0712	Flagellar hook-basal body protein, FliE	FEA-substrate	FEA	0043234, 0009986, 00019861

[a]Some annotations were corrected respective to the similarity search performed as described in the Material & Methods section. More extensive and detailed annotations are available in Table 1S.

[b]Pseudo-pilus are secreted and assembled by FPE after cleavage of the signal peptide of class 3 by signal peptidase ComC. Flagellum components are secreted and assembled by FEA (Table 1).

[c]Subcellular location follow the GO (Gene Ontology) for cellular component. Pseudo-pilus and flagellum (GO:00019861) are supramolecular protein complexes (GO:0043234) exposed at cell surface (GO:0009986).
