# Supplementary material for: Subcellular Localization of Extracytoplasmic Proteins in Monoderm Bacteria: Rational Secretomics-Based Strategy for Genomic and Proteomic Analyses
Source: PLoS One. 2012 Aug 9;7(8):e42982. doi: 10.1371/journal.pone.0042982 (PMC3415414; doi:10.1371/journal.pone.0042982)
Supplement: Table S6 — Summarised information about protein categories, secretion pathways and GO terms for IMPs, lipoproteins, cell-wall proteins, subunits of supramolecular cell-surface appendages and exoproteins, respectively, as predicted by the secretomics-based method in L. monocytogenes EGD-e. (PDF) [file pone.0042982.s006.pdf]

Table 6S: **The 80 secreted exoproteins in *L. monocytogenes* EGD-e as revealed by the secretomics-based method.**

Protein ID	Annotation[a]	Secretion pathway[b]	GO[c]
Lmo0017	5'-nucleotidase/2',3'-cyclic phosphodiesterase, UDP-sugar hydrolase	Sec, SPase I	0005576
Lmo0019	Cell wall hydrolase with SH3 domain	Sec, SPase I	0005576
Lmo0086	Protein of unknown function with fibronectin type III-like fold	Sec, SPase I	0005576
Lmo0087	Protein of unknown function	Sec, SPase I	0005576
Lmo0105	Chitinase, ChiB	Sec, SPase I	0005576
Lmo0201	Phosphatidylinositol phospholipase C, PlcA	Sec, SPase I	0005576
Lmo0202	Listeriolysin O (Thiol-activated cytolysin) (LLO) Hly (HlyA) (LisA)	Sec, SPase I	0005576
Lmo0203	Zinc metalloproteinase, Mpl (PrtA)	Sec, SPase I	0005576
Lmo0205	Phosphatidylcholine phospholipase C, PlcB (prtC)	Sec, SPase I	0005576
Lmo0206	Protein of unknown function	Sec, SPase I	0005576
Lmo0275	Metallo-β-lactamase	Sec, SPase I	0005576
Lmo0394	NLP/P60-type cell wall hydrolase	Sec, SPase I	0005576
Lmo0408	Protein of unknown function with DUF1312 domain, COG5341	Sec, SPase I	0005576
Lmo0412	Potein of unknown function	Sec, SPase I	0005576
Lmo0415	Peptidoglycan GlcNAc deacetylase	Sec, SPase I	0005576
Lmo0438	Nuclear targeted protein A, IntA	Sec, SPase I	0005576
Lmo0461	Protein of unknown function	Sec, SPase I	0005576
Lmo0462	Protein of unknown function	Sec, SPase I	0005576
Lmo0516	Poly-β-glutamate biosynthesis enzyme	Sec, SPase I	0005576
Lmo0540	β-lactamase-type transpeptidase	Sec, SPase I	0005576
Lmo0601	Protein of unknown function with WD-40 repeat, COG3595	Sec, SPase I	0005576
Lmo0638	Protein of unknown function	Sec, SPase I	0005576
Lmo0671	Protein of unknown function	Sec, SPase I	0005576
Lmo0724	Protein of unknown function UCP032442 type, COG4990	Sec, SPase I	0005576
Lmo0745	Protein of unknown function	Sec, SPase I	0005576
Lmo0755	SGNH hydrolase-type esterase	Sec, SPase I	0005576
Lmo0778	Protein of unknown function	Sec, SPase I	0005576

Lmo0849	Amidase	Sec, SPase I	0005576
Lmo0881	Protein of unknown function	Sec, SPase I	0005576
Lmo0950	Protein of unknown function, α/β hydrolase fold, DUF1801 domain, COG4814	Sec, SPase I	0005576
Lmo1104	NLP/P60-type cell wall hydrolase	Sec, SPase I	0005576
Lmo1216	Muramidase flagellum-specific with a single GW domain, FlgJ-type	Sec, SPase I	0005576
Lmo1264	Metalloprotease with zincin-like fold	Sec, SPase I	0005576
Lmo1333	Aminodeoxychorismate lyase	Sec, SPase I	0005576
Lmo1334	Protein of unknown function	Sec, SPase I	0005576
Lmo1438	Cell division protein FtsI/Penicillin-binding protein 2, transpeptidase	Sec, SPase I	0005576
Lmo1518	Protein of unknown function	Sec, SPase I	0005576
Lmo1521	N-acetylmuramoyl-L-alanine amidase with a single GW domain, YrvJ-type	Sec, SPase I	0005576
Lmo1585	Peptidase S49	Sec, SPase I	0005576
Lmo1601	Protein of unknown function with TMP (tape measure protein) domain, COG4980	Sec, SPase I	0005576
Lmo1602	Protein of unknown function with DUF948, COG4768	Sec, SPase I	0005576
Lmo1654	Metalloprotease with zincin-like fold	Sec, SPase I	0005576
Lmo1656	Protein of unknown function	Sec, SPase I	0005576
Lmo1752	Protein of unknown function	Sec, SPase I	0005576
Lmo1786	Internalin C, InlC, with LRR (Leucine-rich repeat)	Sec, SPase I	0005576
Lmo1855	Peptidase M15B and M15C, D,D-carboxypeptidase VanY/endolysins	Sec, SPase I	0005576
Lmo1862	SGNH hydrolase-type esterase	Sec, SPase I	0005576
Lmo1883	Chitinase, ChiA	Sec, SPase I	0005576
Lmo1913	Six-hairpin glycosidase-like	Sec, SPase I	0005576
Lmo2027	Protein of unknown function with leucine-rich repeat (LRR) protein, COG4886, internalin-like	Sec, SPase I	0005576
Lmo2039	Cell division protein FtsI/Penicillin-binding protein 2, transpeptidase	Sec, SPase I	0005576
Lmo2074	α/β hydrolase, pepditase S9/S15	Sec, SPase I	0005576
Lmo2093	Sec-translocated protein of unknown function	Sec, SPase I	0005576
Lmo2106	Metallo-dependent phosphatase	Sec, SPase I	0005576
Lmo2119	Protein of unknown function YbbR-like	Sec, SPase I	0005576
Lmo2156	Protein of unknown function YxeA-like with conserved DUF1093 domain	Sec, SPase I	0005576
Lmo2217	Protein of unknown function, COG4980	Sec, SPase I	0005576
Lmo2439	Protein of unknown function YxeA-like, COG5294	Sec, SPase I	0005576

Lmo2444	Glycosyl hydrolase family 31 with galactose-binding like domains	Sec, SPase I	0005576
Lmo2467	Chitin binding protein	Sec, SPase I	0005576
Lmo2470	Protein of unknown function with leucine-rich repeat (LRR) protein, COG4886, internalin-like	Sec, SPase I	0005576
Lmo2504	Peptidase M23	Sec, SPase I	0005576
Lmo2505	Peptidoglycan lytic protein P45 (protein of 45 kDa), Spl (secreted protein with lytic property)	Sec, SPase I	0005576
Lmo2568	Protein of unknown function YxeA-like, COG5294	Sec, SPase I	0005576
Lmo2639	Protein of unknown function with DUF1312 domain, COG5341	Sec, SPase I	0005576
Lmo2686	Protein of unknown function	Sec, SPase I	0005576
Lmo2713	YkuD family protein (former ErfK/YbiS/YcfS/YnhG family protein)	Sec, SPase I	0005576
Lmo2754	Peptidase S11, D-alanyl-D-alanine carboxypeptidase A	Sec, SPase I	0005576
Lmo2776	Bacteriocin, lactococcin 972	Sec, SPase I	0005576
Lmo0367	Dyp-type peroxidase	Tat, SPase I	0005576
Lmo0335	Bacteriocin, class II microcin	ABC	0005576
Lmo0615	Bacteriocin	ABC	0005576
Lmo2574	Bacteriocin	ABC	0005576
Lmo2753	Bacteriocin leaderless	ABC	0005576
Lmo0129	Cell wall hydrolase, autolysin	Holin	0005576
Lmo2278	Endolysin Ply118	Holin	0005576
Lmo2284	Polygalacturonase	Holin	0005576
Lmo0056	WXG100-A, Lmesat6	Wss	0005576
Lmo0062	WXG100 protein secretion system, peripheral component, EsaC	Wss	0005576
Lmo0063	WXG100-B	Wss	0005576

[a]Some annotations were corrected respective to the similarity search performed as described in the Material & Methods section. More extensive and detailed annotations are available in Table 1S.

[b]Exoprotein with a SP is cleaved by signal peptidase of Type I (SPase I) when secreted *via* Sec or Tat or concomitantly to secretion *via* ABC transporter (Table 1).

[c]Subcellular location follow the GO (Gene Ontology) for cellular component. Exoprotein as released into the extracellular milieu (GO:0005576).
