# Supplementary material for: Subcellular Localization of Extracytoplasmic Proteins in Monoderm Bacteria: Rational Secretomics-Based Strategy for Genomic and Proteomic Analyses
Source: PLoS One. 2012 Aug 9;7(8):e42982. doi: 10.1371/journal.pone.0042982 (PMC3415414; doi:10.1371/journal.pone.0042982)
Supplement: Table S8 — Secretomic analysis of extracytoplasmic proteins experimentally identified in L. monocytogenes EGD-e. (PDF) [file pone.0042982.s008.pdf]

Table 8S: **Secretomic analysis of extracytoplasmic proteins experimentally identified in *L. monocytogenes* EGD-e.**

Protein ID	Annotation[a]	Secretomic analysis				Experimental analysis			References
		Protein type[b]	SCL[c]	Secretion pathway[d]	SP[e]	Fraction[f]	Secretion pathway[g]	C+1[h]	
Exoprotein									
Lmo0201	Phosphatidylinositol phospholipase C, PlcA		EC	Sec;SPase I	I (30)	mb/sn		30	[1,2,3]
Lmo0202	Listeriolysin O (LLO), Hly		EC	Sec;SPase I	I (25)	mb/cw/sn	SipZ	25	[1,2,3,4,5,6]
Lmo0203	Zinc metalloproteinase, Mpl (PrtA)		EC	Sec;SPase I	I (25)	sn			[1,2]
Lmo0205	Phosphatidylcholine phospholipase C, PlcB (PrtC)		EC	Sec;SPase I	I (26)	mb/sn	SipZ		[1,2,3,4]
Lmo0412	Sec-translocated protein of unknown function		EC	Sec;SPase I	I (59)	sn			[1]
Lmo0540	β-lactamase-type transpeptidase		EC	Sec;SPase I	I (47)	sn			[1]
Lmo0601	Protein of unknown function with WD-40 repeat, COG3595		EC	Sec;SPase I	I (29)	mb			[3]
Lmo0950	Protein of unknown function with DUF1801 domain, COG4814		EC	Sec;SPase I	I (33)	mb/se/sn			[2,3,7]
Lmo1216	Muramidase flagellum-specific with a single GW domain, FlgJ-type		EC	Sec;SPase I	I (21)	sn			[2]
Lmo1333	Aminodeoxychorismate lyase protein family		EC	Sec;SPase I	I (33)	mb/sn			[1,2,3]
Lmo1438	Cell division protein FtsI/Penicillin-binding protein 2, transpeptidase		EC	Sec;SPase I	I (51)	sn			[1,2]
Lmo1521	N-acetylmuramoyl-L-alanine amidase with a single GW domain, YrvJ-type		EC	Sec;SPase I	I (30)	sn			[1,2]
Lmo1601	Protein of unknown function of unknown function, COG4980		EC	Sec;SPase I	I (26)	mb			[3]
Lmo1602	Protein of unknown function with DUF948, COG4768		EC	Sec;SPase I	I (21)	mb			[3]
Lmo1752	Protein of unknown function of unknown function		EC	Sec;SPase I	I (34)	sn			[1,2]
Lmo1786	Internalin C, InlC, with LRR		EC	Sec;SPase I	I (34)	sn			[1,2]
Lmo1862	SGNH hydrolase-type esterase		EC	Sec;SPase I	I (26)	mb			[3]
Lmo1883	Chitinase, ChiA		EC	Sec;SPase I	I (30)	sn			[1,2]
Lmo2039	Cell division protein FtsI/Penicillin-binding protein 2, transpeptidase		EC	Sec;SPase I	I (49)	se/sn	SecA2		[1,8]
Lmo2119	Protein of unknown function, YbbR-like		EC	Sec;SPase I	I (29)	mb			[3]
Lmo2156	Protein of unknown function YxeA-like with DUF1093 domain		EC	Sec;SPase I	I (31)	sn			[1,2]
Lmo2504	Peptidase M23		EC	Sec;SPase I	I (26)	sn			[1,2]
Lmo2505	Peptidoglycan lytic protein P45 (protein of 45 kDa), Spl		EC	Sec;SPase I	I (28)	mb/cw/se/sn		28	[1,2,3,6,7,9,10]
Lmo2754	Peptidase S11, D-alanyl-D-alanine carboxypeptidase A		EC	Sec;SPase I	I (32)	sn			[2]
Lmo0129	Cell wall hydrolase, autolysin		EC	Holin	-	sn			[2]
Lmo2574	Bacteriocin		EC	ABC	ABC (21)	mb			[3]
CW-protein									
Lmo0130	Bifunctional phosphodiesterase/nucleotidase, CpdB	LPXTG	CW/CS	Sec;SPase I-SrtA	I (35)	cw/sn	SrtA	35	[1,6,9,10]
Lmo0160	Collagen-binding protein with LPXTG domain	LPXTG	CW/CS	Sec;SPase I-SrtA	I (37)	cw	SrtA		[6,9,10]

Lmo0262	Internalin G, InlG, with LRR and LPXTG domain	LPXTG	CW/CS	Sec;SPase I-SrtA	I (28)	cw	SrtA	[6,9,10]
Lmo0263	Internalin H, InlH, with LRR and LPXTG domain	LPXTG	CW/CS	Sec;SPase I-SrtA	I (31)	cw/se/sn	SrtA	[2,6,9,10,11]
Lmo0320	Virulence protein, Vip, recognition of Gp96 receptor	LPXTG	CW/CS	Sec;SPase I-SrtA	I (32)	se		[12]
Lmo0327	Protein of unknown function, DUF1085, internalin-like	LPXTG	CW/CS	Sec;SPase I-SrtA	I (27)	cw	SrtA	[6,9,10]
Lmo0331	Protein of unknown function with LRR, PKD, internalin-like	LPXTG	CW/CS	Sec;SPase I-SrtA	I (28)	cw		[6]
Lmo0333	Internalin I, InlI, with LRR, PKD motif	LPXTG	CW/CS	Sec;SPase I-SrtA	I (29)	cw		[6]
Lmo0433	Internalin A, InlA, with LRR	LPXTG	CW/CS	Sec;SPase I-SrtA	I (36)	cw/se/sn	SipZX,SrtA	[2,4,6,9,10,13,14]
Lmo0435	Biofilm-associated protein, BapL	LPXTG	CW/CS	Sec;SPase I-SrtA	I (30)	cw		[6]
Lmo0514	Protein of unknown function with LRR, PKD motif, internalin	LPXTG	CW/CS	Sec;SPase I-SrtA	I (27)	cw		[6]
Lmo0610	Protein of unknown function with LRR, PKD motif, internalin	LPXTG	CW/CS	Sec;SPase I-SrtA	I (29)	cw	SrtA	[6,9,10]
Lmo0842	Invasin/intimin cell-adhesion protein with Ig-like motif	LPXTG	CW/CS	Sec;SPase I-SrtA	I (34)	cw	SrtA	[6,9,10]
Lmo0880	Collagen-binding protein	LPXTG-LysM	CW/CS	Sec;SPase I-SrtA	I (26)	cw/sn	SrtA	[2,6,9,10]
Lmo1290	Internalin K, InlK, Recruitment of the Major Vault Protein	LPXTG	CW/CS	Sec;SPase I-SrtA	I (31)	se		[15]
Lmo1413	Protein of unknown function with DUF1085 motif	LPXTG	CW/CS	Sec;SPase I-SrtA	I (30)	cw	SrtA	[6,9,10]
Lmo1666	Bacterial adhesin, hyaline/cadherin domain, RGD motif	LPXTG	CW/CS	Sec;SPase I-SrtA	I (27)	cw/sn	SrtA	[2] [6,9,10]
Lmo2085	Collagen-binding protein	LPXTG	CW/CS	Sec;SPase I-SrtA	I (28)	cw	SrtA	[6,9,10]
Lmo2178	Collagen-binding protein	LPXTG	CW/CS	Sec;SPase I-SrtA	I (30)	cw		[6]
Lmo2185	Surface virulence-associated protein, SvpA, NEAr transporter	LPXTG	CW/CS	Sec;SPase I-SrtB	I (29)	cw/se/sn	SrtB	[2,6,9,10,16,17,18]
Lmo2186	Surface virulence-associated protein, SvpB, NEAr transporter	LPXTG	CW/CS	Sec;SPase I-SrtB	I (28)	cw/sn	SrtB	[2,6,9,10]
Lmo2714	Protein of unknown function	LPXTG	CW/CS	Sec;SPase I-SrtA	I (27)	cw/sn	SrtA	[1,2,6,9,10]
Lmo2821	Internalin J, InlJ, with LRR (Leucine-rich repeat)	LPXTG	CW/CS	Sec;SPase I-SrtA	I (26)	se		[19]
Lmo0585	Sec-translocated protein of unknown function, CscB-like	WXL	CW/CS	Sec;SPase I	I (27)	sn		[1]
Lmo1851	Peptidase S41	PGBD1	CW/CS	Sec;SPase I	I (57)	mb/sn		[2,3]
Lmo0434	Internalin B, InlB, with LRR (Leucine-rich repeat)	GW	CW/CS	Sec;SPase I	I (36)	se/sn	SipZ	[2,4,7,20]
Lmo2203	N-acetylmuramoyl-L-alanine amidas	GW	CW/CS	Sec;SPase I	I (36)	sn		[1]
Lmo2558	N-acetylmuramoyl-L-alanine amidase family 2, autolysin, Ami	GW	CW/CS	Sec;SPase I	I (31)	se/sn	GW	[2,7,21]
Lmo1076	N-acetylmuramoyl-L-alanine amidase, Auto	GW	CW/CS	Sec;SPase I	I (27)	se/sn		[2,22]
Lmo2591	N-acetylmuramoyl-L-alanine amidase	GW	CW/CS	Sec;SPase I	I (27)	sn		[1,2]
Lmo0582	Cell-wall hydrolase, invasion associated protein, Iap (P60)	LysM	CW/CS	Sec;SPase I	I (26)	mb/cw/se/sn	SecA2 26	[1,2,3,6,7,8,9,10,23,24]
Lmo2522	Protein of unknown function	LysM	CW/CS	Sec;SPase I	I (25)	sn		[1,2]
Lmo2691	Autolysin, N-acetylmuramoyl-L-alanine amidase, MurA	LysM	CW/CS	Sec;SPase I	I (53)	cw/sn		[1,2,6,7,9,10]
Lmo1303	Cell division suppressor protein, YneA	ssIMP II-LysM	MB/CW/CS	Sec;YidC	SA	sn		[2]
Lmo1941	Protein of unknown function, YpbE-like	ssIMP II-LysM	MB/CW/CS	Sec;YidC	SA	mb/cw		[3,6]
IMP								
Lmo0204	Actin-assembly inducing protein, ActA	ssIMP I	MB/CS	Sec;SPase I	I (30)	mb/cw/sn	SipZX	[1,2,3,4,6]
Lmo0289	Signal transduction YycH protein	ssIMP II	MB/CS	Sec;YidC	SA	mb		[3]
Lmo0971	D-alanine esterification of teichoic acid, DltD	ssIMP II	MB/CS	Sec;YidC	SA	mb		[3]

Lmo1499	Aminodeoxychorismate lyase	ssIMP II	MB/CS	Sec;YidC	SA	mb	[3]
Lmo1547	Cell shape-determining protein, MreC	ssIMP II	MB/CS	Sec;YidC	SA	mb	[3]
Lmo2410	Protein of unknown function	ssIMP I	MB/CS	Sec;SPase I	I (34)	sn	[2]
Lmo0186	Protein of unknown function with 3D and G5 domains	ssIMP II	MB/CS	Sec;YidC	SA	sn	[1,2]
Lmo0217	Septum formation initiator	ssIMP II	MB/CS	Sec;YidC	SA	sn	[2]
Lmo0292	Serine protease with C-terminal PDZ/DHR/GLGF domain	ssIMP II	MB/CS	YidC	-	mb/se/sn	[2,3,7]
Lmo0290	Protein of unknown function, COG4853	ssIMP II	MB/CS	Sec;YidC	SA	mb	[3]
Lmo0392	Protein of unknown function with DUF1432, COG4864	ssIMP II	MB/CS	Sec;YidC	SA	mb	[3]
Lmo0441	Cell division protein FtsI/Penicillin-binding protein 2	ssIMP II	MB/CS	Sec;YidC	SA	mb	[3]
Lmo0443	Transcriptional regulator	ssIMP II	MB/CS	Sec;YidC	SA	mb/se/sn	[1,2,3,7]
Lmo0929	Sortase A, SrtA	ssIMP II	MB/CS	Sec;YidC	SA	mb	[3]
Lmo0962	Listeria epitope, LemA	ssIMP II	MB/CS	Sec;YidC	SA	mb	[3]
Lmo1026	Transcriptional regulator	ssIMP II	MB/CS	Sec;YidC	SA	mb	[3]
Lmo1075	Teichoic acids export protein ATP-binding subunit	ssIMP II	MB/CS	YidC	-	mb	[3]
Lmo1395	DNA-binding λ repressor-like, COG1426	ssIMP II	MB/CS	YidC	-	mb/sn	[2,3]
Lmo1399	HD superfamily hydrolase	ssIMP II	MB/CS	Sec;YidC	SA	mb	[3]
Lmo1495	Protein of unknown function with DUF1510 domain	ssIMP II	MB/CS	Sec;YidC	SA	mb	[3]
Lmo1511	Lysophospholipase	ssIMP II	MB/CS	Sec;YidC	SA	mb	[3]
Lmo1529	Sec transcolon, subunit YajC	ssIMP II	MB/CS	Sec;YidC	SA	mb	[3]
Lmo1663	Asparagine synthase, glutamine-hydrolyzing	ssIMP II	MB/CS	YidC	-	mb	[3]
Lmo1715	S-adenosyl-L-methionine-dependent methyltransferase type 11	ssIMP II	MB/CS	Sec;YidC	SA	sn	[2]
Lmo1820	Serine/threonine protein kinase	ssIMP II	MB/CS	YidC	-	mb	[3]
Lmo1861	Protein of unknown function, COG4698	ssIMP II	MB/CS	Sec;YidC	SA	mb	[3]
Lmo1892	Carboxypeptidase, PbpA, Glycosyl transferase family 51	ssIMP II	MB/CS	Sec;YidC	SA	mb/cw	[3,9,10]
Lmo1898	Protein of unknown function, COG5353	ssIMP II	MB/CS	Sec;YidC	SA	mb	[3]
Lmo1927	3-dehydroquinate synthase, AroB	ssIMP II	MB/CS	YidC	-	mb	[3]
Lmo2036	UDP-N-acetylmuramoylalanine-D-glutamate ligase, MurD	ssIMP II	MB/CS	Sec;YidC	SA	mb	[3]
Lmo2056	Ca^{2+} chelating serine protease with SCP/PR1 domain	ssIMP II	MB/CS	Sec;YidC	SA	mb	[3]
Lmo2089	Esterase/lipase	ssIMP II	MB/CS	Sec;YidC	SA	mb	[3]
Lmo2229	Carboxypeptidase (penicillin-binding protein)	ssIMP II	MB/CS	Sec;YidC	SA	mb	[3]
Lmo2486	Protein of unknown function, UCP012569 type, COG3595	ssIMP II	MB/CS	Sec;YidC	SA	mb	[3]
Lmo2518	Transcriptional regulator	ssIMP II	MB/CS	Sec;YidC	SA	mb/sn	[1,2,3]
Lmo2710	Protein of unknown function	ssIMP II	MB/CS	Sec;YidC	SA	mb	[3]
Lmo1351	Rhodanese-related sulfurtransferase	ssIMP III	MB/CS	YidC	-	mb	[3]
Lmo1594	Negative regulator of septation ring formation	ssIMP III	MB/CS	YidC	-	mb	[3]
Lmo2533	F_0F_1 ATP synthase, subunit B	ssIMP III	MB/CS	YidC	-	mb	[3]
Lmo0008	Cardiolipin synthetase	msIMP	MB/CS	Sec;YidC	SA	mb	[3]

Lmo0014	Cytochrome AA3-600 quinol oxidase, subunit I	msIMP	MB/CS	YidC	-	mb	[3]
Lmo0015	Cytochrome AA3-600 quinol oxidase, subunit III	msIMP	MB/CS	Sec;YidC	SA	mb	[3]
Lmo0098	Phosphotransferase system, mannose/fructose family IID	msIMP	MB/CS	YidC	-	mb	[3]
Lmo0136	ABC-type dipeptide/oligopeptide/nickel transporter, permease	msIMP	MB/CS	Sec;SPase I	I (33)	mb	[3]
Lmo0220	Peptidase M41, FtsH	msIMP	MB/CS	Sec;YidC	SA	mb	[3]
Lmo0234	Protein of unknown function, PIN domain, COG4956	msIMP	MB/CS	Sec;SPase I	I (65)	mb	[3]
Lmo0288	Signal transduction histidine kinase	msIMP	MB/CS	Sec;YidC	SA	mb	[3]
Lmo0296	Protein of unknown function	msIMP	MB/CS	Sec;YidC	SA	mb	[3]
Lmo0524	Sulfate permease, transporter MFS superfamily	msIMP	MB/CS	Sec;YidC	SA	mb	[3]
Lmo0531	Protein of unknown function with GGDEF domain	msIMP	MB/CS	Sec;YidC	SA	mb	[3]
Lmo0584	Permease	msIMP	MB/CS	Sec;YidC	SA	mb	[3]
Lmo0607	ABC bacteriocin exporter, peptidase, ATP-binding/permease	msIMP	MB/CS	Sec;YidC	SA	mb	[3]
Lmo0608	ABC bacteriocin exporters, peptidase, ATP-binding/permease	msIMP	MB/CS	Sec;YidC	SA	mb	[3]
Lmo0626	Protein of unknown function	msIMP	MB/CS	Sec;SPase I	I (32)	mb	[3]
Lmo0644	Phosphoglycerol transferase, alkaline phosphatase superfamily	msIMP	MB/CS	Sec;YidC	SA	sn	[1,2]
Lmo0650	Phage infection protein, YhgE	msIMP	MB/CS	Sec;SPase I	I (37)	mb	[3]
Lmo0653	Protein of unknown function with DUF975 domain, COG5523	msIMP	MB/CS	Sec;YidC	SA	mb	[3]
Lmo0680	Flagellar export apparatus, membrane subunit FlhA	msIMP	MB/CS	Sec;SPase I	I (42)	sn	[2]
Lmo0781	Phosphotransferase system, mannose family IID	msIMP	MB/CS	YidC	-	mb	[3]
Lmo0782	Phosphotransferase system, sorbose-specific IIC subunit	msIMP	MB/CS	Sec;YidC	SA	mb	[3]
Lmo0841	Cation transport ATPase	msIMP	MB/CS	Sec;YidC	SA	mb	[3]
Lmo0847	ABC-type amino acid transport system, permease	msIMP	MB/CS	Sec;SPase I	I (31)	mb	[3]
Lmo0927	Phosphoglycerol transferase, alkaline phosphatase superfamily	msIMP	MB/CS	Sec;YidC	SA	mb/sn	[1,2,3]
Lmo0933	Phosphate 4-deoxy-4-formamido-L-arabinose transferase	msIMP	MB/CS	YidC	-	mb	[3]
Lmo0952	Protein of unknown function with uncleaved signal peptide	msIMP	MB/CS	Sec;YidC	SA	mb	[3]
Lmo1015	ABC-type betaine transport system, permease, GbuB	msIMP	MB/CS	YidC	-	mb	[3]
Lmo1061	Signal transduction histidine kinase, BaeS	msIMP	MB/CS	Sec;YidC	SA	sn	[2]
Lmo1064	Mg^{2+} and Co^{2+} transporters, CorA-like	msIMP	MB/CS	YidC	-	mb	[3]
Lmo1261	Protein of unknown function with GYF domain	msIMP	MB/CS	YidC	-	mb	[3]
Lmo1291	Acyltransferase family 3	msIMP	MB/CS	Sec;YidC	SA	se/sn	[2,7]
Lmo1422	ABC-type betaine uptake system, bile exclusion system, BilEB	msIMP	MB/CS	YidC	-	mb	[3]
Lmo1424	Ion transporters of the NRAMP family, MntH-like	msIMP	MB/CS	Sec;YidC	SA	mb	[3]
Lmo1427	ABC-type betaine transport systems, permease, OpuCB	msIMP	MB/CS	YidC	-	mb	[3]
Lmo1440	Protein of unknown function with DUF1189 domain	msIMP	MB/CS	Sec;YidC	SA	mb	[3]
Lmo1516	Ammonium permease	msIMP	MB/CS	YidC	-	mb	[3]
Lmo1527	Sec transcolon, bifunctional subunit SecDF	msIMP	MB/CS	Sec;SPase I	I (24)	mb	[3]
Lmo1626	Protein of unknown function	msIMP	MB/CS	Sec;YidC	SA	mb	[3]

Lmo1637	ABC transporter, copper enzyme maturation, permease	msIMP	MB/CS	Sec;YidC	SA	mb		[3]
Lmo1695	Lysyl-tRNA synthetase	msIMP	MB/CS	Sec;YidC	SA	mb		[3]
Lmo1740	ABC-type amino acid transport system, permease	msIMP	MB/CS	YidC	-	mb		[3]
Lmo1741	Signal transduction histidine kinase	msIMP	MB/CS	Sec;YidC	SA	mb		[3]
Lmo1839	Xanthine/uracil permease, PyrP	msIMP	MB/CS	Sec;YidC	SA	mb		[3]
Lmo1853	Heavy metal translocating P-type ATPase	msIMP	MB/CS	YidC	-	mb		[3]
Lmo1884	Xanthine permease	msIMP	MB/CS	Sec;YidC	SA	mb		[3]
Lmo1919	Zinc metallopeptidase	msIMP	MB/CS	YidC	-	mb		[3]
Lmo2017	Phosphatidic acid phosphatase type 2/haloperoxidase	msIMP	MB/CS	Sec;SPase I	I (29)	mb		[3]
Lmo2029	Protein of unknown function, YGGT-like, COG0762	msIMP	MB/CS	YidC	-	mb		[3]
Lmo2059	Voltage-gated potassium channel	msIMP	MB/CS	Sec;YidC	SA	mb		[3]
Lmo2064	Large-conductance mechanosensitive channel, MscL	msIMP	MB/CS	Sec;YidC	SA	mb		[3]
Lmo2120	Protein of unknown function, COG1624	msIMP	MB/CS	YidC	-	mb		[3]
Lmo2140	ABC-type Na^+ efflux pump, permease component	msIMP	MB/CS	Sec;SPase I	I (45)	mb		[3]
Lmo2194	ABC dipeptide/oligopeptide/nickel transporter, permease	msIMP	MB/CS	Sec;YidC	SA	mb		[3]
Lmo2195	ABC dipeptide/oligopeptide/nickel transporter, permease	msIMP	MB/CS	Sec;YidC	SA	mb		[3]
Lmo2221	Protein of unknown function, COG4717	msIMP	MB/CS	YidC	-	mb		[3]
Lmo2232	Hemolysin, CBS domains	msIMP	MB/CS	Sec;YidC	SA	mb		[3]
Lmo2254	Xanthine/uracil/vitamin C permease	msIMP	MB/CS	Sec;YidC	SA	mb		[3]
Lmo2287	Tape measure domain bacteriophage A118	msIMP	MB/CS	YidC	-	sn		[2]
Lmo2335	2-O-mannosyl-D-glycerate specific PTS transporter	msIMP	MB/CS	YidC	-	mb		[3]
Lmo2360	Phage infection protein, YhgE	msIMP	MB/CS	Sec;SPase I	I (33)	mb		[3]
Lmo2367	Phosphoglucose isomerase	msIMP	MB/CS	YidC	-	mb		[3]
Lmo2371	ABC transporter, lipoprotein release, permease	msIMP	MB/CS	Sec;SPase I	I (41)	mb		[3]
Lmo2418	ABC-type metal ion transport system, permease	msIMP	MB/CS	Sec;YidC	SA	mb		[3]
Lmo2469	Amino acid/polyamine transporter I	msIMP	MB/CS	Sec;YidC	SA	mb		[3]
Lmo2503	Cardiolipin synthetase	msIMP	MB/CS	Sec;YidC	SA	mb		[3]
Lmo2526	UDP-N-acetylglucosamine enolpyruvyl transferase	msIMP	MB/CS	YidC	-	se/sn	SecA2	[8]
Lmo2535	F_0F_1 ATP synthase, subunit A	msIMP	MB/CS	Sec;YidC	SA	mb		[3]
Lmo2550	Phosphate 4-deoxy-4-formamido-L-arabinose transferase	msIMP	MB/CS	YidC	-	mb/sn		[2,3]
Lmo2612	Sec translocon, subunit SecY	msIMP	MB/CS	Sec;SPase I	I (42)	mb		[3]
Lmo2638	NADH dehydrogenase, FAD-containing subunit	msIMP	MB/CS	YidC	-	mb		[3]
Lmo2681	K^+ transporting ATPase, B subunit, KdpB	msIMP	MB/CS	Sec;YidC	SA	sn		[2]
Lmo2715	ABC cysteine exporter, permease/ATP-binding protein, CydD	msIMP	MB/CS	Sec;YidC	SA	mb		[3]
Lmo2718	Cytochrome bd-type quinol oxidase, subunit 1, CydA	msIMP	MB/CS	YidC	-	mb		[3]
Lmo2745	ABC bacteriocin exporter, peptidase, ATP-binding/permease	msIMP	MB/CS	Sec;SPase I	I (47)	mb		[3]
Lmo2751	ABC bacteriocin exporter, peptidase, ATP-binding/permease	msIMP	MB/CS	Sec;YidC	SA	mb		[3]

Lmo0013	AA3-600 quinol oxidase subunit II, QoxA	msIMP-Lipo	MB/CS/EC	Sec;YidC-Lgt-SPase II;C+2=G	II (23)	mb/se/sn		[1,2,3,7]
Lmo0641	Heavy metal translocating P-type ATPase	msIMP-Lipo	MB/CS	Sec;YidC-Lgt-SPase II	II (25)	mb		[3]
Lmo2854	YidC insertase, OxaA-like protein, OxaA2 (SpoIIIJ)	msIMP-Lipo	MB/CS/EC	Sec;YidC-Lgt-SPase II;C+2=G	II (27)	mb		[3]
Lipoprotein								
Lmo0047	Peptidase M4, PepSY		MB/CS	Sec;Lgt-SPase II	II (21)	mb/se/*	Lgt	[3,7,25]
Lmo0135	ABC-type dipeptide transport system, substrate-binding protein family 5		MB/CS/EC	Sec;Lgt-SPase II;C+2=G	II (20)	mb/se/sn/*	Lgt	[1,2,3,7,25]
Lmo0152	ABC-type oligopeptide transport system, substrate-binding protein family 5		MB/CS/EC	Sec;Lgt-SPase II;C+2=G	II (22)	mb/sn/*	Lgt	[2,3,25]
Lmo0153	ABC-type metal ion transport system, substrate-binding protein, surface adhesin		MB/CS/EC	Sec;Lgt-SPase II;C+2=G	II (21)	mb/sn		[2,3]
Lmo0181	ABC-type sugar transport system, substrate-binding protein family 1		MB/CS/EC	Sec;Lgt-SPase II;C+2=G	II (21)	*	Lgt	[25]
Lmo0285	ABC-type metal ion transport system, substrate-binding protein, surface antigen		MB/CS/EC	Sec;Lgt-SPase II;C+2=G	II (23)	mb/se		[3,7]
Lmo0355	Fumarate reductase flavoprotein subunit		MB/CS/EC	Sec;Lgt-SPase II;C+2=G	II (22)	mb		[3]
Lmo0541	ABC-type Fe^{3+}-hydroxamate transport system, substrate-binding protein		MB/CS/EC	Sec;Lgt-SPase II;C+2=G	II (23)	mb/*	Lgt	[3,25]
Lmo0791	Lipoprotein of unknown function, YcdA-like spore lipoprotein		MB/CS/EC	Sec;Lgt-SPase II;C+2=G	II (21)	mb/se/*	Lgt	[3,7,25]
Lmo0945	Metallo-β-lactamase family protein, DNA-binding ComEC/Rec2-like protein		MB/CS	Sec;Lgt-SPase II	II (19)	*	Lgt	[25]
Lmo1016	ABC-type proline/glycine betaine transport system, substrate-binding protein		MB/CS/EC	Sec;Lgt-SPase II;C+2=G	II (21)	mb		[3]
Lmo1041	ABC-type molybdate transport system, substrate-binding protein		MB/CS	Sec;Lgt-SPase II	II (19)	mb/se		[3,7]
Lmo1068	Lipoprotein of unknown function		MB/CS/EC	Sec;Lgt-SPase II;C+2=G	II (20)	mb/se/sn/*	Lgt	[1,2,3,7,25]
Lmo1073	ABC-type Fe^{3+}-hydroxamate transport system, substrate-binding protein		MB/CS/EC	Sec;Lgt-SPase II;C+2=G	II (19)	se/*	Lgt	[7,25]
Lmo1388	CD4+ T-cell stimulating antigen, TcsA, ABC transporter, binding protein		MB/CS/EC	Sec;Lgt-SPase II;C+2=G	II (23)	mb/se/sn/*	SecA2,Lgt	[1,2,3,7,8,25]
Lmo1426	ABC-type choline transport system, osmoprotectant, binding protein, OpuCC		MB/CS	Sec;Lgt-SPase II	II (23)	mb/se		[3,7]
Lmo1671	ABC-type metal ion transport system, substrate-binding protein		MB/CS	Sec;Lgt-SPase II	II (22)	mb/se/sn/*	Lgt	[2,3,7,25]
Lmo1730	ABC-type sugar transport system, substrate-binding protein component		MB/CS/EC	Sec;Lgt-SPase II;C+2=G	II (22)	se		[7]
Lmo1738	ABC-type signal transduction system, substrate-binding protein family 3		MB/CS	Sec;Lgt-SPase II	II (20)	mb/se/sn/*	Lgt	[2,3,7,25]
Lmo1757	Sex pheromone cAM373 biosynthesis, CamS		MB/CS	Sec;Lgt-SPase II	II (18)	mb/se/*	Lgt	[3,7,25]
Lmo1800	Receptor-linked protein tyrosine/serine phosphatase		MB/CS/EC	Sec;Lgt-SPase II;C+2=G	II (22)	*	Lgt	[25]
Lmo1847	ABC-type metal transport system, binding protein, surface adhesin, LpeA		MB/CS	Sec;Lgt-SPase II	II (19)	mb/se/sn/*	Lgt	[2,3,7,25,26]
Lmo1959	ABC-type Fe^{3+}-hydroxamate transport system, substrate-binding protein		MB/CS/EC	Sec;Lgt-SPase II;C+2=G	II (21)	mb		[3]
Lmo2079	Lipoprotein of unknown function		MB/CS/EC	Sec;Lgt-SPase II;C+2=G	II (21)	sn/*	Lgt	[2,25]
Lmo2125	ABC-type maltose transporter, substrate-binding protein family 1		MB/CS/EC	Sec;Lgt-SPase II;C+2=G	II (23)	se	SecA2	[8]
Lmo2196	ABC-type oligopeptide transport system, substrate-binding protein family 5		MB/CS/EC	Sec;Lgt-SPase II;C+2=G	II (23)	mb/se/sn/*	SecA2,Lgt	[1,2,3,8,25]
Lmo2219	Peptidylprolyl isomerase, foldase, PrsA2		MB/CS/EC	Sec;Lgt-SPase II;C+2=G	II (21)	mb/se/*	LspA,Lgt	[3,7,25,27]
Lmo2331	Lipoprotein of unknown function		MB/CS/EC	Sec;Lgt-SPase II;C+2=G	II (22)	mb/se	Lgt	[3,7,25]
Lmo2349	ABC-type signal transduction system, substrate-binding protein family 3		MB/CS/EC	Sec;Lgt-SPase II;C+2=G	II (21)	mb/sn		[2,3]
Lmo2416	Lipoprotein of unknown function		MB/CS	Sec;Lgt-SPase II	II (21)	sn/*	Lgt	[2,25]
Lmo2417	ABC-type metal ion transport system, substrate-binding protein, surface antigen		MB/CS/EC	Sec;Lgt-SPase II;C+2=G	II (20)	mb/se/*	Lgt	[3,7,25]
Lmo2431	ABC-type Fe^{3+}-hydroxamate transport system, substrate-binding protein		MB/CS/EC	Sec;Lgt-SPase II;C+2=G	II (20)	mb/*	Lgt	[3,25]
Lmo2569	ABC-type oligopeptide transport system, substrate-binding protein		MB/CS	Sec;Lgt-SPase II	II (23)	mb/*	Lgt	[3,25]

Lmo2595	Lipoprotein of unknown function	MB/CS/EC	Sec;Lgt-SPase II;C+2=G	II (22)	*	Lgt	[25]
Lmo2636	Thiamine biosynthesis lipoprotein ApbE-like	MB/CS/EC	Sec;Lgt-SPase II;C+2=G	II (20)	mb/se/*	Lgt	[3,7,25]
Lmo2637	Immunogen protein, NADH-(ubi-)quinone oxireductase subunit	MB/CS/EC	Sec;Lgt-SPase II;C+2=G	II (23)	mb/se/sn/*	SecA2,Lgt	[2,3,7,8,25]
Cytoprotein							
Lmo0796	Lipid/polyisoprenoid-binding, YceI-like	CP/EC	NC	-	sn		[1,2]
Lmo0900	Protein of unknown function with conserved DUF1801 domain, COG5646	CP/EC	NC	-	sn		[2]
Lmo1439	Manganese-superoxide dismutase, MnSOD	CP/EC	NC	-	se/sn	SecA2	[1,2,7,28]
Lmo2297	Phage minor structural GP20	CP/EC	NC	-	sn		[2]
Lmo2487	Protein of unknown functionYvlB-like, COG3595	CP/EC	NC	-	mb		[3]
Lmo2705	Polyketide cyclase/dehydrase	CP/EC	NC	-	mb		[3]
Lmo0020	DNA-binding transcriptional regulator, FrlR	CP	-	-	mb		[3]
Lmo0044	30S ribosomal protein S6, RpsF	CP	-	-	sn		[2]
Lmo0096	Phosphotransferase system, sorbose subfamily IIB	CP	-	-	mb		[3]
Lmo0118	Antigen A, LmaA	CP	-	-	sn		[2]
Lmo0119	Protein of unknown function	CP	-	-	sn		[2]
Lmo0154	ABC-type Mn/Zn transport system, ATPase	CP	-	-	mb		[3]
Lmo0194	ABC-type macrolide transport system, ATPase	CP	-	-	mb		[3]
Lmo0196	Stage V sporulation protein G	CP	-	-	sn		[1]
Lmo0197	Sporulation stage V, protein G	CP	-	-	mb/se		[3,7]
Lmo0199	Phosphoribosyl pyrophosphokinase	CP	-	-	mb		[3]
Lmo0211	50S ribosomal protein L25	CP	-	-	sn		[1]
Lmo0214	Transcription-repair coupling factor (superfamily II helicase)	CP	-	-	se		[7]
Lmo0223	Cysteine synthase A, CysA	CP	-	-	mb/se/sn		[1,2,3,7]
Lmo0229	Transcriptional repressor of class III stress genes	CP	-	-	mb		[3]
Lmo0237	Glutamyl-tRNA synthetase, class Ic	CP	-	-	mb		[3]
Lmo0248	50S ribosomal protein L11, RplK	CP	-	-	mb		[3]
Lmo0249	50S ribosomal protein L1	CP	-	-	mb		[3]
Lmo0251	50S ribosomal protein L7/L12, RplL	CP	-	-	mb/sn		[2,3]
Lmo0254	Protein of unknown function	CP	-	-	mb		[3]
Lmo0258	DNA-directed RNA polymerase, subunit β	CP	-	-	se/sn	SecA2	[8]
Lmo0259	DNA-directed RNA polymerase, subunit β-prime	CP	-	-	se/sn	SecA2	[8]
Lmo0284	ABC-type metal ion transport system, ATPase	CP	-	-	mb		[3]
Lmo0395	Acyl-CoA N-acyltransferase, GCN5-related, Nat	CP	-	-	sn		[2]
Lmo0407	Protein of unknown function with DUF1149 domain, COG4835	CP	-	-	mb		[3]
Lmo0454	AAA-type ATPase chaperone, MoxR	CP	-	-	mb		[3]
Lmo0526	DNA-binding protein, MerR-like	CP	-	-	sn		[2]
Lmo0539	Tagatose-1,6-bisphosphate aldolase	CP	-	-	sn		[2]

Lmo0560	Glutamate dehydrogenase/leucine dehydrogenase	CP	-	-	mb/sn		[2,3]
Lmo0583	Sec translocase, ATPase, SecA2	CP	-	-	mb		[3]
Lmo0667	ABC-type multidrug transport system, ATPase	CP	-	-	mb		[3]
Lmo0739	β-glucosidase/6-phospho-β-glucosidase/β-galactosidase	CP	-	-	se		[7]
Lmo0756	ABC-type multidrug transport system, ATPase	CP	-	-	mb		[3]
Lmo0774	Sphingosine kinase	CP	-	-	mb		[3]
Lmo0785	Transcriptional antiterminator	CP	-	-	mb		[3]
Lmo0807	ABC-type spermidine/putrescine transport system, ATPase	CP	-	-	mb		[3]
Lmo0848	ABC-type polar amino acid transport system, ATPase	CP	-	-	mb		[3]
Lmo0851	Protein of unknown function	CP	-	-	mb		[3]
Lmo0866	Superfamily II DNA and RNA helicase	CP	-	-	mb		[3]
Lmo0943	DNA-binding protein, ferritin-like, Dps type	CP	-	-	mb/sn		[2,3]
Lmo1002	Phosphotransferase system, phosphocarrier HPr protein	CP	-	-	sn		[2]
Lmo1003	Phosphoenolpyruvate-protein kinase, PTS system EI	CP	-	-	mb		[3]
Lmo1014	ABC-type proline/glycine betaine transport system, ATPase	CP	-	-	mb		[3]
Lmo1023	K^+ transport systems, NAD-binding	CP	-	-	mb		[3]
Lmo1053	Pyruvate/2-oxoglutarate dehydrogenase complex, β subunit, PdhB	CP	-	-	mb/se		[3,7]
Lmo1054	Dihydrolipoamide acyltransferase (E2)	CP	-	-	mb/se	SecA2	[3,7,8]
Lmo1055	Dihydrolipoamide dehydrogenase	CP	-	-	mb/se/sn		[1,3,7]
Lmo1059	Disulfide isomerase with thioredoxin-like fold	CP	-	-	se		[7]
Lmo1067	GTP-binding protein TypA	CP	-	-	mb		[3]
Lmo1072	Pyruvate carboxylase	CP	-	-	mb		[3]
Lmo1077	CDP-glycerol glycerophosphotransferase	CP	-	-	mb		[3]
Lmo1080	Nucleotide-diphospho-sugar transferase, glycosyl transferase family 2	CP	-	-	mb/sn		[2,3]
Lmo1084	dTDP-4-dehydrorhamnose reductase	CP	-	-	mb		[3]
Lmo1087	Threonine dehydrogenase, Zn-dependent dehydrogenase family	CP	-	-	mb		[3]
Lmo1090	Glycosyltransferase involved in cell wall biogenesis	CP	-	-	mb		[3]
Lmo1091	Glycosyl transferase, family 2	CP	-	-	mb		[3]
Lmo1125	Protein of unknown function	CP	-	-	sn		[2]
Lmo1182	Propanediol utilization protein, PduL	CP	-	-	sn		[2]
Lmo1221	Phenylalanyl-tRNA synthetase, class IIc, α subunit	CP	-	-	mb		[3]
Lmo1222	Phenylalanyl-tRNA synthetase, class IIc, β subunit	CP	-	-	mb		[3]
Lmo1267	Trigger factor, FKBP-type peptidyl-prolyl cis-trans isomerase	CP	-	-	mb		[3]
Lmo1279	ATP-dependent protease, HslVU (ClpYQ), ATPase subunit	CP	-	-	mb		[3]
Lmo1299	Glutamine synthetase type I	CP	-	-	mb		[3]
Lmo1305	Transketolase, Tkt	CP	-	-	sn		[2]
Lmo1314	Ribosome recycling factor	CP	-	-	se		[7]

Lmo1357	Acetyl-CoA carboxylase, biotin carboxylase	CP	-	-	mb		[3]
Lmo1360	Tetrahydrofolate dehydrogenase/cyclohydrolase	CP	-	-	mb		[3]
Lmo1364	Cold shock protein, CspA type	CP	-	-	sn		[1]
Lmo1389	ABC-type sugar transport system, ATPase component	CP	-	-	mb		[3]
Lmo1398	Recombinase A, RecA	CP	-	-	mb		[3]
Lmo1421	ABC-type betaine uptake system, bile exclusion system, BilEA	CP	-	-	mb		[3]
Lmo1428	ABC-type proline/glycine betaine transport system, ATPase	CP	-	-	mb		[3]
Lmo1435	Dihydrodipicolinate synthase subfamily	CP	-	-	mb		[3]
Lmo1473	DnaK class I heat-shock chaperone protein	CP	-	-	mb/se/sn	SecA2	[1,2,3,7,8]
Lmo1475	Negative transcriptional regulator of class I heat shock protein	CP	-	-	mb		[3]
Lmo1517	Nitrogen regulatory protein PII	CP	-	-	mb		[3]
Lmo1519	Aspartyl-tRNA synthetase	CP	-	-	mb		[3]
Lmo1544	Septum site-determining protein, MinD	CP	-	-	mb		[3]
Lmo1548	Cell shape determining protein MreB/Mrl	CP	-	-	mb		[3]
Lmo1570	Pyruvate kinase	CP	-	-	mb		[3]
Lmo1571	6-phosphofructokinase, Pfk	CP	-	-	mb/sn		[2,3]
Lmo1572	Acetyl-CoA carboxylase, α subunit	CP	-	-	mb		[3]
Lmo1580	Universal stress protein, UspA, Adenine nucleotide α hydrolases-like	CP	-	-	mb/sn		[2,3]
Lmo1600	Phospho-2-dehydro-3-deoxyheptonate aldolase, subtype 2	CP	-	-	mb		[3]
Lmo1606	DNA segregation ATPase FtsK/SpoIIIE	CP	-	-	mb		[3]
Lmo1611	Peptidase M42	CP	-	-	mb		[3]
Lmo1634	Bifunctional aldehyde/alcohol dehydrogenase, listerial adhesion protein, Lap	CP	-	-	se	SecA2	[7,29]
Lmo1636	ABC-type multidrug transport system, ATPase component	CP	-	-	mb		[3]
Lmo1641	Aconitase/iron regulatory protein 2	CP	-	-	mb		[3]
Lmo1647	Phospholipid/glycerol acyltransferase	CP	-	-	mb		[3]
Lmo1657	Translation elongation factor Ts	CP	-	-	mb/se/sn		[2,3,7]
Lmo1658	30S ribosomal protein S2	CP	-	-	mb		[3]
Lmo1713	Cell shape determining protein MreB/Mrl	CP	-	-	mb		[3]
Lmo1737	Glycerol dehydrogenase	CP	-	-	mb		[3]
Lmo1739	ABC-type polar amino acid transport system, ATPase component	CP	-	-	mb		[3]
Lmo1744	Nucleoside-diphosphate-sugar epimerases	CP	-	-	mb		[3]
Lmo1755	Glutamyl-tRNA amidotransferase subunit A, GatA	CP	-	-	mb		[3]
Lmo1764	Phosphoribosylglycinamide synthetase	CP	-	-	mb		[3]
Lmo1765	Phosphoribosylaminoimidazolecarboxamide formyltransferase, PurH	CP	-	-	mb/sn		[2,3]
Lmo1769	Phosphoribosylformylglycinamidine synthase II	CP	-	-	mb		[3]
Lmo1773	Adenylosuccinate lyase, PurB	CP	-	-	mb/sn		[2,3]
Lmo1774	Phosphoribosylaminoimidazole carboxylase, ATPase subunit	CP	-	-	mb		[3]

Lmo1775	1-(5-Phosphoribosyl)-5-amino-4-imidazole-carboxylate carboxylase, PurE	CP	-	-	sn		[2]
Lmo1783	50S ribosomal protein L20	CP	-	-	mb		[3]
Lmo1809	Phospholipid biosynthesis protein, PlsX type	CP	-	-	mb		[3]
Lmo1829	Fibronectin-binding protein A, FbpA, with DUF814	CP	-	-	mb	SecA2	[30]
Lmo1835	Carbamoyl phosphate synthase, large subunit, glutamine-dependent	CP	-	-	mb		[3]
Lmo1849	ABC-type Mn/Zn transport system, ATPase component	CP	-	-	mb		[3]
Lmo1867	Phosphoenolpyruvate synthase/pyruvate phosphate dikinase	CP	-	-	sn		[2]
Lmo1875	ABC-type sugar transport system, ATPase component	CP	-	-	mb		[3]
Lmo1877	Formyltetrahydrofolate synthetase	CP	-	-	mb		[3]
Lmo1934	Histone-like bacterial DNA-binding protein	CP	-	-	sn		[2]
Lmo1964	ABC-type multidrug transport system, ATPase component	CP	-	-	mb		[3]
Lmo1967	Toxic anion resistance protein, TelA type	CP	-	-	mb/se		[3,7]
Lmo2016	Cold-shock protein, DNA-binding	CP	-	-	sn		[2]
Lmo2020	Cell division initiation protein, DivIVA	CP	-	-	mb		[3]
Lmo2021	Small molecule binding protein with 3H domain	CP	-	-	se		[7]
Lmo2030	Protein of unknown function with DUF552 domain, COG1799	CP	-	-	mb		[3]
Lmo2032	Cell division protein, FtsZ	CP	-	-	mb		[3]
Lmo2033	Cell division protein, FtsA	CP	-	-	mb		[3]
Lmo2035	N-acetylglucosaminyltransferase, MurG	CP	-	-	mb		[3]
Lmo2068	Chaperonin GroEL	CP	-	-	mb/se/sn	SecA2	[1,2,3,7,8]
Lmo2069	Chaperonin GroES	CP	-	-	sn		[1,2]
Lmo2103	Phosphotransacetylase, Pta	CP	-	-	sn		[2]
Lmo2110	Mannose-6-phosphate isomerase	CP	-	-	se	SecA2	[8]
Lmo2114	ABC-type antimicrobial peptide transport system, ATPase	CP	-	-	mb		[3]
Lmo2118	Phosphoglucosamine mutase	CP	-	-	mb		[3]
Lmo2139	ABC-type multidrug transport system, ATPase	CP	-	-	mb		[3]
Lmo2154	Ribonucleotide-diphosphate reductase, β subunit	CP	-	-	mb		[3]
Lmo2192	ABC-type oligopeptide transport system, ATPase	CP	-	-	mb		[3]
Lmo2193	ABC-type dipeptide/oligopeptide/nickel transport system, ATPase	CP	-	-	mb		[3]
Lmo2206	Chaperonin ClpB	CP	-	-	mb		[3]
Lmo2215	ABC-type multidrug transport system, ATPase	CP	-	-	mb		[3]
Lmo2248	Phosphate transport regulator, distant homolog of PhoU	CP	-	-	mb		[3]
Lmo2296	Bacteriophage coat protein	CP	-	-	sn		[2]
Lmo2323	Protein of unknown function	CP	-	-	mb		[3]
Lmo2332	Resolvase	CP	-	-	mb		[3]
Lmo2372	ABC-type antimicrobial peptide transport system, ATPase	CP	-	-	mb		[3]
Lmo2389	NADH dehydrogenase, FAD-containing subunit	CP	-	-	mb		[3]

Lmo2391	Nucleoside-diphosphate-sugar epimerase	CP	-	-	mb		[3]
Lmo2411	ABC-type transport system involved in Fe-S cluster assembly, permease, SufB	CP	-	-	mb		[3]
Lmo2414	ABC-type transport system involved in Fe-S cluster assembly, permease	CP	-	-	mb		[3]
Lmo2415	SUF system FeS cluster assembly, SufC, ATPase	CP	-	-	mb/se		[3,7]
Lmo2419	ABC-type metal ion transport system, ATPase	CP	-	-	mb		[3]
Lmo2425	Glycine cleavage H-protein, GcvH	CP	-	-	se/sn		[2,7]
Lmo2455	Enolase	CP	-	-	mb/se/sn	SecA2	[1,2,3,7,8]
Lmo2456	Phosphoglycerate mutase, 2,3-bisphosphoglycerate-independent	CP	-	-	mb/sn		[1,3,7]
Lmo2458	Phosphoglycerate kinase	CP	-	-	se/sn		[1,2,7]
Lmo2459	Glyceraldehyde-3-phosphate dehydrogenase	CP	-	-	mb/se/sn		[1,2,3,7]
Lmo2468	Peptidase S14, ClpP	CP	-	-	mb		[3]
Lmo2507	Cell division ATP-binding protein, FtsE	CP	-	-	mb		[3]
Lmo2510	Sec translocase, ATPase, SecA	CP	-	-	mb		[3]
Lmo2511	Ribosomal protein S30Ae/σ54 modulation protein	CP	-	-	se/sn		[1,7]
Lmo2525	Cell shape determining protein MreB/Mrl	CP	-	-	mb		[3]
Lmo2528	F_0F_1 ATP synthase, subunit ε	CP	-	-	mb		[3]
Lmo2529	F_0F_1 ATP synthase, subunit β	CP	-	-	mb		[3]
Lmo2530	F_0F_1 ATP synthase, subunit γ	CP	-	-	mb		[3]
Lmo2531	F_0F_1 ATP synthase, subunit α	CP	-	-	mb		[3]
Lmo2532	F_0F_1 ATP synthase, subunit δ	CP	-	-	mb		[3]
Lmo2539	Glycine/serine hydroxymethyltransferase, GlyA	CP	-	-	mb/sn		[2,3]
Lmo2554	Glycosyl transferase, family 1	CP	-	-	mb		[3]
Lmo2556	Fructose/tagatose/ketose bisphosphate aldolase class II, FbaA	CP	-	-	se		[7]
Lmo2557	Sphingosine kinase	CP	-	-	mb		[3]
Lmo2580	ABC-type peptide transport system, ATPase	CP	-	-	mb		[3]
Lmo2596	30S ribosomal protein S9	CP	-	-	mb/se	SecA2	[3,8]
Lmo2597	50S ribosomal protein L13, RplM	CP	-	-	mb		[3]
Lmo2600	ABC-type cobalt transport system, ATPase	CP	-	-	mb		[3]
Lmo2601	ABC-type cobalt transport system, ATPase	CP	-	-	mb		[3]
Lmo2605	50S ribosomal protein L17	CP	-	-	mb		[3]
Lmo2613	50S ribosomal protein L15	CP	-	-	mb		[3]
Lmo2615	30S ribosomal protein S5	CP	-	-	mb		[3]
Lmo2618	30S ribosomal protein S8	CP	-	-	mb		[3]
Lmo2620	50S ribosomal protein L5	CP	-	-	mb		[3]
Lmo2626	30S ribosomal protein S3	CP	-	-	mb		[3]
Lmo2627	50S ribosomal protein L22	CP	-	-	mb		[3]
Lmo2629	50S ribosomal protein L2, RplB	CP	-	-	mb		[3]

Lmo2631	50S ribosomal protein L4	CP	-	-	mb/sn		[2,3]
Lmo2632	50S ribosomal protein L3	CP	-	-	mb		[3]
Lmo2653	Elongation factor EF-Tu, TufA	CP	-	-	mb/se/sn	SecA2	[2,3,7,8]
Lmo2654	Translation elongation factor EFG/EF2	CP	-	-	mb/se		[3,7]
Lmo2756	DNA topoisomerase III, TopB	CP	-	-	mb		[3]
Lmo2758	Inositol-5-monophosphate dehydrogenase	CP	-	-	mb		[3]
Lmo2781	Glycoside hydrolase	CP	-	-	mb		[3]
Lmo2785	Catalase, Kat	CP	-	-	mb		[3]
Lmo2792	λ repressor-like, DNA-binding domain	CP	-	-	mb		[3]
Lmo2795	DNA-binding transcriptional regulator with SIS domain	CP	-	-	ec		[2]
Lmo2853	RNA-binding protein	CP	-	-	mb		[3]

[a]Some annotations were corrected respective to the homology search performed as described in the Methods section. More extensive and detailed annotations are available in Table 1S.

[b]Besides exoproteins, parietal proteins (CW-protein) could be categorized between those covalently anchored to the cell wall via a LPXTG domain (LPXTG) and those attached via non-covalent interaction, namely with GW repeat, LysM, WXL or PGBD1 domains. Considering integral membrane protein (IMP), multi-spanning IMP (msIMP) are discriminated from single-spanning IMP (ssIMP), which are further categorized into (i) Type I (ssIMP I), *i.e.* IMP exhibiting a cleavable SP in addition to another TMD positioned upstream, (i) Type II (ssIMP II), *i.e.* IMP exhibiting a signal anchor, which is a TMD with N_{in}-C_{out} orientation (Type I module), that could be an uncleaved SP (Unc), (iii) Type III (ssIMP III), *i.e.* IMP exhibiting an reverse signal-anchor, which is a TMD with N_{out}-C_{in} orientation (Type II module). Proteins exhibiting a lipobox were predicted as lipoproteins (Lipo) anchored to cytoplasmic membrane.

[c]Subcellular location (SCL) follows the GO (Gene Ontology) for cellular component: cytoplasm (CP; GO:0005737), intrinsic to membrane (MB; GO:0031226), cell wall (CW; GO:0009275), cell surface (CS; GO:0009986) and extracellular milieu (EC; GO:0005576).

[d]The predicted routes follow by the secreted proteins are detailed in terms of (i) translocation system, namely Sec translocon, ABC exporter or holin, as well as membrane integration via YidC or secretion via non-classical way (NC), (ii) post-translocational modification, namely cleavage by signal peptidase of Type I (SPase I) or II (SPase II), protein acylation by the prolipoprotein diacylglyceryltransferase Lgt, covalent anchoring to cell wall by sortase A (SrtA) or B (SrtB), (iii) presence of a glycine residue at position +2 from the cleavage site (C+2=G).

[e]The signal peptide (SP) are differentiated between SP of Type I (I), Type II (II), signal anchor (SA), *i.e.* uncleaved signal peptide, or SP targeting protein to ABC exporter. The position +1 from the cleavage site (C+1) is given in bracket.

[f]Whenever possible, fraction of bacterial cell where the protein was experimentally identified follows the GO (Gene Ontology) for cellular component: membrane fraction (mb; GO:005624), cell wall fraction (cw), surface exposed fraction (se) and supernatant (sn). Asterisk indicates protein identified in supernatant of an isogenic mutant for the Lgt maturation pathway resulting in the release of lipoprotein into the extracellular milieu [25].

[g]Experimental information on the secretion route is provided for SecA2-dependent protein secretion, SP cleavage by signal peptidase of Type I (SPase I) SipX and/or SipZ, or signal peptidase of Type II (SPase II) LspA, covalent protein anchoring to cell wall by sortase A (SrtA) or B (SrtB), non-covalent attchment to cell wall *via* GW repeats, or protein anchoring to the cytoplasmic membrane by the prolipoprotein diacylglyceryltransferase Lgt.

[h]Experimental identification of cleavable SP is provided with the position +1 from the cleavage site (C+1).

REFERENCES

1. Desvaux M, Dumas E, Chafsey I, Chambon C, Hebraud M (2010) Comprehensive appraisal of the extracellular proteins from a monoderm bacterium: theoretical and empirical exoproteomes of *Listeria monocytogenes* EGD-e by secretomics. J Proteome Res 9: 5076-5092.
2. Trost M, Wehmhoner D, Karst U, Dieterich G, Wehland J, et al. (2005) Comparative proteome analysis of secretory proteins from pathogenic and nonpathogenic *Listeria* species. Proteomics 5: 1544-1557.
3. Wehmhoner D, Dieterich G, Fischer E, Baumgartner M, Wehland J, et al. (2005) "LaneSpector", a tool for membrane proteome profiling based on sodium dodecyl sulfate-polyacrylamide gel electrophoresis/liquid chromatography-tandem mass spectrometry analysis: application to *Listeria monocytogenes* membrane proteins. Electrophoresis 26: 2450-2460.
4. Bonnemain C, Raynaud C, Reglier-Poupet H, Dubail I, Frehel C, et al. (2004) Differential roles of multiple signal peptidases in the virulence of *Listeria monocytogenes*. Mol Microbiol 51: 1251-1266.
5. Lety MA, Frehel C, Beretti JL, Berche P, Charbit A (2003) Modification of the signal sequence cleavage site of listeriolysin O does not affect protein secretion but impairs the virulence of *Listeria monocytogenes*. Microbiology 149: 1249-1255.
6. Garcia-Del Portillo F, Calvo E, D'Orazio V, Pucciarelli MG (2011) Association of ActA to the peptidoglycan revealed by cell wall proteomics of intracellular *Listeria monocytogenes*. J Biol Chem.
7. Schaumburg J, Diekmann O, Hagendorff P, Bergmann S, Rohde M, et al. (2004) The cell wall subproteome of *Listeria monocytogenes*. Proteomics 4: 2991-3006.
8. Lenz LL, Mohammadi S, Geissler A, Portnoy DA (2003) SecA2-dependent secretion of autolytic enzymes promotes *Listeria monocytogenes* pathogenesis. Proc Natl Acad Sci U S A 100: 12432-12437.
9. Pucciarelli MG, Calvo E, Sabet C, Bierne H, Cossart P, et al. (2005) Identification of substrates of the *Listeria monocytogenes* sortases A and B by a non-gel proteomic analysis. Proteomics: 10.1002/pmic.200402075.
10. Calvo E, Pucciarelli MG, Bierne H, Cossart P, Albar JP, et al. (2005) Analysis of the *Listeria* cell wall proteome by two-dimensional nanoliquid chromatography coupled to mass spectrometry. Proteomics 5: 433-443.

11. Personnic N, Bruck S, Nahori MA, Toledo-Arana A, Nikitas G, et al. (2010) The stressinduced virulence protein InlH controls interleukin-6 production during murine listeriosis. Infect Immun 78: 1979-1989.

12. Cabanes D, Sousa S, Cebria A, Lecuit M, Garcia-del Portillo F, et al. (2005) Gp96 is a receptor for a novel *Listeria monocytogenes* virulence factor, Vip, a surface protein. Embo J 24: 2827-2838.

13. Garandeau C, Reglier-Poupet H, Dubail I, Beretti JL, Berche P, et al. (2002) The sortase SrtA of *Listeria monocytogenes* is involved in processing of internalin and in virulence. Infect Immun 70: 1382-1390.

14. Gaillard JL, Berche P, Frehel C, Gouin E, Cossart P (1991) Entry of *Listeria monocytogenes* into cells is mediated by internalin, a repeat protein reminiscent of surface antigens from Gram-positive cocci. Cell 65: 1127-1141.

15. Dortet L, Mostowy S, Louaka AS, Gouin E, Nahori MA, et al. (2011) Recruitment of the major vault protein by InlK: a *Listeria monocytogenes* strategy to avoid autophagy. PLoS Pathog 7: e1002168.

16. Bierne H, Garandeau C, Pucciarelli MG, Sabet C, Newton S, et al. (2004) Sortase B, a new class of sortase in *Listeria monocytogenes*. J Bacteriol 186: 1972-1982.

17. Newton SM, Klebba PE, Raynaud C, Shao Y, Jiang X, et al. (2005) The *svpA-srtB* locus of *Listeria monocytogenes*: Fur-mediated iron regulation and effect on virulence. Mol Microbiol 55: 927-940.

18. Borezee E, Pellegrini E, Beretti JL, Berche P (2001) SvpA, a novel surface virulence-associated protein required for intracellular survival of *Listeria monocytogenes*. Microbiology 147: 2913-2923.

19. Sabet C, Toledo-Arana A, Personnic N, Lecuit M, Dubrac S, et al. (2008) The *Listeria monocytogenes* virulence factor InlJ is specifically expressed in vivo and behaves as an adhesin. Infect Immun 76: 1368-1378.

20. Braun L, Dramsi S, Dehoux P, Bierne H, Lindahl G, et al. (1997) InlB: an invasion protein of *Listeria monocytogenes* with a novel type of surface association. Mol Microbiol 25: 285-294.

21. Milohanic E, Jonquieres R, Glaser P, Dehoux P, Jacquet C, et al. (2004) Sequence and binding activity of the autolysin-adhesin Ami from epidemic *Listeria monocytogenes* 4b. Infect Immun 72: 4401-4409.

22. Cabanes D, Dussurget O, Dehoux P, Cossart P (2004) Auto, a surface associated autolysin of *Listeria monocytogenes* required for entry into eukaryotic cells and virulence. Mol Microbiol 51: 1601-1614.

23. Ruhland GJ, Hellwig M, Wanner G, Fiedler F (1993) Cell-surface location of *Listeria*-specific protein p60--detection of *Listeria* cells by indirect immunofluorescence. J Gen Microbiol 139: 609-616.

24. Kuhn M, Goebel W (1989) Identification of an extracellular protein of *Listeria monocytogenes* possibly involved in intracellular uptake by mammalian cells. Infect Immun 57: 55-61.

25. Baumgärtner M, Kärst U, Gerstel B, Loessner M, Wehland J, et al. (2007) Inactivation of Lgt allows systematic characterization of lipoproteins from *Listeria monocytogenes*. J Bacteriol 189: 313-324.

26. Reglier-Poupet H, Pellegrini E, Charbit A, Berche P (2003) Identification of LpeA, a PsaA-like membrane protein that promotes cell entry by *Listeria monocytogenes*. Infect Immun 71: 474-482.

27. Reglier-Poupet H, Frehel C, Dubail I, Beretti JL, Berche P, et al. (2003) Maturation of lipoproteins by type II signal peptidase is required for phagosomal escape of *Listeria monocytogenes*. J Biol Chem 278: 49469-49477.

28. Archambaud C, Nahori MA, Pizarro-Cerda J, Cossart P, Dussurget O (2006) Control of *Listeria* superoxide dismutase by phosphorylation. J Biol Chem 281: 31812-31822.

29. Burkholder KM, Kim KP, Mishra KK, Medina S, Hahm BK, et al. (2009) Expression of LAP, a SecA2-dependent secretory protein, is induced under anaerobic environment. Microbes Infect 11: 859-867.

30. Dramsi S, Bourdichon F, Cabanes D, Lecuit M, Fsihi H, et al. (2004) FbpA, a novel multifunctional *Listeria monocytogenes* virulence factor. Mol Microbiol 53: 639-649.
